# *Actl7b* deficiency leads to mislocalization of LC8 type dynein light chains and disruption of murine spermatogenesis

**DOI:** 10.1242/dev.201593

**Published:** 2023-10-27

**Authors:** Gina E. Merges, Lena Arévalo, Andjela Kovacevic, Keerthika Lohanadan, Dirk G. de Rooij, Carla Simon, Melanie Jokwitz, Walter Witke, Hubert Schorle

**Affiliations:** ^1^ Department of Developmental Pathology, Institute of Pathology, University Hospital Bonn, 53127 Bonn, Germany; ^2^ Department of Molecular Cell Biology, Institute for Cell Biology, University of Bonn, 53121 Bonn, Germany; ^3^ Reproductive Biology Group, Division of Developmental Biology, Department of Biology, Faculty of Science, Utrecht University, 3584 CH Utrecht, The Netherlands; ^4^ Cell Migration Unit, Institute of Genetics, University of Bonn, 53115 Bonn, Germany

**Keywords:** *Actl7b*, Infertility, Spermatogenesis, Actin-related proteins, LC8 light chains, Mouse

## Abstract

Actin-related proteins (Arps) are classified according to their similarity to actin and are involved in diverse cellular processes. ACTL7B is a testis-specific Arp, and is highly conserved in rodents and primates. ACTL7B is specifically expressed in round and elongating spermatids during spermiogenesis. Here, we have generated an *Actl7b*-null allele in mice to unravel the role of ACTL7B in sperm formation. Male mice homozygous for the *Actl7b*-null allele (*Actl7b^−/−^*) were infertile, whereas heterozygous males (*Actl7b^+/−^*) were fertile. Severe spermatid defects, such as detached acrosomes, disrupted membranes and flagella malformations start to appear after spermiogenesis step 9 in *Actl7b^−/−^* mice, finally resulting in spermatogenic arrest. Abnormal spermatids were degraded and levels of autophagy markers were increased. Co-immunoprecipitation with mass spectrometry experiments identified an interaction between ACTL7B and the LC8 dynein light chains DYNLL1 and DYNLL2, which are first detected in step 9 spermatids and mislocalized when ACTL7B is absent. Our data unequivocally establish that mutations in ACTL7B are directly related to male infertility, pressing for additional research in humans.

## Teaser

**Summary:** *Actl7b*-deficient mice display spermatogenic arrest, which is likely due to loss of the interaction of ACTL7B with DYNLL1/DYNLL2, a spermatid cytoskeleton protein.

## INTRODUCTION

Proteins belonging to the superfamily of actin-like/actin-related proteins share up to 60% amino acid identity with conventional actins and act in various cellular processes, including vesicle trafficking, chromatin modulation, microtubule motility and actin filament dynamics (Schafer and Schroer, 1999).

*ACTL7B* was first described by Chadwick et al. (1999), who identified and characterized two previously unreported actin-like genes, *ACTL7A* and *ACTL7B* (previously known as *T-ACTIN 2* and *T-ACTIN 1*), from the familial dysautonomia candidate region on chromosome 9q31 in human. However, neither gene was found to be mutated in individuals with dysautonomia, suggesting that they are not involved in its pathogenesis. Nucleotide alignment showed high level identity between these two genes and a greater than 40% predicted amino acid identity to a variety of actin proteins. In mice, *Actl7a* and *Actl7b* were mapped to chromosome 4 (Chadwick et al., 1999; Hisano et al., 2003). It has been proposed these genes arose before the divergence of rodents and primates by retropositioning of a spliced mRNA transcribed from an actin progenitor gene (Hisano et al., 2003). In mouse and human, *ACTL7B* is expressed exclusively in the testis (Tanaka et al., 2003; Hisano et al., 2003), suggesting a role in spermatogenesis.

In mouse and human, *ACTL7B* has been found to be expressed post-meiotically in round and elongating spermatids (Hisano et al., 2003; Guo et al., 2018). ACTL7B is detected in the cytoplasm and at lesser amounts in the nucleus of round and elongating spermatids; it seems to be, in contrast to ACTL7A, evicted with excess cytoplasm at the end of spermiogenesis (Tanaka et al., 2003).

Interestingly, five polymorphisms in *ACTL7B* (and six in *ACTL7A*) were detected in a cohort of Japanese infertile male individuals, suggesting that *ACTL7B* plays a role in fertility (Tanaka et al., 2019). Noteworthy, a study comparing two groups of Luzhong mutton sheep with different fecundity identified nine genes, among these *ACTL7B* (and *ACTL7A*), that were associated with reduced litter size (Tao et al., 2021). Furthermore, proteomic and phosphoproteomic analysis of prepubertal and pubertal testis of swamp buffalo identified ACTL7B to be more abundant and phosphorylated in the pubertal testis, again suggesting a role in spermatogenesis (Huang et al., 2020). Single nucleotide polymorphisms in the coding sequence of *ACTL7B* in infertile men have been reported; however, they have not been directly correlated to male infertility (Tanaka et al., 2007, 2019). Furthermore, a study comparing the expression of testis-enriched genes in fertile and teratozoospermic men identified *ACTL7B* to be among those genes expressed at significantly lower levels in teratozoospermic men (Ahn et al., 2017). Additionally, a recent study, using comparative proteomics on human testicular tissue, identified ACTL7B, both at the protein and mRNA level, among the six proteins/transcripts with the highest discriminating power of obstructive and non-obstructive azoospermia subtypes (Davalieva et al., 2022).

The molecular function of ACTL7B is not fully understood, although its immunolocalization suggests a role in cytoskeletal organization and/or protein trafficking during spermatogenesis. A recent study by Clement et al. described the generation and analysis of *Actl7b*-null mice, (Clement et al., 2023). Male *Actl7b*-null mice are infertile and show severe oligoteratozoospermia with malformations of the sperm tails and heads.

Here, we have generated an *Actl7b*-null allele using CRISPR/Cas9-mediated gene editing to investigate the role of ACTL7B in more detail. While *Aclt7b^+/−^* males were unaffected, *Actl7b^−/−^* males were infertile, showing morphological sperm abnormalities as described previously (Clement et al., 2023). Spermatogenic abnormalities arose after step 9 of spermiogenesis, leading to disruption of sperm differentiation as well as spermatid phagocytosis and degradation. Co-immunoprecipitation and mass spectrometry analyses revealed interaction of ACTL7B with dynein light chains DYNLL1 and DYNLL2. Loss of ACTL7B leads to mislocalization of DYNLL1 and DYNLL2 in spermatids of *Actl7b^−/−^* males highlighting its role in cytoskeletal re-organization.

## RESULTS

### Generation of *Actl7b*-deficient mice

**Fig. 1.**
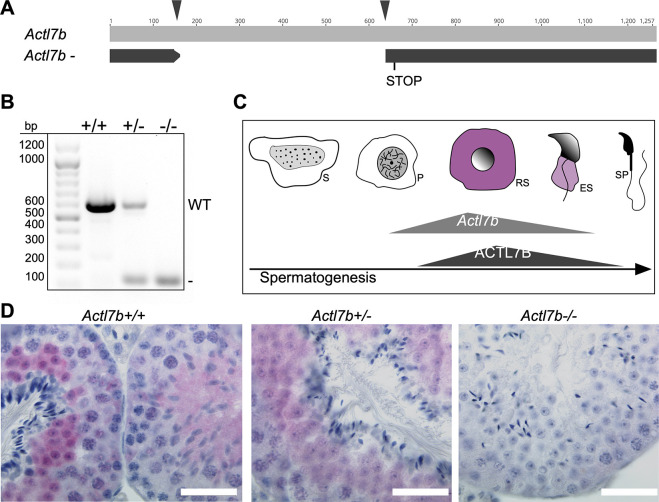
**Establishment of *Actl7b*-deficient mice.** (A) Graphical representation of CRISPR-Cas9-mediated gene editing of the *Actl7b* locus using two guide RNAs (black arrowheads) targeting the intron-less *Actl7b*-coding sequence. 473 bp were deleted, causing a frameshift leading to a premature stop. (B) Agarose gel of genotyping polymerase chain reaction of *Actl7b^+/+^*, *Actl7b^+/−^* and *Actl7b^−/−^* mice (wild-type band, 607 bp; KO band, 134 bp). (C) *Actl7b* expression and ACTL7B immunolocalization during spermiogenesis based on literature (Hisano et al., 2003; Tanaka et al., 2003; Guo et al., 2018). ES, elongating spermatids; P, pachytene spermatocytes; RS, round spermatids; S, spermoatogonia; SP, spermatozoa. (D) Immunohistochemical staining against ACTL7B on Bouin-fixed, paraffin wax-embedded *Actl7b^+/+^*, *Actl7b^+/−^* and *Actl7b^−/−^* testis sections counterstained with Hematoxylin. Scale bars: 20 μm.

We applied CRSIPR/Cas9-mediated gene editing in zygotes to generate *Actl7b*-deficient mice. Two guides were used targeting the intron-less coding sequence of *Actl7b* (Fig. 1A, arrowheads). Founders were backcrossed to C57BL/6J mice and the *Actl7b* locus was sequenced in the F1 generation. A mouse carrying a 473 bp deletion causing a frameshift was selected to establish an *Actl7b-*deficient line and animals were analyzed starting from generation N2. A genotyping PCR was established to discriminate the *Actl7bΔ* from the wild-type allele (Fig. 1B). *Actl7b* was described to be expressed in round and elongating spermatids in mouse and human (Hisano et al., 2003; Guo et al., 2018; Lukassen et al., 2018) (Fig. 1C, Fig. S1).

We used testis sections from heterozygous (*Actl7b^+/−^*), homozygous (*Actl7b^−/−^*) and wild-type mice (*Actl7b^+/+^*) for immunohistochemical staining against ACTL7B. We demonstrate that ACTL7B localizes to the cytoplasm of round and elongating spermatids in wild-type and heterozygous animals, confirming published data (Tanaka et al., 2003) (Fig. 1D). Of note, the staining is intense in round spermatids and weakens as spermatids elongate. In testis sections of *Actl7b^+/−^* mice, ACTL7B signal appeared weaker. No staining was detected in *Actl7b^−/−^* testis sections, validating the null allele.

### *Actl7b* deficiency leads to a disruption of spermatogenesis and infertility in male mice

**Fig. 2.**
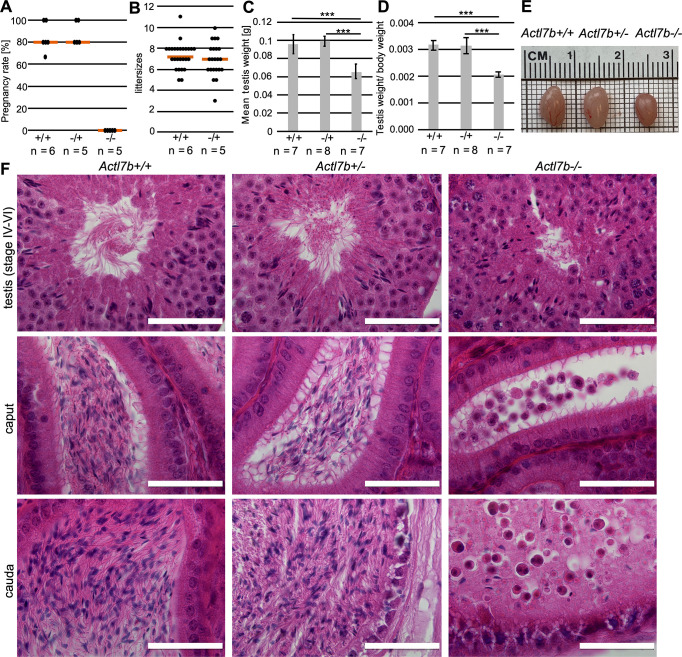
**Fertility analysis and reproductive organ morphology.** (A) Pregnancy rate of *Actl7b^+/+^*, *Actl7b^+/−^* and *Actl7b^−/−^* males mated with female wild-type C57BL/6J mice (*n*=number of males). (B) Average litter sizes monitored after mating of *Actl7b^+/+^* and *Actl7b^+/−^* males with female wild-type C57BL/6J mice (*n*=number of males). Five plugs per male were recorded. (C) Mean testis weight of *Actl7b^+/+^*, *Actl7b^+/−^* and *Actl7b^−/−^* males (*n*=number of males). (D) Testis to body weight ratio of *Actl7b^+/+^*, *Actl7b^+/−^* and *Actl7b^−/−^* males (*n*=number of males). (E) Photographs of representative testes dissected from *Actl7b^+/+^*, *Actl7b^+/−^* and *Actl7b^−/−^* littermates with similar body weight. (F) Hematoxylin and Eosin staining of testis (stages IV-VI of the epithelial cycle), caput epididymis and cauda epididymis of *Actl7b^+/+^*, *Actl7b^+/−^* and *Actl7b^−/−^* males. Scale bars: 50 μm. Statistical analyses were carried out using a two-tailed, unpaired Student's *t*-test (***P<0.001). Error bars represent s.d.

Fertility analysis revealed that *Actl7b^+/−^* males produce similar litter sizes and pregnancy frequencies to *Actl7b^+/+^* males (Fig. 2A,B), whereas *Actl7b^−/−^* males are infertile. Macroscopic analysis of the reproductive organs showed that testis weight, testis to body weight ratio and testis size of *Actl7b^−/−^* males is significantly reduced compared with *Actl7b^+/−^* and *Actl7b^+/+^* males (Fig. 2C-E), indicating defects in spermatogenesis. Similar results described by Clement et al. were that homozygous male mice were infertile and showed an approximately 20% reduction in testis weight compared with wild-type controls (Clement et al., 2023).

Next, histological sections of testis, caput and cauda epididymis were prepared. Sperm production appears normal in *Actl7b^+/−^* males (Fig. 2F). *Actl7b^+/−^* testis sections containing step 13-15 spermatids do not significantly differ from wild-type testis sections, and epididymides contain mature, morphologically normal sperm. In contrast, spermatogenesis in *Actl7b^−/−^* males appears to be disrupted. Caput and cauda epididymides are filled with cell debris, morphologically abnormal spermatids and roundish cells most likely representing immature germ cells (Fig. 2F). Similar observations have been made by Clement et al., who describe the presence of cell debris and many roundish cells with the appearance of round spermatids in cauda epididymides of *Actl7b*-KO males (Clement et al., 2023). These cells were described to be TUNEL positive, i.e. apoptotic.

Differences between cauda epididymides of wild-type and *Actl7b^−/−^* males were already apparent upon inspecting the dissected organs (Fig. S2A). Cauda from *Actl7b^−/−^* males appeared smoother and less filled. We isolated sperm from the cauda epididymis from *Actl7b^+/+^*, *Actl7b^+/−^* and *Actl7b^−/−^* males via swim-out. Sperm count was severely reduced in *Actl7b^−/−^* with an average of 32,250 sperm from both cauda epididymides (Fig. S2B,C). This corresponds to a 1000-fold reduction in sperm number and differs from the results of Clement et al., who showed an ∼10-fold reduction (Clement et al., 2023). Sperm count was not significantly different between *Actl7b^+/−^* and wild-type males. *Actl7b^+/−^* sperm appear morphologically normal (Fig. S2D,E), viable (Fig. S2F) and motile (not shown). Although *Actl7b^−/−^* males show a pathomorphological phenotype, loss of one allele of *Actl7b* seems to be phenotypically inconspicuous. In *Actl7b^−/−^* mice, daily sperm production (Fig. S2F) and the number of elongating spermatids per seminiferous tubule cross-section (Fig. S2G) were significantly reduced, indicating that the reduction in spermatids originates at least partially from defective spermiogenesis in the testis.

**Fig. 3.**
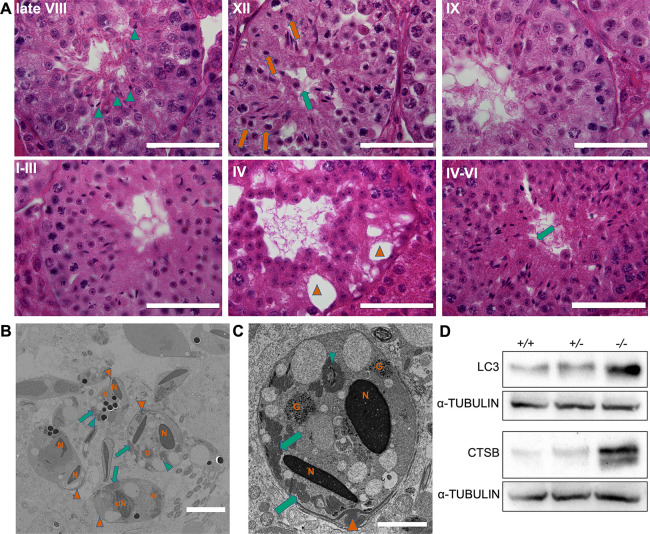
**Morphology of *Actl7b*-deficient seminiferous tubules.** (A) Hematoxylin and Eosin staining of Bouin-fixed paraffin wax-embedded testis sections of *Actl7b^−/−^* mice. Immature apoptotic germ cells can be seen to be released into the lumen (green arrows). At late stage VIII, elongated spermatids with an abnormal morphology, which were not spermiated, were seen (green arrowheads) and round spermatids blocked in development with dark cytoplasm were found (orange arrows). Vacuolation of seminiferous tubules was detected (orange arrowheads). Scale bars: 50 µm. (B,C) Transmission electron micrographs of vesicles filled with degrading spermatids detected in *Actl7b^−/−^* seminiferous tubules. N, condensed nuclei; dN, degraded nucleus; G, granular material. Orange arrowheads indicate acrosomal structures; green arrowheads indicate flagellar cross-sections; green arrows indicate mitochondria. Scale bars: 5 µm in B; 2 µm in C. (D) Western blots on protein extractions from whole *Actl7b^+/+^*, *Actl7b^+/−^* and *Actl7b^−/−^* testis. Anti-LC3 and anti-CSTB were used. α-Tubulin was used as a loading control.

*Actl7b^−/−^* seminiferous tubules appear disorganized and germ cell development abnormal (Fig. 3A). Vacuolations in the seminiferous tubules are detected mostly in the basal region of the seminiferous tubules, indicating recent loss of germ cells. During stage VIII, retained abnormally formed elongated spermatids originating from the previous epithelial cycle can be seen. Importantly, we detected round spermatids that seem to be blocked in development and present with dark cytoplasm, indicative of apoptosis/degradation. Immature germ cells seem to be released into the lumen of seminiferous tubules. Clement et al. described the roundish cells being shed into the lumen of seminiferous tubules in their *Actl7b*-KO mouse model to be TUNEL positive (Clement et al., 2023). Here, we detected vesicles filled with degrading spermatids in *Actl7b^−/−^* seminiferous tubules (Fig. 3B,C). These contained condensed nuclei, acrosomal and tail structures, and mitochondria and granular material. This suggests that Sertoli cells have become engulfed and are degrading spermatids. However, TUNEL staining did not reveal significant differences (Fig. S2H). Of note, we found that levels of a mammalian autophagy marker, microtubule-associated protein 1A/1B-light chain 3 (LC3), and of cathepsin B (CTSB) significantly increased in *Act7b^−/−^* testis (Fig. 3D). Interestingly, deletion of CTSB in mice leads to inhibition of autophagy and promotion of apoptosis in murine testis, suggesting that CTSB is a regulator between autophagy and apoptosis during spermatid development (Wen et al., 2022). Hence, based on our data we favor autophagy as being the main cause of spermatid degradation.

Closer inspection of testicular sections revealed defects in synchronization of the epithelial cycle in *Actl7b^−/−^* mice. Cohorts of spermatids in stage IX start elongation but it appears they do not elongate in a synchronous manner (Fig. S3A). Spermatids do not condense properly and seem to localize too close to the basal lamina. Furthermore, cohorts of round spermatids appear abnormal and seem disorganized at stage IX (Fig. S3B). In comparison, wild-type stage IX tubules appear more synchronized. Next, at stage X, morphologically abnormal elongating spermatids are detected, whereas in wild-type stage X tubules, all elongating spermatids appear to be at the same stage and look normal (Fig. S3C). Finally, in stage VII seminiferous tubules of *Actl7b^−/−^* males, all elongating spermatids were morphologically abnormal (Fig. S3D). Compared with wild type, *Actl7b^−/−^* elongating spermatids do not align properly at the lumen and are very few in number. Substantial numbers of round spermatids are formed in *Actl7b^−/−^* testis and spermatogenesis appears normal until this stage. As expected, meiosis was inconspicuous in *Actl7b^−/−^* testis and no increased numbers of apoptotic divisions were detected (Fig. S3E).

### Spermiogenesis is disrupted in *Actl7b^−/−^* males, and *Actl7b^−/−^* spermatids show various structural defects

**Fig. 4.**
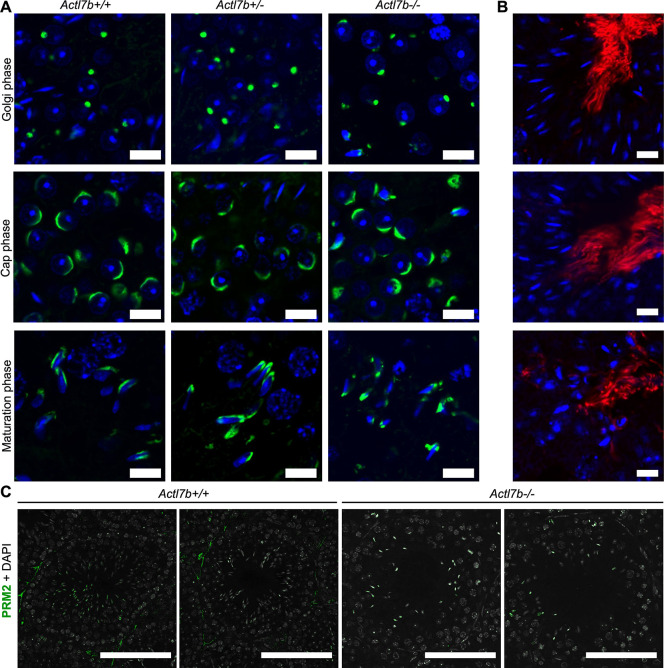
**Acrosome formation, flagella formation and chromatin condensation in *Actl7b*-deficient mice.** (A) PNA staining of testis of *Actl7b^+/+^*, *Actl7b^+/−^* and *Actl7b^−/−^* males. Acrosomal structures in Golgi, cap and maturation phases are shown. Scale bars: 10 μm. (B) Immunohistochemistry staining against ODF2 on *Actl7b^+/+^* (top) *Actl7b^+/−^* (middle) and *Actl7b^−/−^* (bottom) testis tissue sections. DAPI was used as the counterstain. Scale bars: 10 μm. (C) Immunohistochemistry staining against PRM2 on *Actl7b^+/+^*, *Actl7b^+/−^* and *Actl7b^−/−^* testis tissue sections. DAPI (in gray) was used as the counterstain. Scale bars: 50 μm.

To examine spermiogenesis in *Actl7b*-deficient males in more detail, we looked at basic parameters, including acrosome biogenesis, DNA condensation, manchette formation and sperm tail formation. Because ACTL7B is first present in round spermatids, effects were expected to manifest from round spermatid stage onwards. In comparison with *Actl7b^+/−^* and *Actl7b^+/+^*, in *Actl7b^−/−^* testis, acrosomal structures are less frequent and disorganized after spermatids start to elongate (Fig. 4A, Fig. S4A,B). In Golgi and Cap phase *Actl7b^−/−^* acrosomal structures appear normal, abnormalities become apparent in Maturation phase (Fig. 4A). A signal for ODF2 in the lumen of seminiferous tubules of *Actl7b^−/−^*, *Actl7b^+/−^* and *Actl7b^+/+^* testis sections indicated that sperm flagellar structures are formed (Fig. 4B, Fig. S4C). These are, however, less frequent and more often found in clusters close to the basal membrane, again suggesting engulfment and degradation of spermatids (Fig. S4C). Staining against PRM2 in the nuclei of elongating spermatids in *Actl7b^−/−^*, *Actl7b^+/−^* and *Actl7b^+/+^* testis sections indicates that nuclear remodeling and chromatin condensation is initiated (Fig. 4C). Transition proteins are loaded onto the DNA and, later, protamines are detected in the nuclei of spermatids (Fig. S5A,B). Staining against the cleaved domain of PRM2 (cP2) showed that PRM2 localizes to the nucleus of developing spermatids. Finally, in wild-type mice, the remaining full-length unprocessed PRM2 is evicted to the residual bodies (Fig. S5C), as described previously (Arévalo et al., 2022). In contrast, in *Actl7b^−/−^* testis sections, residual bodies seem enlarged and less clearly separated from spermatid nuclei. Seemingly, the eviction of cytoplasm fails in large parts of *Actl7b^−/−^* spermatids and cP2 filled cytoplasm is retained.

**Fig. 5.**
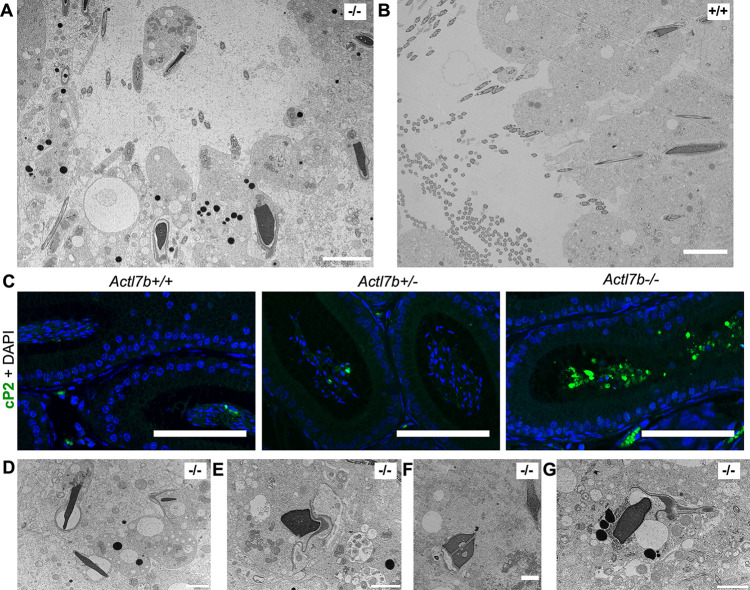
**Ultrastructural analysis of *Actl7b*^−/−^ testis.** (A) Transmission electron micrograph of a lumen of an *Actl7b^−/−^* seminiferous tubule. Scale bar: 5 µm. (B) Transmission electron micrograph of a lumen of an *Actl7b^+/+^* seminiferous tubule. Scale bar: 5 µm. (C) Immunohistochemistry against cP2 on caput sections from *Actl7b^+/+^*, *Actl7b^+/−^* and *Actl7b^−/−^* mice. DAPI was used as a counterstain. Scale bars: 100 µm. (D-G) Images of representative *Actl7b^−/−^* spermatids with condensed nuclei. Scale bars: 2 µm.

Transmission electron micrographs reveal that spermatids in *Actl7b^−/−^* testes show abnormal morphologies and excess cytoplasm, which is indicative of defective eviction of cytoplasm (Fig. 5A). In comparison, in the lumen of *Actl7b^+/+^* tubules morphologically normal sperm line up to be spermiated (Fig. 5B). Staining against cP2 (to visualize residual cytoplasm) on caput epididymal sections showed that large amounts of cP2 are retained in the cytoplasm of immature germ cells in *Actl7b^−/−^* mice (Fig. 5C). This, again, suggests defects in the eviction of cytoplasm. In later developmental stages of *Actl7b^−/−^* spermatids in the testis, sperm membranes and acrosomal structures become detached (Fig. 5D,E). Part of the condensed nuclei show inclusions (Fig. 5F) and the overall organization of elongating spermatids appears disorganized (Fig. 5G). Sperm-specific structures fail to assemble correctly. Staining of epididymal sperm with PNA and Mitotracker showed no significant differences between *Actl7b^+/−^* and wild-type sperm (Fig. S6A). In contrast, *Actl7b^−/−^* epididymal sperm show mislocalization of acrosomal structures and mitochondria. In most *Actl7b^−/−^* sperm, PNA and Mitotracker signals were found along the whole tail and in the head region. Of note, Clement et al. reported mislocalization of mitochondria in their *Actl7b*-KO model (Clement et al., 2023). Clement et al. further describe mislocalization of flagellar proteins in KO sperm and multiple morphological malformations of flagellae. Therefore, we used IC staining against α-tubulin to analyze manchette formation in *Actl7b^−/−^* spermatids. Manchettes appear irregular compared with wild type, and *Actl7b^−/−^* spermatid head shapes are abnormal (Fig. S6B).

When analyzing the single steps of spermiogenesis, the first defects become apparent after step 9 (Figs S7 and S8). In part of the developing *Actl7b^−/−^* spermatids, chromatin condensation seems to be initiated earlier than in wild-type spermatids. Darker stained chromatin could, however, also be a sign of DNA degradation. Irregular sperm head shaping becomes apparent in step 10 spermatids.

These results lead us to conclude that spermiogenesis is disrupted in *Actl7b^−/−^* males. However, the block in development seems to be heterogeneous. Although some germ cells arrest in development at round spermatid stage and appear to be degraded or are released into the lumen, others develop further, form more advanced acrosomal structures, and show hypercondensed chromatin and flagella formation. Apparently, key processes of sperm development are initiated, but seemingly run in an uncoordinated fashion. Finally, abnormally formed spermatids become engulfed and degraded.

### ACTL7B interacts with dynein light chains DYNLL1 and DYNLL2

To identify ACTL7B-protein interactions, anti-ACTL7B antibody was coupled to Dynabeads and used for co-immunoprecipitation on protein extracts from whole wild-type testes. Uncoupled beads were used as control. Eluted proteins were identified by mass spectrometry (Table S1). After excluding contaminating proteins such as keratins, proteins identified in the ‘beads only’ control were subtracted from the dataset. Furthermore, a published bead proteome from HeLa cells was used to filter out proteins that nonspecifically bind Dynabeads (Trinkle-Mulcahy et al., 2008). Those included various H2B histone variants. In the co-immunoprecipitation using the anti-ACTL7B-coupled beads, we identified LC8 light chains, dynein light chain 1 (DYNLL1) and its paralog dynein light chain 2 (DYNLL2). Other proteins identified were ribonucleoproteins and ribosomal proteins, which were excluded from further analysis.

**Fig. 6.**
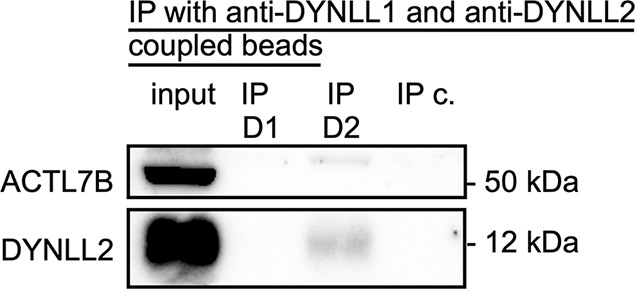
**Interaction of ACTL7B with DYNLL1 and DYNLL2.** Western blots of the protein input (whole wild-type testis), the IP eluate of the anti-DYNLL1-coupled beads (IP D1), the IP eluate of the anti-DYNLL2-coupled beads (IP D2) and the IP eluate of the beads-only control (IP c.). Anti-ACTL7B and anti-DYNLL2 antibodies were used for western blots.

We next performed co-immunoprecipitation using anti DYNLL1 and anti-DYNLL2 coupled beads (Fig. 6, Fig. S9). The anti-DYNLL1 antibody appeared not to be suitable for coupling and/or co-immunoprecipitation, as no DYNLL1 was detected in the eluate (not shown). In the eluate of the co-immunoprecipitation using anti-DYNLL2-coupled beads, DYNLL2 and ACTL7B were detected, further supporting the ACTL7B-DYNLL2 interaction.

Next, we generated HEK cells stably expressing *Actl7b* fused to *eGFP* (HEK*^Actl7b-eGFP^*) and performed a pull-down with GFP nanobody-coupled beads followed by mass spectrometry analysis on the eluate. Here, we identified ACTL7B and DYNLL1 enriched in the Actl7b sample using a targeted analysis (Table S2). Of note, DYNLL2 peptides were detected but they were either identical to DYNLL1 or the detection level was low.

**Fig. 7.**
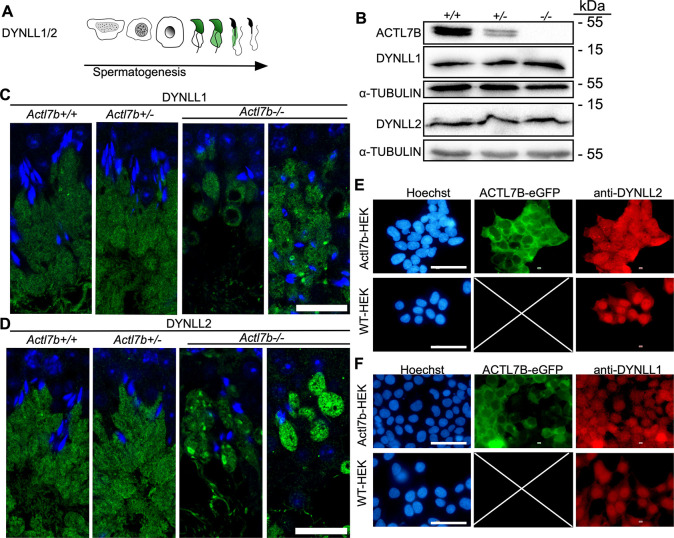
**Localization of DYNLL1 and DYNLL2 in *Actl7b*-deficient testis.** (A) Graphical depiction of DYNLL1 and DYNLL2 immunolocalization during spermiogenesis based on literature (Wang et al., 2005). (B) Western blots on protein extracts from whole *Actl7b^+/+^*, *Actl7b^+/−^* and *Actl7b^−/−^* testis. Anti-ACTL7B, anti-DYNLL2 and anti-DYNLL2 were used. α-Tubulin was used as loading control. (C) DYNLL1 staining in *Actl7b^+/+^*, *Actl7b^+/−^* and *Actl7b^−/−^* elongating spermatids. DAPI was used as a counterstain. Scale bar: 20 µm. (D) DYNLL2 staining in *Actl7b^+/+^*, *Actl7b^+/−^* and *Actl7b^−/−^* elongating spermatids. DAPI was used as a counterstain. Scale bar: 20 µm. (E) Immunocytochemical staining against DYNLL2 in wild-type and ACTL7B-eGFP-expressing HEK cells. Scale bars: 50 µm. (F) Immunocytochemical staining against DYNLL1 in wild-type and ACTL7B-eGFP-expressing HEK cells. Scale bars: 50 µm. Images shown in E and F are taken from the overviews displayed in Figs S11 and S12.

DYNLL1 is described to be localized first to the nucleus of elongating spermatids then later in the cytoplasm and residual bodies (Fig. 7A) (Wang et al., 2005). Western blots on protein extractions from *Actl7b^−/−^*, *Actl7b^+/−^* and *Actl7b^+/+^* testis showed that DYNLL1 and DYNLL2 protein amounts are not reduced in *Actl7b^−/−^* testis compared with *Actl7b^+/−^* and *Actl7b^+/+^* testes (Fig. 7B, Fig. S10). Of note, ACTL7B levels were reduced in Actl7b^+/−^ testes. Immunofluorescent staining against DYNLL1 and DYNLL2 revealed that both proteins show the identical localization. As described, they localize to the head of early elongating spermatids and were present in the cytoplasm at later developmental stages (Figs S11 and S12). Interestingly, DYNLL1 and DYNLL2 expression correlates with the onset of defects observed in spermatids of *Actl7b^−/−^* mice. In *Actl7b^−/−^* testis, the first clear differences in DYNLL1 and DYNLL2 staining compared with wild type were seen at around stage I-III of the seminiferous cycle. Both light chains should be homogeneously present throughout the whole cytoplasm, which should be orientated towards the lumen in a stream-like fashion. In *Actl7b^−/−^* testes, however, the DYNLL1/2-positive cytoplasm is arranged in almost round sacs (Fig. 7C,D, Figs S11 and S12). Vacuolation of the staining and foci of concentrated DYNLL1/2 were detected. Taken together, these results suggest that the localization of DYNLL1 and DYNLL2 is altered in the absence of ACTL7B, whereas the amount of protein is unchanged. In HEK*^Actl7b-eGFP^*cells, ACTL7B localized mainly to the cytoplasm, and DYNLL1 and DYNLL2 were localized throughout the whole cytoplasm and the staining appeared fibrous (Fig. 7E,F, Fig. S13). In comparison, DYNLL1 and DYNLL2 localized mainly in the nucleus in wild-type HEK cells and only weakly in the cytoplasm. Furthermore, the staining appeared much more even in wild-type HEK cells. Next, we transfected HEK cells with an expression plasmid containing the murine DYNLL1 or DYNLL2 fused to mCherry. Here, a similar staining pattern can be seen (Fig. S14). This supports the notion that ACTL7B interacts with and controls DYNLL1 and DYNLL2 localization in the cell.

Of note, F-/G-actin ratios are not significantly different in *Actl7b^−/−^* compared with *Actl7b^+/+^* or *Actl7b^+/−^* testis, suggesting that actin filament turnover is unlikely to be affected in the absence of ACTL7B (Fig. S15A). Even though the co-immunoprecipitation did not reveal ACTL7B-actin interaction, actin filament arrangement is disturbed in some areas of *Actl7b*^−/−^ seminiferous tubules (Fig. S15B).

### Loss of ACTL7B leads to proteomic changes in *Actl7b^−/−^* testis

**Fig. 8.**
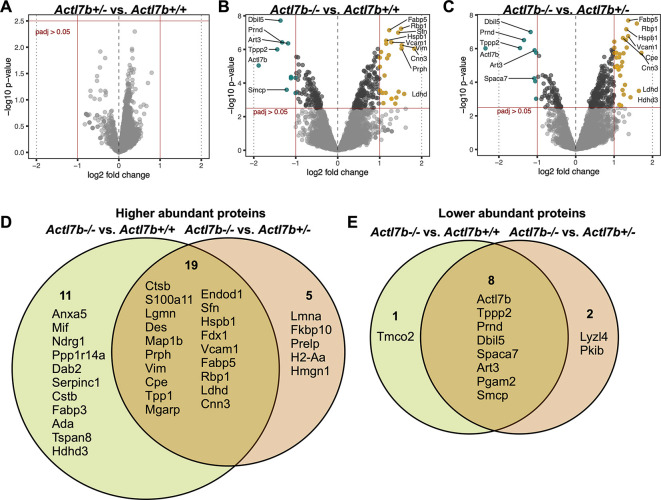
**Proteomic analysis of *Actl7b*-deficient testis.** (A-C) Volcano plots showing differential abundance (DA) of proteins in *Actl7b^+/−^* compared with *Actl7b^+/+^* (A), in *Actl7b^−/−^* compared with *Actl7b^+/−^* (B) and in *Actl7b^−/−^* compared with *Actl7b^+/+^* (C) testis. Proteins showing a significant DA are indicated in teal (less abundant) and yellow (more abundant) (adjusted *P*-value>0.05). Top DA proteins are labeled with their corresponding gene symbol. (D,E) Venn diagrams showing the overlap of significantly higher (D) and lower (E) abundance proteins in the comparisons of *Actl7b^−/−^* with *Actl7b^+/+^*, and *Actl7b^−/−^* with *Actl7b^+/+^* testis (adjusted *P*-value≤0.05, LFC≤1).

In order to analyze alterations in the testicular proteome in *Actl7b*-deficient mice, protein samples isolated from whole testes of five *Actl7b^−/−^*, five *Actl7b^+/−^* and five *Aclt7b^+/+^* mice were used for mass spectrometric analysis (Table S3). Principal component analysis (PCA) showed that the *Actl7b^−/−^* samples cluster is separated from the *Actl7b^+/−^* and *Actl7b^+/+^* samples clusters (Fig. S16A). Differential abundance (DA) analysis revealed no significant difference in protein abundance in *Actl7b^+/−^* compared with *Actl7b^+/+^* samples (Fig. 8A, Fig. S16B). Differentially abundant proteins were detected in *Actl7b^−/−^* samples compared with *Actl7b^+/−^* and *Actl7b^+/+^* samples, respectively (Fig. 8B,C). Thirty proteins were more abundant and nine proteins were less abundant in *Actl7b^−/−^* compared with *Actl7b^+/+^* samples; 24 proteins were more abundant and 10 proteins were less abundant in *Actl7b^−/−^* compared with *Actl7b^+/−^*, with a stringent log fold change (LFC) of ≥1 (Fig. 8D,E, Fig. S16B). Nineteen of the more abundant proteins were detected in both comparisons (Fig. 8D).

Several Sertoli cell-expressed proteins, such as the type III intermediate filament proteins vimentin, desmin and peripherin (Mruk and Cheng, 2004; Vogl et al., 2008), as well as fatty acid-binding protein 5 (FABP5) and intracellular retinol-binding protein 1 (RBP1) (Oresti et al., 2013; Griswold, 2022), were found to be more abundant in *Actl7b^−/−^* testicular samples. Differential abundance of these mostly Sertoli cell-enriched proteins might indicate a secondary effect caused by arrested and degrading/apoptotic germ cells. Moreover, proteins associated with protein or nucleic acid degradation, as well as early apoptosis, were more abundant in *Actl7b^−/−^* testis (ANXA5, CTSB, LGMN, TPP1 and ENDOD1). Of note, CTSB was also shown to be more abundant via western blot (Figs 8D and 3D). Furthermore, VCAM1, which is proposed to function as an adhesion protein in immunoregulation of the testis, was significantly more abundant in *Actl7b^−/−^* testis (Sainio-Pöllänen et al., 1997). As expected, ACTL7B was detected among the significantly less abundant proteins in *Actl7b^−/−^* compared with *Actl7b^+/−^* and *Actl7b^+/+^* samples. Additional spermatocyte- and spermatid-related proteins were detected to be less abundant in *Actl7b^−/−^* samples (ART3, SPACA7, SMCP, TPPP2, TMCO2, PGAM2, LYZL4, PRND and PKIB). One example is the prion-like protein doppel (PRND), which is expressed both in Sertoli cells and in spermatids during the final stages of spermiogenesis (Allais-Bonnet and Pailhoux, 2014). Knockout of *Prnd* leads to male mouse infertility (Behrens et al., 2002; Paisley et al., 2004). Here, immunohistochemical staining against PRND on testis sections revealed that PRND is absent in some areas of *Actl7b^−/−^* seminiferous tubules (Fig. S17).

When applying a less stringent LFC of ≥0.5, 193 proteins were more abundant and 59 proteins less abundant in *Actl7b^−/−^* compared with *Actl7b^+/+^* samples (Fig. S16B, Table S1). These proteins were used to analyze the enrichment in biological processes (Fig. S18A,B). Proteins that were more abundant in *Actl7b^−/−^* compared with *Actl7b^+/+^* showed an enrichment in protein degradation processes, apoptosis, oxidative stress response, cell motility and localization (Fig. S18A). Levels of the autophagy-related protein LC3B, which were shown to be increased in *Actl7b^−/−^* testis compared with *Actl7b^+/+^* testis via western blot (Fig. 3D), were also identified as more abundant by mass spectrometry (*Map1lc3b*; Table S1). Interestingly, N-cadherin (encoded by *Cdh2*) was more abundant in *Actl7b^−/−^* samples. In Sertoli cells, N-cadherin is required for blood-testis-barrier integrity (Jiang et al., 2015). Sertoli cell N-cadherin interacts with actin and vimentin, and is found at the ectoplasmic specializations between Sertoli cells and germ cells, and in the basal compartment of the seminiferous tubules (Mruk and Cheng, 2004). Ezrin, which was also more abundant in *Actl7b^−/−^* testis, regulates Sertoli cell-spermatid-adhesion, influences spermatid polarity and is involved in residual body/phagosome transport (Gungor-Ordueri et al., 2014). It has been shown that in cells transfected with a mutated ezrin, the structures of tubulin, actin and vimentin are altered (Zhang et al., 2020). Furthermore, it has been shown that ezrin interacts with VCAM1 *in vitro* (Barreiro et al., 2002). Immunohistochemical staining against ezrin showed staining surrounding the seminiferous tubules and an intense accumulation of ezrin around germ cells and vacuolations in *Actl7b^−/−^* seminiferous tubules, indicating increased germ cell transport and clearance (Fig. S18C). In comparison, ezrin was below the detection threshold for immunohistochemistry in the epithelial tissue in *Actl7b^+/−^* and *Actl7b^+/+^* samples, where ezrin was detected only surrounding the seminiferous tubules. On the other hand, less abundant proteins showed an enrichment in male gamete formation, fertilization and reproduction, as well as in microtubule-based movement (Fig. S18B).

### *ACTL7A* and *ACTL7B* are highly conserved across primates and rodents

As *ACTL7A* and *ACTL7B* are both testis specific and show sequence similarity, we performed evolutionary analysis of both genes to compare their levels of sequence conservation and predict their essentiality. *ACTL7A* and *ACTL7B* show 57% amino acid and 52% coding sequence identity in *Mus musculus*. Of note, human and mouse ACTL7B show 85.9% pairwise amino acid identity. Selective pressures on the codon level were assessed via the nonsynonymous/synonymous substitution rate ratio (ω=dN/dS). This ratio distinguishes between purifying selection (codon sequence conservation) (ω<1), neutral evolution (ω=1) and positive selection (ω>1). Analysis of the selective constrains on *ACTL7A* and *ACTL7B* revealed that both are under strong purifying selection across primates and rodents (Table 1, Figs S19 and S20). The evolutionary rates (ω) of the whole sequences across all included species trees were significantly lower than 1 (*ACTL7A*: ω=0.17, *P*>0.001; *ACTL7B*: ω=0.06, *P*>0.001), with *ACTL7B* showing an even lower evolutionary rate compared with *ACTL7A*. When comparing selective pressures between primates and rodents, we found no significant difference in evolutionary rate between the clades. Hence, *ACTL7A* and *ACTL7B* are equally highly conserved in both clades. Finally, the selective constraints acting on each codon site were calculated across the whole tree. Ninety-seven percent of the *ACTL7B* codon sites were conserved, further confirming that *ACTL7B* is under strong purifying selection. Similarly, 83% of the codon sites were conserved for *ACTL7A*. These results indicate that changes in the coding sequences, such as mutations, are highly detrimental and are strongly selected against. This usually indicates that the gene and its protein product are highly essential. *ACTL7B* seems to be more strongly conserved than *ACTL7A*. Additionally, as both rodents and primate clades, including the murine and human sequences, are equally conserved, we can surmise that *ACTL7B* is highly essential for both human and mouse spermatogenesis.

**Table 1.**
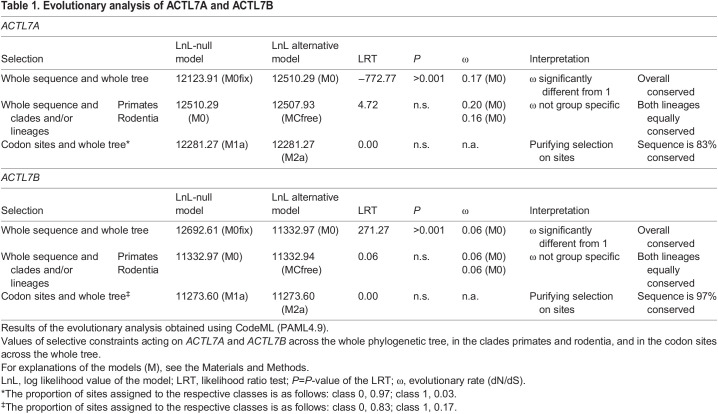
Evolutionary analysis of ACTL7A and ACTL7B

## DISCUSSION

Here, we have generated an *Actl7b*-deficient mouse model to analyze the role of ACTL7B in spermatogenesis. We show that, in line with published data (Clement et al., 2023), loss of ACTL7B led to male infertility in mice due to the absence of functional mature sperm. *Actl7b^−/−^* spermatids showed an arrest in development, resulting in a wide variety of abnormalities starting after step 9, such as detached acrosomes and membranes, malformed flagella, and impaired eviction of excess cytoplasm. The majority of spermatids were subsequently degraded. A subset of degrading and immature spermatids was released into the lumen of the seminiferous tubules. Vesicles containing degrading spermatids seem to be eliminated by Sertoli cells and levels of autophagy marker proteins are increased in *Actl7b^−/−^* testes. *Actl7b^+/−^* males showed reduced ACTL7B levels but remained fertile, producing motile viable sperm at concentrations similar to wild type. As the genes of human and mouse *ACTL7B* are highly similar, we can surmise that ACTL7B variants can lead to failed spermatogenesis and infertility in humans.

Interestingly, the actin-related protein ACTL7B does not seem to interact with the actin cytoskeleton, as we could not find a change in steady-state actin filaments. Rather, ACTL7B seems to link to the microtubule network related functions.

The phenotype of *Actl7b* deficiency described here is highly similar to the one described by Clement et al., who used homologous recombination in embryonic stem cells to generate and analyze an *Actlt7b*-KO mouse (Clement et al., 2023). Both models demonstrate severe reductions in epididymal sperm number, germ cell loss in testis, immature germ cell release from testis and multifaceted morphological sperm abnormalities. Of note, although we observed a 1000-fold reduction in sperm count (approximately 32,000,000 in wild type versus 32,000 in KO), Clement et al. reported this reduction to be only 10-fold. Both models were generated by deleting the entire Actl7b-coding sequence (CDS), but the lines were established on C57Bl/6J by us and on C57BL/6N by Clement et al. (2023). This might explain the phenotypic differences (Simon et al., 2013).

By mass spectrometry analyses, we demonstrated that ACTL7B interacts with dynein light chains DYNLL1 and DYNLL2. Of note, ACTL7B is upregulated in round spermatids whereas dynein light chains DYNLL1 and DYNLL2 are detected later, in step 9 spermatids (Wang et al., 2005). This correlates with the onset of the defects in spermatogenesis of *Actl7b^−/−^* males, suggesting that the ACTL7B-DYNLL1/2 interaction is crucial and leads to the defects observed. The LC8 family light chains DYNLL1 and its paralog DYNLL2 are highly conserved orthologs among mammals (Rapali et al., 2011). LC8 light chains are presumably involved in dynein complex assembly, thereby indirectly affecting cargo binding. The LC8 light chains are hub proteins that form homodimers with high conformational dynamics of binding grooves, interacting with a wide variety of proteins and functioning in multiple cellular processes such as mitosis, intracellular transport, the stabilization of microtubules, nuclear transport, apoptosis, postsynaptic density and regulation of transcription (Rapali et al., 2011; Nyarko et al., 2011; Jespersen and Barbar, 2020; Hall et al., 2008). They bind intrinsically disordered proteins as dimers, thereby linking subunits in multiprotein complexes (Reardon et al., 2020; Asthana et al., 2012). DYNLL1 was further shown to facilitate dissociation of dynactin from dynein, regulating cargo release (Jin et al., 2014). We hypothesize that DYNLL1 and DYNLL2 promote ACTL7B dimerization and stabilization in multiprotein complexes. Alternatively, ACTL7B might be involved in LC8 transport or activation, as absence of ACTL7B caused mislocalization of the LC8 light chains. Furthermore, we show that expression of *Actl7b* in HEK cells alters DYNLL1 and DYNLL2 localization in the cell. Effects of the LC8 light chains on the microtubule structure or the dynein 1 motor complex might explain the phenotype seen in *Actl7b*-deficient mice.

It has been shown that knockdown of the cytoplasmic dynein 1 heavy chain DYNC1H1, causes a disruption of the microtubule structure and polymerization in Sertoli cells in rat (Wen et al., 2018). Furthermore, F-actin organization was perturbed, spermatid polarity was affected, and spermatid transport and release were defective. Finally, phagosome transport was affected. These effects are similar to those described for *Actl7b*-deficient mice here. Another study identified SPEF2 as interaction partner of DYNC1H1 (Lehti et al., 2017). Germ cell-specific knockout of *Spef2* let to multiple spermatid differentiation defects, severely reduced sperm numbers and male mice infertility. It has been proposed that SPEF2 functions as a linker protein, interacting with dynein 1 to facilitate cargo transport along microtubules during spermatid differentiation. ACTL7B might have similar functions. Other dynein light chains have been described in spermatogenesis. For the dynein light chain Tctex-type 4 (DNLT4), 40 different interactors have been identified in human testis, showing the functional variety of dynein light chain interacting proteins (Freitas et al., 2014). In human, absence or lower levels of dynein light chain Tctex-type 1 (DNLT1) have been associated with male infertility (Indu et al., 2015). A detailed analysis of LC8 function in murine spermatogenesis is mandatory in order to reveal the role and consequence of ACTL7B-LC8 interactions.

Mass spectrometric analysis of testicular proteins revealed that Sertoli cell-associated proteins are more abundant in *Actl7b^−/−^* testis compared with *Actl7b^+/−^* and *Actl7b^+/+^* testis, indicating a reaction to defective spermatids. We show that key autophagy marker proteins show higher levels in *Actl7b^−/−^* testes. As TUNEL staining was negative, we favor autophagy as the main cause of spermatid degradation. Even so, apoptosis-related proteins were also found to be more abundant in *Actl7b^−/−^* testis. Intermediate filaments (vimentin, desmin and peripherin), ezrin, N-cadherin and VCAM-1 were found to be more abundant. Intermediate filaments usually surround the Sertoli cell nucleus and from there extend to desmosome junctions, which are localized between adjacent Sertoli cells, and between Sertoli cells and germ cells (Johnson, 2014). Ezrin, which accumulated around germ cells and vacuolations, indicating recent germ cell loss in *Actl7b^−/−^* testis tissue. Ezrin regulates Sertoli cell-spermatid adhesion as well as phagosome transport (Gungor-Ordueri et al., 2014). N-cadherin localizes to ectoplasmic specializations between Sertoli cells and germ cells (Jiang et al., 2015). It interacts with intermediate filaments and actin. The vascular adhesion molecule VCAM1 interacts with ezrin *in vitro* (Barreiro et al., 2002). Under inflammation or chronic conditions, VCAM1 expression can be activated by multiple stimuli including pro-inflammatory cytokines and ROS (Kong et al., 2018). VCAM1 expression has been shown to be increased in Sertoli cells that have been exposed to inflammatory mediators *in vitro* (Riccioli et al., 1995). Taken together, these secondary effects of *Actl7b* deficiency suggest rapid degradation of abnormal, developmentally blocked spermatids by Sertoli cells. Spermatid degradation correlated with a lower abundance of proteins related to spermatids, male gamete formation and fertility.

*ACTL7A* and *ACTL7B* show a high level of sequence identity, and are expressed specifically in the testes of mouse and human, which might suggest functional redundancy (Chadwick et al., 1999; Hisano et al., 2003; Tanaka et al., 2003). However, we have shown that both genes are evolutionarily conserved and under purifying selection in both rodents and primates, suggesting that both genes are required for proper sperm development and function. Furthermore, in murine sperm, ACTL7A is localized to the acrosome and tail, whereas ACTL7B is present in the cytoplasm of round and elongating spermatids (Fu et al., 2012; Tanaka et al., 2003). Although ACTL7B is evicted and detected in residual bodies, ACTL7A is present in the acrosome of mature sperm. Together, the data strongly suggest that ACTL7A and ACTL7B have adapted to different functions. Consequently, the phenotype described for *Actl7a* mutant mice and humans carrying *ACTL7A* variants differ from those described here. Homozygous *ACTL7A* missense mutation causes sperm acrosomal defects and infertility in human and mouse (Xin et al., 2020). *ACTL7A*-deficient sperm showed reduced levels of PLCζ, a sperm-borne oocyte-activation factor, and artificial oocyte activation overcame the infertility caused by *ACTL7A* deficiency. Similar phenotypes have recently been described for human and murine sperm carrying homozygous pathogenic variants in *ACTL9* (Dai et al., 2021). Indeed, ACTL9 seems to interact with ACTL7A, and both proteins are mislocalized when ACTL9 is mutated. Another recent study identified a homozygous missense variant of *ACTL7A* in a teratozoospermic patient (Dai et al., 2022). Analysis of a mouse model carrying an equivalent mutation showed that the acrosome and acroplaxome become detached during spermiogenesis. The acroplaxome, ACTL7A and PLCζ are shed and evicted in cytoplasmic droplets. Supporting the results of previous studies, *Actl7a*-mutated sperm failed to activate the oocyte, leading to infertility. All these studies showed that spermatogenesis is not disrupted when ACTL7A is missing, but acrosome formation is impaired. Hence, the phenotypes of *Actl7b* and *Actl7a* deficiency differ greatly. Here, we additionally showed that ACTL7A levels are not elevated in *Actl7b^−/−^* mice, further arguing against a compensatory role for ACTL7A in *Actl7b^−/−^* mice.

In human, one study identified *ACTL7B* levels to be significantly lower in teratozoospermic individuals compared with unaffected controls (Ahn et al., 2017). Furthermore, single nucleotide polymorphisms in *ACTL7B* have been identified in cohorts of infertile individuals (Tanaka et al., 2007, 2019). However, these have not been directly correlated to the infertility. Finally, *ACTL7B* has been identified to convey a high discriminating power between obstructive and non-obstructive azoospermia subtypes, both at protein and transcript levels, suggesting *ACTL7B* as a screening marker (Davalieva et al., 2022). Our study clearly shows that mutations in *ACTL7B* might be directly connected to male infertility, calling for further investigations in human.

## MATERIALS AND METHODS

### Ethics statement

Animal experiments were performed according to the German law of animal protection and in agreement with the approval of the local institutional animal care committees Landesamt für Natur, Umwelt und Verbraucherschutz, North Rhine-Westphalia (AZ81-0204.2018.A369).

### Generation of *Actl7b*-deficient mice (*Mus musculus*)

Single guide RNAs (sg1_ts, 5′-CACCCGGACACGGCGTGTCGCAT; sg1_bs, 5′-AAACCATGCGACACGCCGTGTCC; sg2_ts, 5′-CACCAATACGGAAGATCAAGGCG; sg2_bs, 5′-AAACGCGCCTTGATCTTCCGTAT) were designed using the Benchling CRISPR Guide RNA design tool (https://www.benchling.com/crispr/; ENSMUSG00000070980) and tested in embryonic stem cells as described previously (Schneider et al., 2016). The selected guides were ordered as crRNA sequences (Integrated DNA Technologies) and prepared for electroporation as described previously (Arévalo et al., 2022). Briefly, crRNAs were annealed to tracRNA (IDT) (50 nM) and mixed with Cas9 (IDT) in OPTI-MEM (Thermo Fisher Scientific). Potential off-targets of the single guide RNAs were analyzed using the ‘CRISPR-Cas9 guide RNA design checker’ from IDT (https://eu.idtdna.com/site/order/designtool/index/CRISPR_SEQUENCE). The guides had off-target scores of 91/100 and 86/100, respectively. Only two potential off-targets on chromosome 4 were detected (for guide 2), both of which are localized in non-coding areas.

CRISPR-Cas9-mediated gene editing of oocytes was performed as described previously (Arévalo et al., 2022). Six- to eight-week-old B6D2F1 females were superovulated by intraperitoneal injections of 5 i.u. pregnant mare serum (PMS) and 5 i.u. human chorionic gonadotropin (hCG) and mated with B6D2F1 males. Oocytes were isolated at 0.5 dpc and electroporated in OPTI-MEM containing the guide RNA mix using a BioRad Gene Pulser . After recovery and washing, the oocytes were incubated in KSOM (Merck) overlaid with mineral oil at 37°C overnight.

Developing two-cell stage embryos were transferred into the fallopian tube of pseudo-pregnant CD1 foster mice. Offspring was genotyped by PCR and sequenced to identify founder animals. Selected founders were backcrossed to C57BL/6J mice and the F1 generation was sequenced. The *Actl7bΔ* allele (NM_025271.2:c.159_631del) was further back-crossed to C57BL/6J mice. Starting from the N2 generation, analyses were performed. The allele was registered with Mouse Genome Informatics (Actl7b^em1Hsc^; 671828).

No significant differences in body weight were monitored for genetically altered mice compared with wild-type mice. Mice were assessed as newborn litter, as litter at weaning and as individual animals every 3 months starting at 8 weeks of age. Nutritional status, posture, coat and orifices, as well as behavior and reaction to handling, were normal in *Actl7b*-deficient mice.

### Genotyping and sequencing of mice

Primers flanking the gene edited region (Actl7b_fwd, 5′-GGGACACAGGTTCCACTCAAC; Actl7b_rev, 5′-AGGTAGTTGGTGAGGTCGCA) were used to amplify both the wild-type and edited allele (cycling conditions: 5 min at 95°C; 35 cycles of 30 s at 95°C, 30 s at 60°C and 45 s at 72°C; 5 min at 72°C). PCR products (wild-type allele, 607 bp; *Actl7b^−/−^*, 134 bp) were separated on agarose gels. Samples for sequencing were prepared as described previously (Merges et al., 2022) and sent to GATC/Eurofins for sequencing.

### Fertility assessment

Male mice, aged between 8 and 12 weeks, were mated 1:1/1:2 with C57BL/6J females, and females were examined for the presence of a vaginal plug daily. Plug-positive females were separated and monitored for pregnancies and litter sizes. A minimum of five plugs per male were monitored. Pregnancy rate was determined by calculating the percentage of plugs resulting in live-born litter.

### Immunohistochemistry and immunofluorescence

Tissues were fixed in Bouin's solution (4°C, overnight), embedded in paraffin wax and 3 µm sections were generated using a microtome (Microm CP60). Heat-mediated antigen retrieval was performed at pH 6 or pH 9 [pH 6: rabbit monoclonal (clone SD08-04) anti-DYNLL1 (Invitrogen, SD08-04, 1:1500), rabbit polyclonal anti-DYNLL2 (Proteintech, 16811-1-AP, 1:1500), rabbit polyclonal anti-ACTL7B (Proteintech, 13537-1-AP, 1:750), mouse anti-PRM2 (Briar Patch Biosciences, Hup2B, 1:200), rabbit polyclonal anti-ODF2 (Proteintech, 12058-1-AP, 1:500), monoclonal (clone 3C12) mouse anti-Erzin (Santa Cruz, sc-58758, 1:100), rabbit polyclonal anti-TNP1 (Abcam, ab73135, 1:1000) and rabbit anti-cP2 (custom antibody, Davids Biotechnologie, 1:500 (Arévalo et al., 2022); pH 9: rabbit anti-actin monoclonal (clone EPR16769) (Abcam, ab179467, 1:500) and rabbit polyclonal anti-PRND (Proteintech, 26947-1-AP, 1:500)]. For slides stained using anti-ACTIN and anti-PRND antibodies, an additional peroxidase blocking step was performed. Slides stained against PRM2, cP2 and TNP1 were additionally treated with decondensation buffer, as described previously (Schneider et al., 2020).

Sections stained using anti-DYNLL1, anti-DYNLL2, anti-TNP1, anti-cP2, anti-PRND, anti-ODF2 and anti-actin were processed using the VectaFluor Horse Anti-Rabbit IgG, DyLight 488 Antibody Kit (Vector Laboratories; DI-1788). Sections stained for ezrin were processed with the VectaFluor Anti-Mouse IgG, DyLight 594 Kit (Vector Laboratories; DI-2794). sections stained for PRM2 were processed with the VectaFluor Duet Immunofluorescence Double Labeling Kit, DyLight 594 Anti-Rabbit, DyLight 488 Anti-Mouse (Vector Laboratories; DK-8828). Sections stained for anti-ACTL7B were processed using the Vectastain ABC-AP Kit (Vector Laboratories; AK-5001) and ImmPACT Vector Red alkaline phosphatase substrate (Vector Laboratories; SK-5105). For all stainings, an extra 30 min blocking step with 5% BSA in PBS was performed. Fluorescent stainings were DAPI counterstained with ProLong Gold antifade reagent (Thermo Fisher Scientific) or ROTI Mount FluorCare DAPI (Carl Roth).

Peanut agglutinin (PNA)-fluorescein isothiocyanite (FITC) Alexa 615 Fluor 488 conjugate (Invitrogen Molecular Probes) was used on deparaffinized sections that were fixed using Bouin's solution. After permeabilization with 0.1% Triton-X 100 for 5 min at room temperature, slides were blocked for 1 h with 1% BSA and incubated for 1 h with PNA (1:200). Slides were mounted with or ROTI Mount FluorCare DAPI (Carl Roth).

TUNEL assay was performed using the TUNEL Assay Kit- HRP-DAB (Abcam, ab206386) according to manufacturer's instructions. Positive control slides were generated by treatment with 1 µg/µl DNase I in TBS/1 mM MgSO_4_ for 20 min at room temperature, as recommended by the manufacturer.

Testicular sperm were stained using mouse monoclonal (clone DM1A) anti-α-tubulin (Abcam, ab7291, 1:1000) antibodies. After permeabilization with 0.1% Triton-X 100 for 15 min at room temperature, slides were blocked for 1 h with 5% BSA, incubated for 1 h with anti-α-tubulin (1:1000) and mounted with or ROTI Mount FluorCare DAPI (Carl Roth).

PNA-Mito red staining was performed on PFA-fixed (4%, 20 min at room temperature) epididymal sperm. Sperm were incubated with 5 µg/ml Peanut agglutinin (PNA)-fluorescein isothiocyanite (FITC) Alexa 615 Fluor 488 conjugate (Molecular Probes) and 20 nm MitoTracker Red CMXRos (Cell Signaling, 9082) for 45 min at room temperature, washed in PBS and smeared on slides. Slides were mounted with or ROTI Mount FluorCare DAPI (Carl Roth).

Imaging was performed using a confocal Visitron VisiScope and the VisiView Software. Anti-ACTL7B stained sections were counterstained with Haemalum acidic Mayer (Waldeck) and imaged using a Leica DM5500 B microscope. Imaging of testicular and epididymal sperm was performed using a Leica DM5500 B microscope. Staining against DYNLL1 and DYNLL2 was imaged using a LSM 710 (Zeiss).

### Protein extraction for immunoblotting and mass spectrometry analysis

Whole testis tissue was homogenized in 1 ml per 100 mg tissue 1:10 RIPA buffer (Cell Signaling Technology) supplemented with Protease Inhibitor (cOmplete ULTRA Tablets, Mini, EASYpack; Roche, Mannheim, Germany). After incubation on ice for 15 min, the samples were sonicated for 5 min using the Bioruptor UCD-200TM-EX (Tosho Denki). Next, the samples were centrifuged for 30 min at 14,000 rpm (20.817 ***g***) at 4°C and the supernatant was used for downstream applications.

### Immunoblotting

Protein extracts were separated on a 12% SDS gel with a 5% stacking gel and transferred to PVDF membranes using the Trans-Blot Turbo System (BioRad). Membranes were blocked using 5% milk for 1 h at room temperature. Primary antibodies (rabbit polyclonal anti-ACTL7B; Proteintech, 13537-1-AP), mouse monoclonal (clone DM1A) anti-α-tubulin (Santa Cruz Biotechnology, sc-8035), rabbit monoclonal (clone SD08-04) anti-DYNLL1 (Invitrogen, SD08-04), rabbit polyclonal anti-DYNLL2 (Proteintech, 16811-1-AP), rabbit monoclonal (clone EPR21033) anti-CSTB (Abcam, ab214428) and rabbit polyclonal anti-LC3A/B (ab128025) were diluted 1:1000 in 5% milk and membranes were incubated at 4°C overnight. After washing in TBST, the membranes were incubated with secondary antibodies [polyclonal goat anti-rabbit IgG/HRP (P044801-2; 1:2000), polyclonal rabbit anti-mouse IgG/HRP (P026002-2; 1:1000), Agilent Technologies/Dako] for 1 h at room temperature. After washing in TBST, the signals were detected using WESTARNOVA2.0 chemiluminescent substrate (Cyanagen) and the ChemiDoc MP Imaging system (Bio-Rad). For western blots after Co-IP SuperSignal West Femto Maximum Sensitivity Substrate (Thermo Scientific) was used.

### Macroscopic analysis of testis

Sections of Bouin-fixed testis were processed and stained with Hemalum solution acid (Henricks and Mayer, 1965) and Eosin Y solution (Carl Roth) as described previously (Merges et al., 2022).

### Periodic acid-Schiff staining

PAS staining was performed as described previously (Schneider et al., 2020). Deparaffinized, re-hydrated slides were incubated for 10 min in periodic acid (0.5%), washed, incubated for 20 min with Schiff's reagent and counterstained. Elongating spermatids per tubule cross-section were counted.

### Isolation of epididymal and testicular sperm

Sperm from wild-type and *Actl7b^+/−^* males were isolated from the cauda epididymis by swim-out as described previously (Schneider et al., 2016). Epididymal tissue was incised multiple times and incubated in PBS at 37°C for 20-30 min. Sperm count was performed using a Neubauer counting chamber.

Testicular sperm were isolated as described by Kotaja et al. (2004), pipetted onto slides, coverslipped and snap frozen in liquid nitrogen. The cover slip was flipped off and slides were fixed for 5 min in 90% ethanol.

### Testicular daily sperm production

Daily sperm production was determined as described by Juma et al. (2017), with modifications. In brief, after removal of the tunica albuginea, testes were homogenized in 400 µl DSP buffer (0.15 M NaCl, 0.1 M NaN_3_ and 0.05% Triton-X 100 in water). Wild-type and heterozygous samples were adjusted to 4 ml, and KO samples were adjusted to 2 ml final volume using DSP buffer. Elongating spermatids were counted using a Neubauer counting chamber; the result was divided by 4.84.

### Transmission electron microscopy

Testis tissue was prepared as described previously (Merges et al., 2022). In brief, the tissue was fixed in 3% glutaraldehyde at 4°C overnight, washed, post-fixed with 2% osmium tetroxide at 4°C for 2 h and washed again. After dehydration and contrasting in 70% (v/v) ethanol 0.5% (m/v) uranyl acetate (1–1.5 h, 4°C), samples were washed with propylenoxide (three times for 10 min at room temperature) and stored in propylenoxide:Epon C (1:1 v/v) at 4°C overnight. Next, the pellets were embedded in Epon C (70°C, 48 h) and ultra-thin sections were prepared. Ultrathin sections were contrasted with UranyLess (Electron Microscopy Sciences) and lead citrate. Images were taken using the Philips CM10 transmission electron microscope equipped with analySiS imaging software and a Zeiss Crossbeam 550 FIB scanning electron microscope equipped with a retractable STEM detector.

### Eosin-Nigrosin staining

Staining was performed as described previously (Merges et al., 2022) using 50 μl of sperm swim-out and 50 μl Eosin-Nigrosin stain [0.67 g Eosin Y (color index 45380), 0.9 g sodium chloride, 10 g Nigrosin (color index 50420) and 100 ml deionized H_2_O]. Two-hundred sperm per animal were analyzed.

### Generation of HEK*^Actl7b-eGFP^*cells

*Actl7b* CDS was amplified using the Q5 high-fidelity thermostable DNA polymerase (NEB) from mouse testis cDNA using the following primers (GFP-Actl7b_fw, ctcgagctcaagcttcgatggcgacaaagaacag; GFP-Actl7b_rv, ggtaccgtcgactgcagttagcacttgctgtagatgg) and inserted into the pEGFP-C1 vector (Clontech) digested with EcoRI (NEB) using the NEBuilder HiFi DNA Assembly kit (NEB) according to the manufacturer's instructions. The pEGFP-C1-Actl7b plasmid was then linearized with ApaLI (NEB) and transfected into HEK293 cells using Lipofectamine 2000 (ThermoFisher Scientific). Next, cells were selected for 3 weeks with 1 mg/ml geneticin (G418 sulfate, Gibco), trypsinized, diluted to 0.5 cells/100 µl and plated 100 µl/well on a 96-well plate. After 6 weeks, five clones with different degrees of fluorescence were selected, expanded in 0.8 mg/ml geneticin and cryopreserved. Protein lysates of the five clones were tested by western blotting using polyclonal anti-ACTL7B (Invitrogen, PA5-113560, 1:1000). All clones showed different expression levels of the 76 kDa fusion protein in agreement with the fluorescence intensity levels. We chose the clone with the highest fluorescence intensity for further experiments.

### Generation of DYNLL1-mCherry and DYNLL2-mCherry expression plasmids

*Dynll1* and *Dynll2* CDC was amplified from mouse testis cDNA using the following primers: Dynll1_fw, AAAAGAATTCATGTGCGACCGGAAGGC; Dynll1_rv, AAAAGAATTCATGTGCGACCGGAAGGC, TTTTGGATCCTTACCAGATTTGAACAGAAGAATG; Dynll2_fw, AAAAGAATTCATGTCTGACCGGAAGGCAG and Dynll2_rv, TTTTGGATCCTTGCCCGACTTGAGAGGAG). The amplified cDNA was cloned into the p-mCherry-N1 plasmid (Clontech, PT3974-5). Plasmids were sent to GATC/Eurofins (Cologne, Germany) for sequencing.

### Transfection of cells

HEK cells were cultured in DMEM (Gibco) with 10% FBS and transfected at 80% confluency. Transfection was performed using 3 μg of expression plasmid with FuGENE HD Transfection Reagent (Promega) according to the manufacturer's instructions. Cells were imaged 12 h later using a Leica DM5500 B microscope.

### Co-immunoprecipitation

Proteins from WT C57BL/6J testis were isolated utilizing the T-PER Tissue Protein Extraction Reagent (Thermo Fisher Scientific) supplemented with Halt Protease Inhibitor Single-Use Cocktail EDTA-Free (Thermo Fisher Scientific) according to the manufacturers instructions. Proteins from HEK cells were isolated using the M-PER Mammalian Protein Extraction Reagent (Thermo Fisher Scientific) supplemented with Halt Protease Inhibitor Single-Use Cocktail EDTA-Free (Thermo Fisher Scientific) according to the manufacturers' instructions.

The ACTL7B antibody (Proteintech; 13537-1-AP) was purified using the Amicon Ultra 30K – 0.5 Centrifugal Filter Device (Merck Millipore, UFC503008) according to the manufacturer's instructions. The purified antibody was coupled to beads using the Dynabeads Antibody Coupling Kit (Thermo Fisher Scientific; 14311D), using 7 µg antibody per mg beads. Next, 7.5 mg of antibody-coupled beads and 5 mg empty beads were used with the Dynabeads Co-Immunoprecipitation Kit (Thermo Fisher Scientific; 14321D) according to the manufacturer's instructions. Proteins eluted from the beads in 1 ml HPH EB (0.5 M NH_4_OH and 0.5 mM EDTA) buffer. 700 µl were lyophilized and sent for mass spectrometry analysis. 300 µl were lyophilized and solubilized in SDS-PAGE sample loading buffer.

DYNLL1 and DYNLL2 antibodies were also purified using Amicon Ultra 30K – 0.5 Centrifugal Filter Device (Merck Millipore). For antibody coupling using the Dynabeads Antibody Coupling Kit (Thermo Fisher Scientific), 10 µg antibody per mg beads were used. 1.5 mg beads were used with the Dynabeads Co-Immunoprecipitation Kit (Thermo Fisher Scientific) for Western Blot applications according to the manufacturer's instructions.

GFP pull-down assay was performed using GFP nanobody-coupled magnetic beads (GFP-Trap Magnetic Particles Kit, Chromotek) according to the manufacturer's instructions. After IP, the bound proteins were eluted in Laemmli's buffer and used for mass spectrometry analysis.

### Mass spectrometry

Proteins from whole testis from five wild-type, *Actl7b*^+/−^ and *Actl7b*^−/−^ mice were isolated as described and used for mass spectrometric analysis. Peptide preparation and liquid chromatography (LC)-mass spectrometry (MS) were performed at the University of Bonn Core Facility Mass Spectrometry.

#### Preparation of co-immunoprecipitation samples for LC/MS

50 µg of protein per sample were subjected to in solution preparation of peptides with the iST 96x sample preparation kit (Preomics) according to manufacturer's recommendations.

#### Whole-cell proteomic analysis: precipitation, proteolysis, peptide labeling and fractionation

Protein lysates with 200 µg protein were mixed with a fourfold volume of chilled acetone (−20°C). After 1 h at −20°C, proteins were sedimented by centrifugation for 15 min at 14,000× ***g***. The supernatant was discarded und pellets air-dried. Pellets were dissolved in 20 µl Lyse buffer (Preomics iST-NHS kit) and protein content was determined by BCA assay. Solutions with 40 µg protein were mixed with 50 µl of the kit's DIGEST solution (3 h, 37°C). 0.25 mg of TMTpro isobaric Mass Tag Labeling reagent (15plex) were added to each sample and incubated at room temperature for 1 h. 10 µl 5% hydroxylamine were used to quench the reaction. The preparation procedure was continued according to the iST-NHS kit instructions. Pooled peptides were dried in a vacuum concentrator, dissolved in 20 mM ammonium formate (pH 10) and fractionated by reverse-phase chromatography at elevated pH with a Reprosil 100 C18 column (3 µm 125×4 mm, Dr Maisch). Sixty fractions were combined into six pools and dried in a vacuum concentrator. Peptides were purified by solid phase extraction (Oasis HLB cartridges, Waters).

#### LC/MS measurements

Before measurement peptides were re-dissolved in 0.1% formic acid (FA) to yield a 1 g/l solution and separated on a Dionex Ultimate 3000 RSLC nano HPLC system (Dionex). 1 µl was injected onto a C18 analytical column (self-packed 400 mm length and 75 µm inner diameter, ReproSil-Pur 120 C18-AQ, 1.9 µm, Dr Maisch). Peptides were separated during a linear gradient from 5% to 35% solvent B (90% acetonitrile and 0.1% FA) at 300 nl/min. The nanoHPLC was coupled online to an Orbitrap Fusion Lumos mass spectrometer (ThermoFisher Scientific).

#### Measurement of peptides from co-immunoprecipitated proteins

Gradient length was 90 min. Peptide ions between 300 and 1600 m/z were scanned in the Orbitrap detector every 3 s with a resolution of 120,000 (maximum fill time 50 ms, AGC target 100%). In a top-speed method, peptides were subjected to higher energy collision induced dissociation (1.0 Da isolation, normalized energy 27%) and fragments analyzed in the Orbitrap (resolution 15,000, AGC target 100% and maximum fill time 22 ms). Fragmented peptide ions were excluded from repeat analysis for 20 s.

#### Targeted measurements

ACTL7B, DYNLL1 and DYNLL2 peptides were analyzed in a targeted mode with 120 min gradient length. MS1 scans were performed every 3 s, peptide precursors were isolated with 1 Da isolation width, fragmented with HCD (28%, maximum injection time 30 ms) and analyzed in Orbitrap.

#### Measurement of TMT-labelled fractions

Gradient length was 150 min. Peptide ions between 330 and 1600 m/z were scanned in the Orbitrap detector with settings as above. In a top-speed method, peptides were subjected to collision-induced dissociation for identification (CID: 0.7 Da isolation and normalized energy 30%) and fragments analyzed in the linear ion trap with AGC target 50% and maximum fill time 35 ms, rapid mode. Fragmented peptide ions were excluded from repeat analysis for 30 s. Top 10 fragment ions were chosen for synchronous precursor selection and fragmented with higher energy CID (HCD: 3 Da MS2 isolation and 65% collision energy) for detection of reporter ions in the Orbitrap analyzer (range 100-180 m/z, resolution 50,000, maximum fill time 86 ms and AGC target 200%).

#### Data analysis

Raw data processing and database search were performed with Proteome Discoverer software 2.5.0.400 (Thermo Fisher Scientific). Peptide identification was carried out with an in-house Mascot server version 2.8.1 (Matrix Science). MS data were searched against *Mus musculus* sequences from the SwissProt database, including isoforms (2022/03, 17132 murine sequences) and contaminants database (cRAP; Mellacheruvu et al., 2013). Precursor ion m/z tolerance was 10 ppm; fragment ion tolerance was 0.5 Da (CID). Tryptic peptides with up to two missed cleavages were searched. C_6_H_11_NO-modification of cysteines (delta mass of 113.08406) and TMTpro on N-termini and lysines were set as static modifications. Oxidation was allowed as a dynamic modification of methionine. Mascot results were evaluated by the Percolator algorithm version 3.02.1 (The et al., 2016) as implemented in Proteome Discoverer. Spectra with identifications above 1% q-value were sent to a second round of database search with semi-tryptic enzyme specificity (one missed cleavage allowed). Protein N-terminal acetylation, methionine oxidation, TMTpro and cysteine alkylation were then set as dynamic modifications. Actual FDR values were 0.7% (peptide spectrum matches) and 1.0% (peptides and proteins). Reporter ion intensities (most confident centroid) were extracted from the MS3 level, with SPS mass match>65%.

Data from targeted measurements were analyzed in Skyline (Pino et al., 2020). Validation of MS2 spectra was aided by a spectral library created on the PROSIT server (Gessulat et al., 2019). Protein quantification was carried out at the MS2 level.

#### Differential abundance analysis

Data for proteins detected in all genotypes and replicates were with more than two peptides were log2 transformed and median normalized. Abundances were analyzed as described previously (Merges et al., 2022). The Bioconductor package proDA (Ahlmann-Eltze and Anders, 2021 preprint) was used [peptide spectrum match (PSM)-level data extracted from Protein Discoverer]. Proteins with LFC>0.5 and LCF>1 (FDR adjusted *P*<0.05) compared with wild type were analyzed. The R-package ggplot2 (Wickham, 2011) was used to plot the data.

### F-actin/G-actin ratio

The F-/G-Actin ratio was analyzed as described previously (Pilo Boyl et al., 2007). Whole-testis tissue was homogenized in 500 µm 1.1×PHEM buffer (600 mM PIPES-Na, 250 mM HEPES-Na, 100 mM EGTA and 20 mM MgCl_2_; pH 6.9) with 1.2% TritonX-100 and incubated on ice for 15 min. Next, the samples were centrifuged in a swing-out rotor for 10 min at 10,000 ***g*** at 4°C. 400 µl of the G-actin congaing supernatant were boiled with 100 µl of 5×SDS loading buffer [110 mM Tris/HCl (pH 6.8), 20% glycerol, 3.8% SDS, 8% β-mercaptoethanol and 0.05% Bromophenol Blue) for 10 min and then cooled on ice. After discarding the rest of supernatant, the pellet was dried and resuspended in 650 µl 1×SDS loading buffer, boiled for 10 min and cooled on ice. Equal volumes of supernatant and pellet fraction were loaded on a 10% acrylamide gel, the gel was blotted on methanol-activated PVDF membrane and anti-β-actin (1:5000; 08-691002; MP Biomedicals) was used to detect the G- and the F-actin fractions. Band quantification was performed using the GE FujiFilm ImageQuant LAS-4000 CH mini Imager (GE Healthcare).

### Evolutionary analysis

Evolutionary rates of *ACTL7B* among rodents and primates were analyzed according to Lüke et al. (2016). *ACTL7B*-coding sequences were obtained from NCBI GenBank and Ensembl genome browser. Phylogenetic trees of considered species were built according to the ‘Tree of Life web project’ (Letunic and Bork, 2021). The webPRANK software was applied for codon-based phylogeny-aware alignment of orthologous gene sequences (Löytynoja and Goldman, 2010). The tree and alignment were visualized using the ETE toolkit (Huerta-Cepas et al., 2016).

Evolutionary rates and selective pressures were determined using codeML implemented in PAML4.9 (Yang, 1997, 2007). The evolutionary rate is based on the calculation of the nonsynonymous/synonymous substitution rate ratio (ω=dN/dS). It distinguishes between purifying selection (ω<1), neutral evolution (ω=1) and positive selection (ω>1).

Different null and alternative models (M) were applied. The M0 model served as basic model for all performed analyses. Different codon frequency settings were tested for the M0 model of each gene and the setting with the highest likelihood was chosen. To test whether alternative models describe the selective constraints within a dataset better than the null models, likelihood ratio tests (LRT) were performed.

In order to determine the overall evolutionary rate and selective pressure on the coding sequence among all included species, we employed two models: M0 ‘one ratio’, in which all branches were constrained to evolve at the same freely estimated evolutionary rate; and M0fix (fixed ratio) in which the evolutionary rate for all branches was constrained to 1. The M0 model calculates the overall evolutionary rate. A LRT between M0 and M0fix was performed to determine whether the calculated evolutionary rate significantly differs from 1 (neutral).

In order to determine whether the selective pressures differ between rodents and primates, we employed two models: M0 ‘one ratio’; MCfree ‘two-ratio’, which allows the estimation of a free and independent ω for the two marked clades. To test whether the alternative MCfree model presents a better fit for the data, we compared the models log likelihood values by LRT.

To test evolution along coding sequences and infer codon sites under positive or purifying selection, we applied LRT comparing the null model M1a ‘nearly neutral’, which does not allow sites with ω>1, with the alternative model M2a ‘selection’, which does. The models assign the codon sites into different classes: class 0, sites under purifying selection (0>ω>1); class 1, sites evolving neutrally (ω=1); class 2a (only M2a), sites subject to positive selection (ω<1). Bayesian statistics were used to identify those codons that have been subject to either positive selection (if the alternative model is the better fit) or purifying selection (if the null model is the better fit). Only sites with posterior probabilities (Bayes Empirical Bayes) higher than 0.95 to be assigned to class 0 or class 2a were determined to be under purifying or positive selection, respectively.

### Statistics

Values are, if not indicated otherwise, given as mean values with standard deviation. Statistical significance was calculated using a two-tailed, unpaired Student's *t*-test. *P*<0.05 was considered significant (*P<0.05, **P<0.005, ***P<0.001).

## Supplementary Material

10.1242/develop.201593_sup1 Supplementary information

Table S1. Mass spectrometry datasets of proteins identified by ACTL7B CoIP using wild-type testes.

Table S2. Dynll2 validated as interaction partner of ACTL7B by targeted analysis of mass spectrometry results from HEK cells expressing ACTL7B-GFP.

Table S3. Mass spectrometry datasets of whole testes lysates from wild-type, Actl7b+/- and Aclt7b-/- male mice.
