# Supplementary material for: Actl7b deficiency leads to mislocalization of LC8 type dynein light chains and disruption of murine spermatogenesis
Source: Development. 2023 Oct 27;150(21):dev201593. doi: 10.1242/dev.201593 (PMC10652042; doi:10.1242/dev.201593)
Supplement: Supplementary information [file develop-150-201593-s1.pdf]

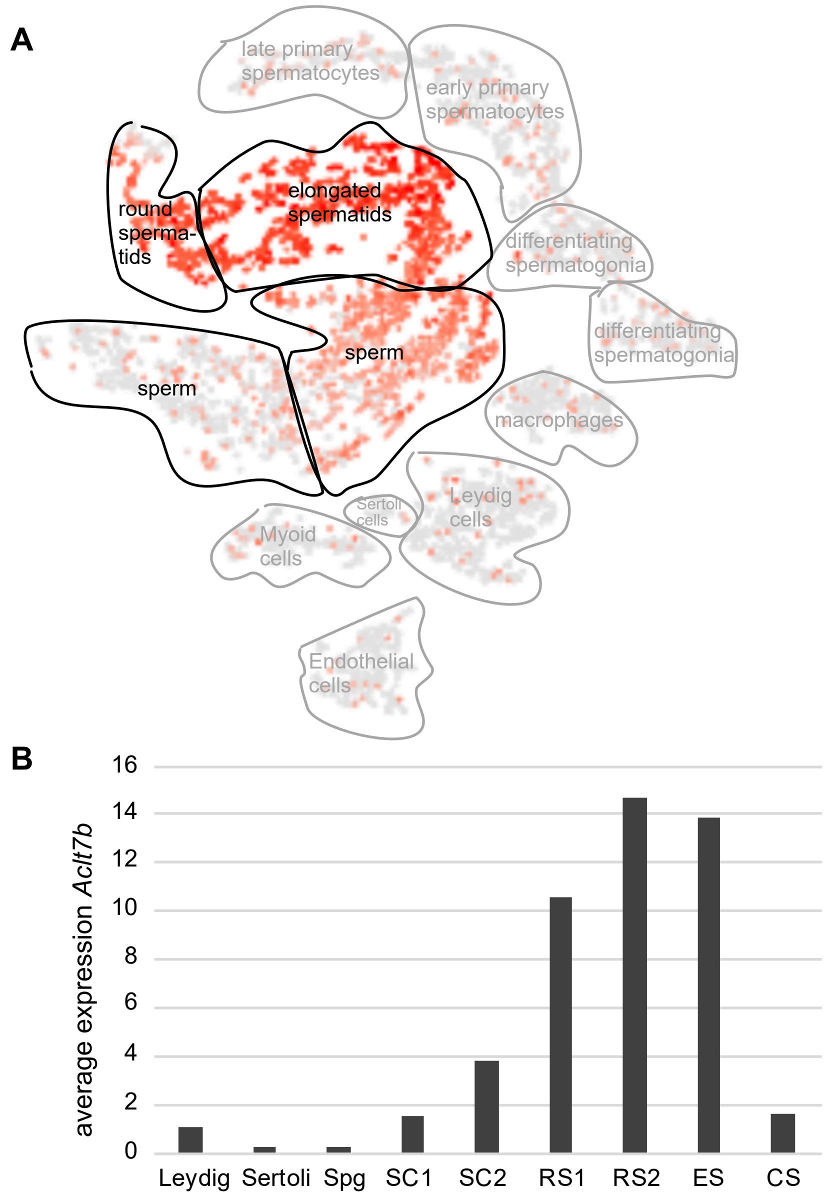

Fig. S1. *Actl7b* expression in young adult human testicular tissue and murine testicular cells. (A) Expression data of *ACTL7B* (indicated in red) in human testis extracted from the Human Testis Atlas adapted from Guo *et al.* 2018. *ACTL7B* is expressed mainly in round and elongated spermatids. **(B)** Average normalized expression of *Actl7b* in testicular cell populations of adult mouse testis adapted from Lukassen *et al.* 2018. *Actl7b* is expressed mainly in round and elongated spermatids. Spg: spermatogonia, SC: spermatocytes, RS: round spermatids, ES: elongating spermatids, CS: condensing spermatids

Development • Supplementary information

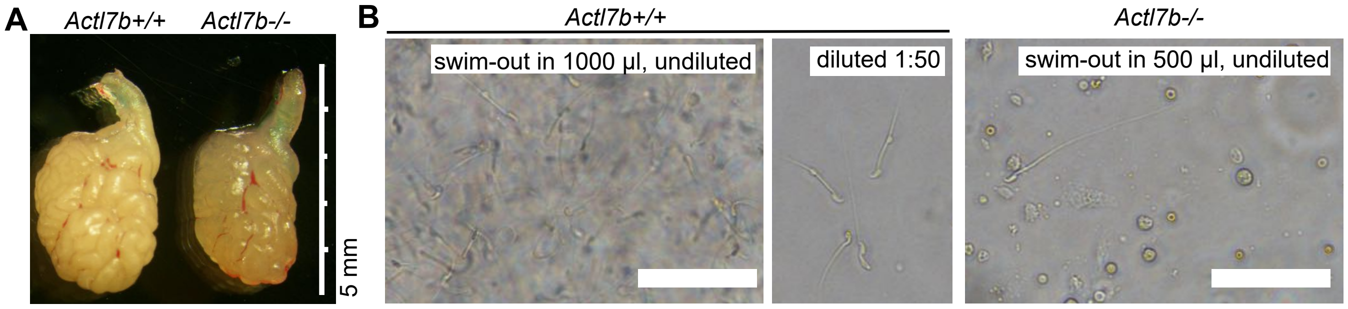

C

Sperm counts from both cauda epididymides

	+/+<br>n = 3	+/-<br>n = 3	-/-<br>n = 3
Individual 1	30500000	34000000	34500
Individual 2	35375000	39750000	41250
Individual 3	28125000	32875000	21000
Average sperm count per genotype	31333333.33	35541666.67	32250.00
s.d.	3696141.01	3687676.55	10310.80

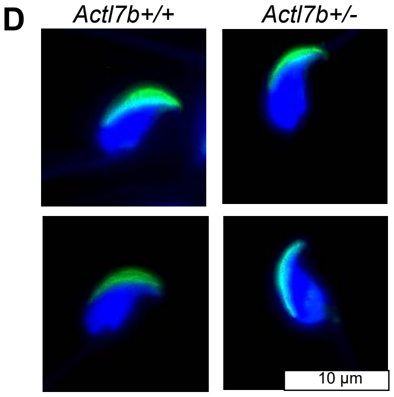

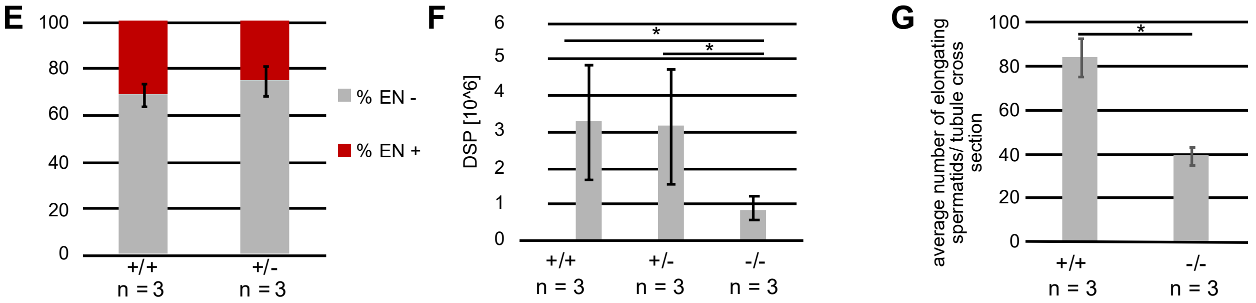

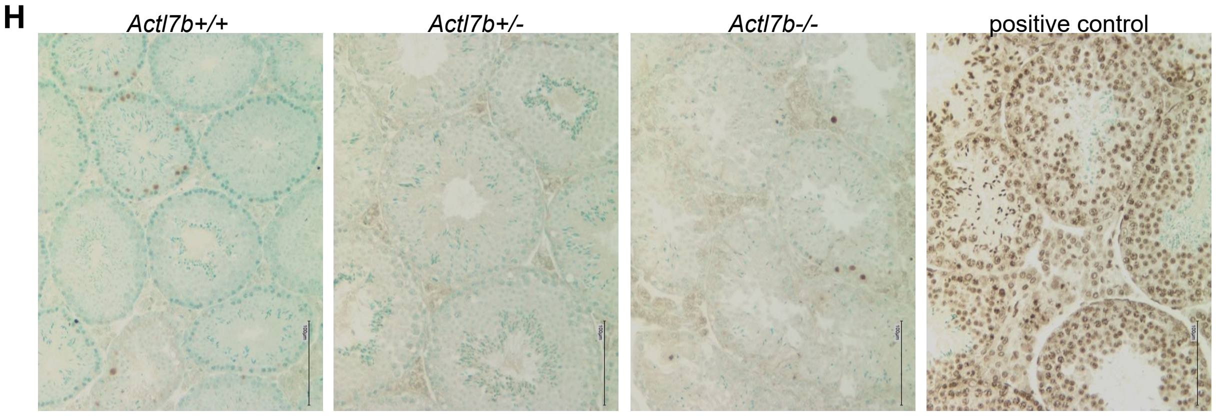

Fig. S2. Sperm production, morphology and viability of *Actl7b*-deficient mice. (A) Representative images of cauda epididymides dissected from an *Actl7b+/+* and *Actl7b-/-* male (littermates). **(B)** Representative images of cell suspension after swim-out from cauda epididymides from *Actl7b+/+* and *Actl7b-/-* mice. Scale: 50 µm **(C)** Sperm count of epididymal sperm from *Actl7b+/+, Actl7b+/-* and *Actl7b-/-* mice. **(D)** Representative images of *Actl7b+/-* and *Actl7b+/+* sperm isolated from cauda epididymis and stained with PNA and DAPI. **(E)** Percentage of Eosin-Nigrosin positive/negative epididymal sperm from *Actl7b+/+* and *Actl7b+/-* mice. **(F)** Average daily sperm production from *Actl7b+/+, Actl7b+/-* and *Actl7b-/-* mice. **(G)** Average number of elongating spermatids counted per PAS-stained seminiferous tubule cross section of *Actl7b+/+, Actl7b+/-* and *Actl7b-/-* mice. **(H)** TUNEL staining on testis sections from *Actl7b+/+, Actl7b+/-* and *Actl7b-/-* mice including positive control (slides treated with DNase I, as recommended by the kits manufacturer). Scale: 100 µm.

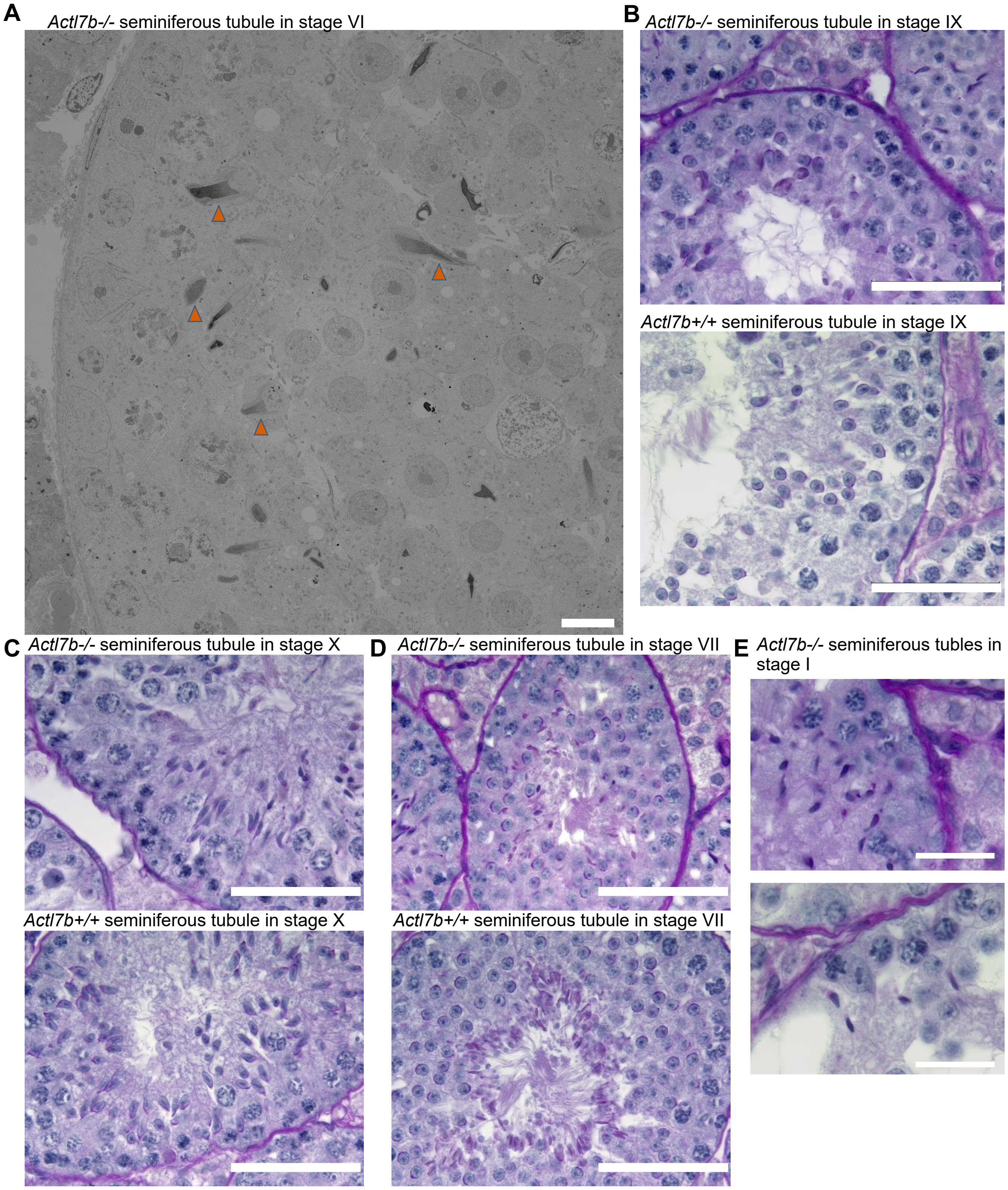

Fig. S3. Spermatogenesis in *Actl7b*-deficient mice. (A) Transmission electron micrograph of *Actl7b-/-* seminiferous tubule. Elongating spermatids, which should be located closer to the lumen and did not condense properly can be seen (vermillion arrow heads). Scale: 5 µm **(B)** PAS staining of testis sections of stage VI seminiferous tubules of *Actl7b+/+* and *Actl7b-/-* mice. **(C)** PAS staining of testis sections of stage X seminiferous tubules of *Actl7b+/+* and *Actl7b-/-* mice. **(D)** PAS staining of testis sections of stage VII seminiferous tubules of *Actl7b+/+* and *Actl7b-/-* mice. **(E)** PAS staining of testis sections of stage I seminiferous tubules of *Actl7b-/-* mice. Scale: 20 µm. Scales B-D: 50 µm**.**

Development • Supplementary information

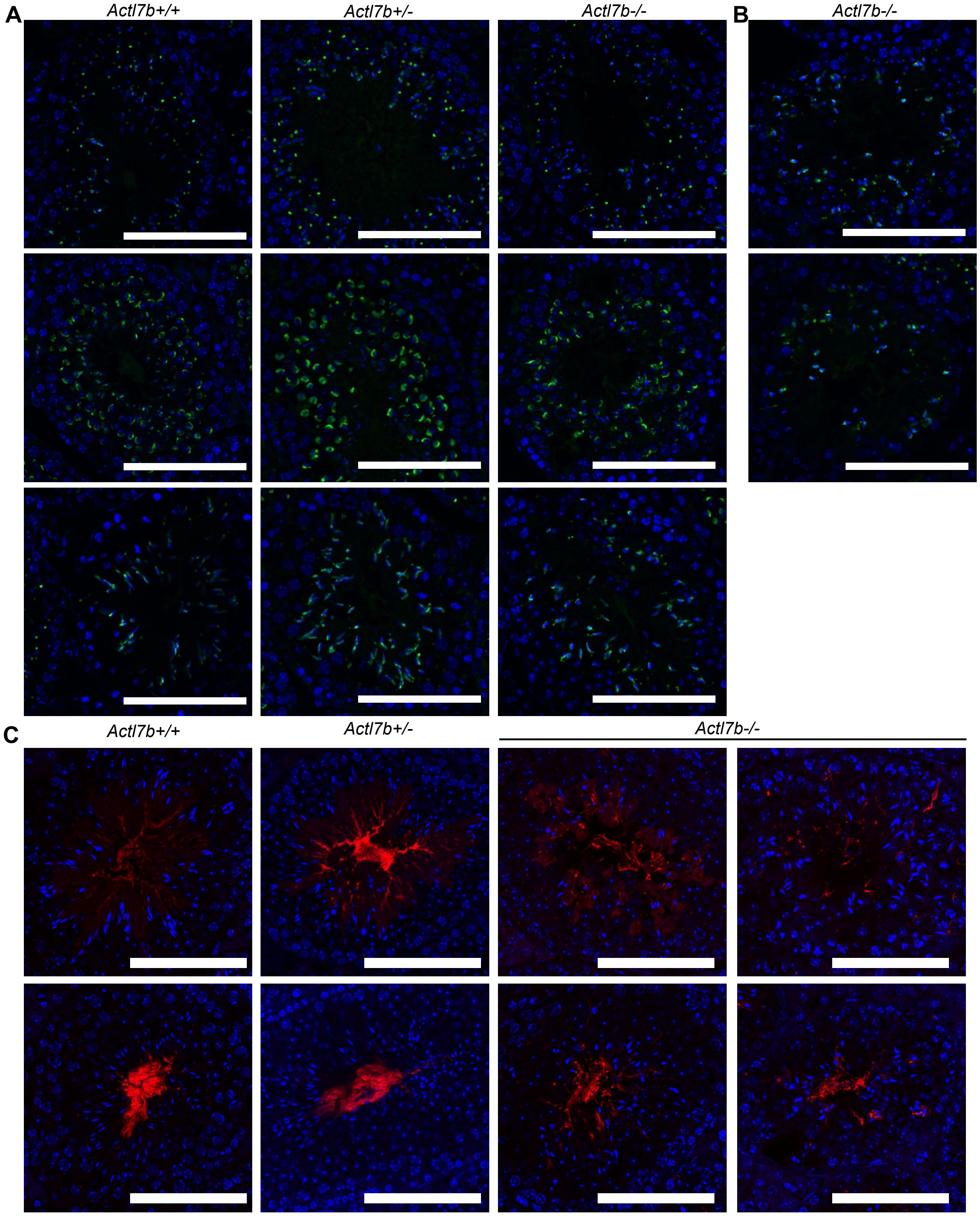

Fig. S4. Basic spermatogenic parameters in *Actl7b*-deficient mice. (A) Testis sections from *Actl7b+/+*, *Actl7b+/-* and *Actl7b-/-* mice stained with PNA and DAPI. **A)** Testis sections from *Actl7b-/-* mice stained with PNA and DAPI. **(A)** Testis sections from *Actl7b+/+*, *Actl7b+/-* and *Actl7b-/-* mice stained against ODF2 and counterstained with DAPI. Scales: 100 µm.

Development • Supplementary information

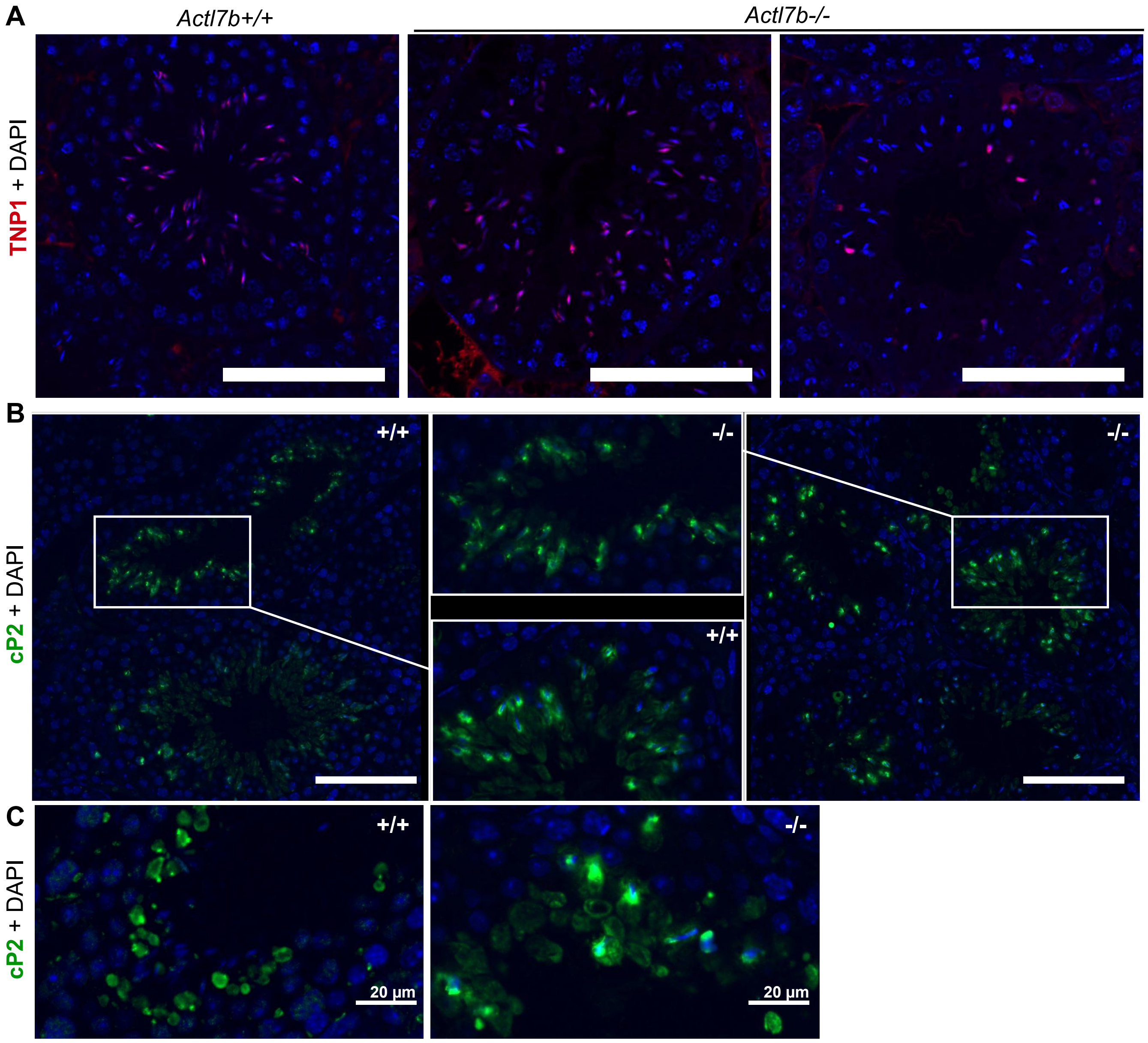

Fig. S5. Nuclear remodeling in *Actl7b*-deficient mice. (A) IHC staining against TNP1 on *Actl7b+/+*, *Actl7b+/-* and *Actl7b-/-* testis sections. **(B)** IHC staining against the cleaved part of PRM2 on *Actl7b+/+*, *Actl7b+/-* and *Actl7b-/-* testis sections. **(C)** IHC staining against the cleaved part of PRM2 on *Actl7b+/+* and *Actl7b-/-* testis sections. Scales A, B: 100 µm.

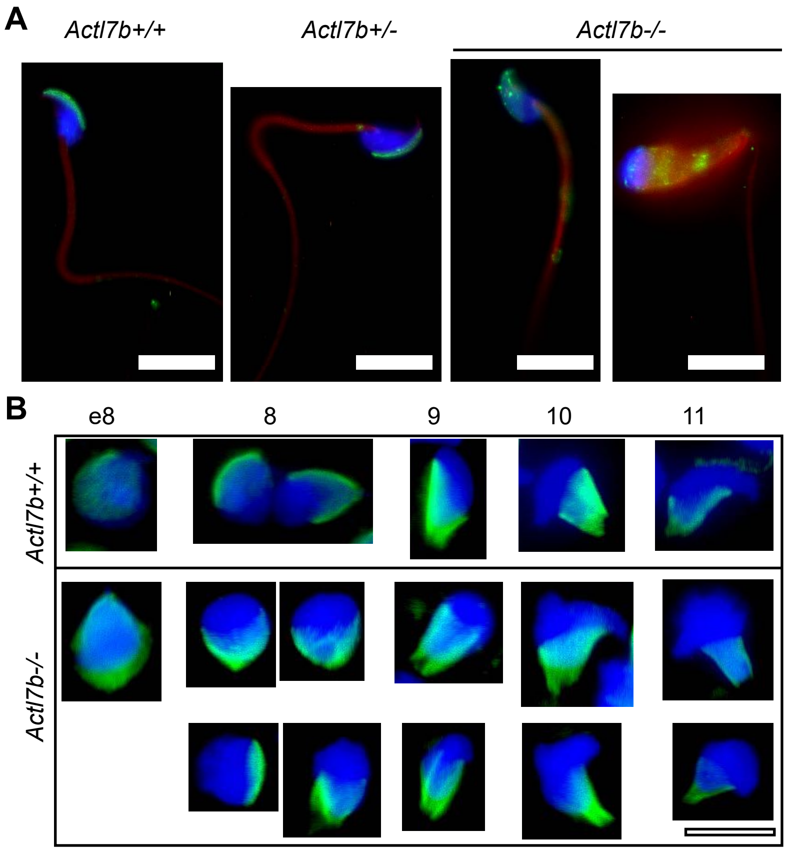

Fig. S6. Flagella and manchette formation in *Actl7b*-deficient mice. (A) Representative images of *Actl7b+/+*, *Actl7b+/-* and *Actl7b-/-* sperm isolated from cauda epididymis and stained with PNA, Mitotracker and DAPI. **(B)** Stainings of the manchette in *Actl7b+/+* and *Actl7b-/-* spermatids isolated from testis. e8 = early step 8 spermatids, 8-11 = step 8-11 spermatids. Scales: 10 µm.

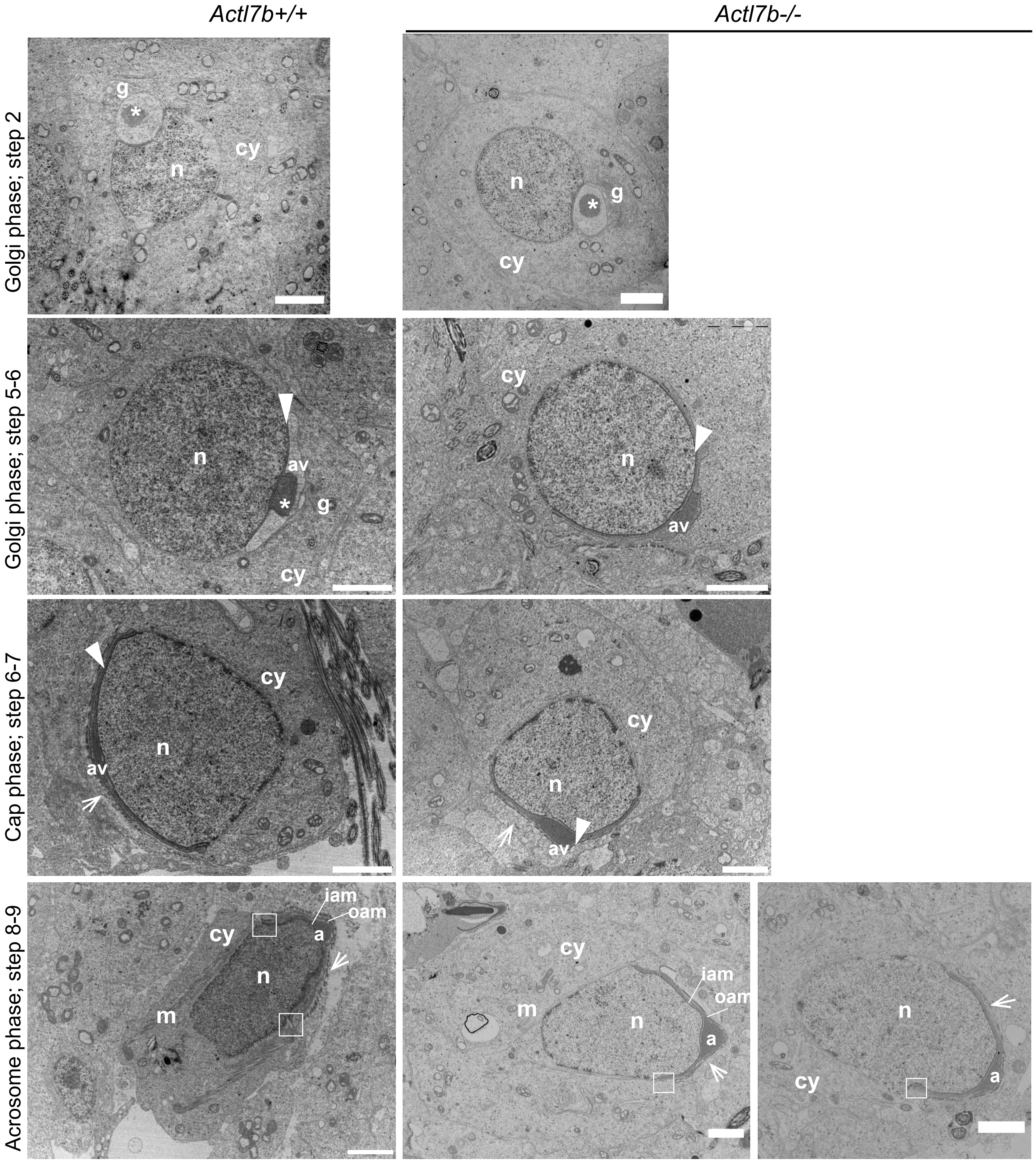

Fig. S7. Transmission electron micrographs of developmental steps 2-9 of spermiogenesis in WT and *Actl7b*-deficient mice. n: nucleus; cy: cytoplasm; boxes: marginal ring region; white *: acrosomal granule/ acrosomal vesicle; av: acrosomal vacuole; a: acrosome; m: manchette microtubules; iam: inner acrosomal membrane; oam: outer acrosomal membrane; white arrow: ectoplasmic F-actin bundles. Scale: 2 μm.

Development • Supplementary information

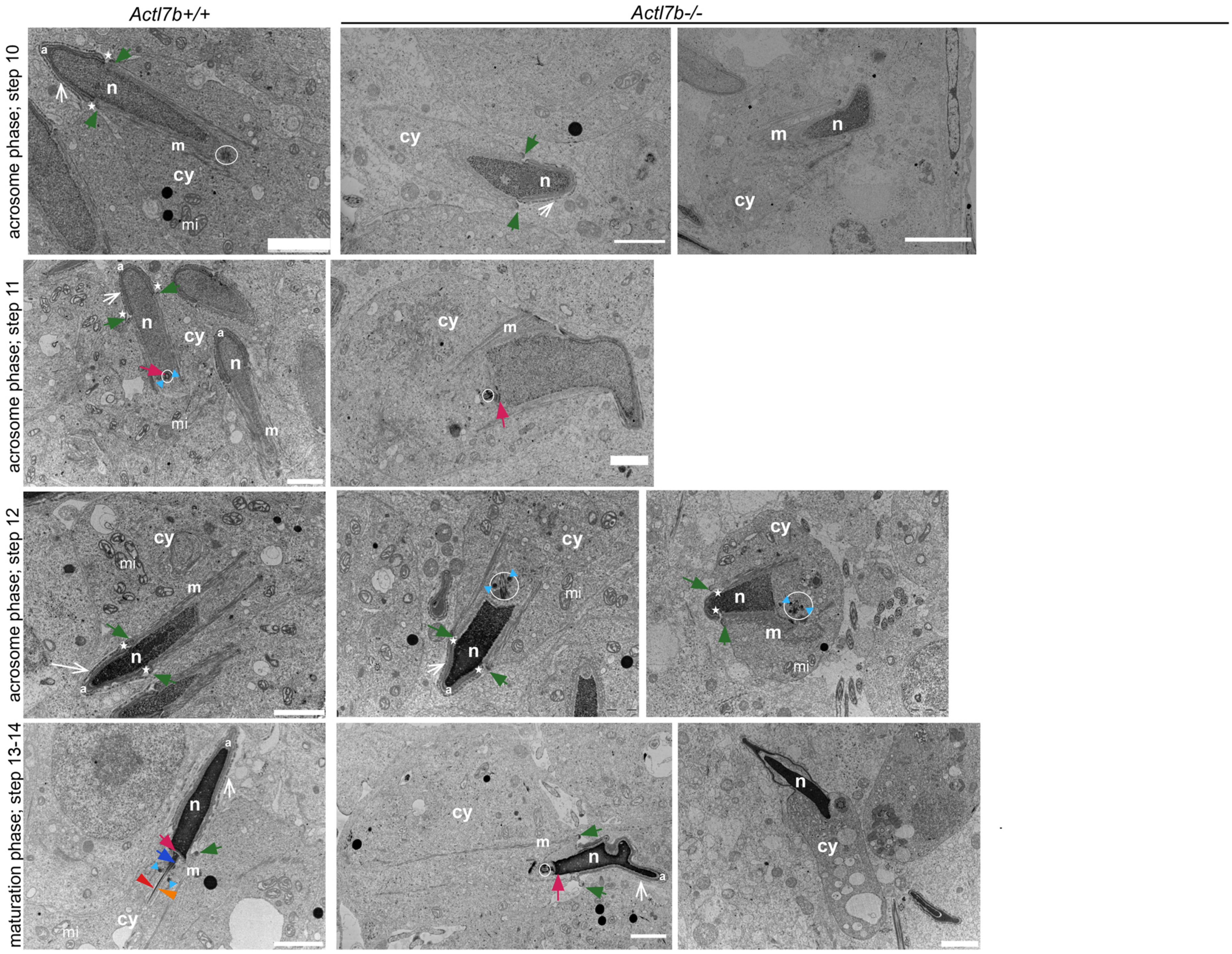

Fig. S8. Transmission electron micrographs of developmental steps 10-14 of spermiogenesis in WT and *Actl7b*-deficient mice. n: nucleus; cy: cytoplasm; boxes: marginal ring region; white *: acrosomal granule/acrosomal vesicle; a: acrosome; m: manchette microtubules; white arrow: ectoplasmic F-actin bundles; star: groove belt region; mi: mitochondria; encircled: proximal centriole; light blue arrow heads: annulus; green arrow: perinuclear ring; fuchsia arrow: basal plate; blue arrow: segmented column; red arrow head: axoneme; orange arrow heads: outer dense fibers. Scale: 2 µm.

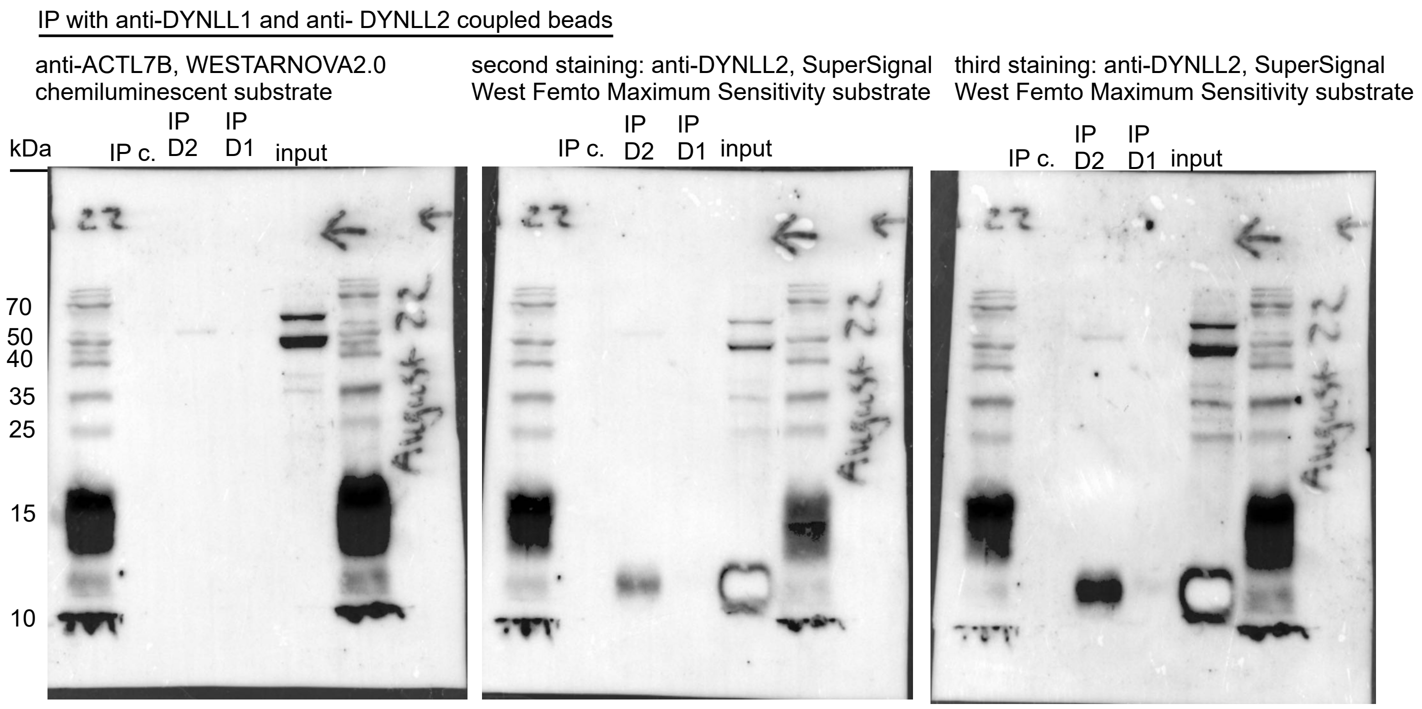

Fig. S9. Western blots of IPs using anti-DYNLL1 and anti-DYNLL2 coupled beads and bead only controls. Merged pictures of protein bands detected and colorimetric picture of the membrane with protein ladder. Note: The anti-ACTL7B antibody (13537-1-AP; Proteintech) has been shown to generate unspecific bands in western blot using mouse testis tissue subjected to SDS PAGE (see suppliers webpage). The specific band runs at a height of around 45 kDa.

Development • Supplementary information

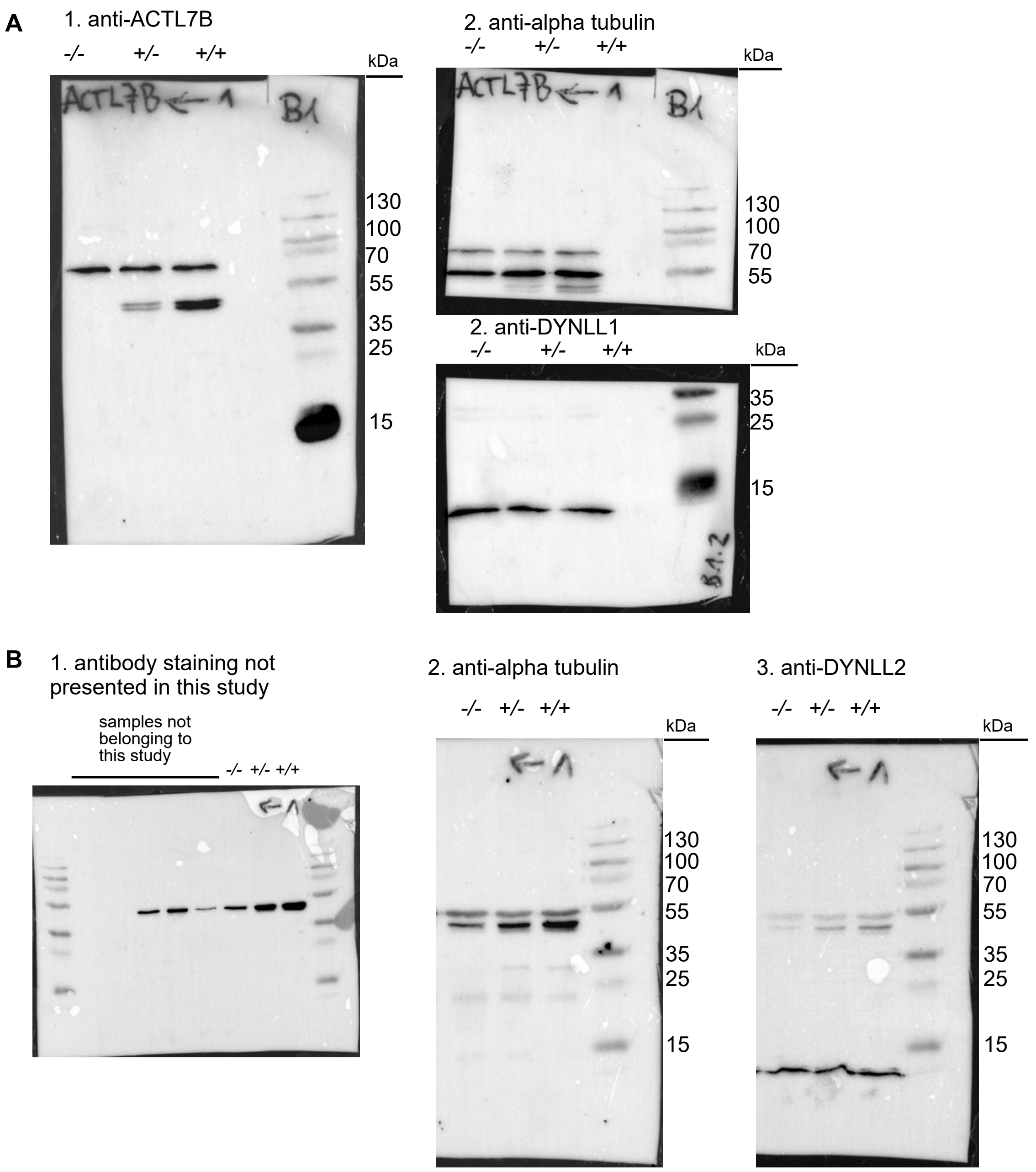

Fig. S10. Western blots on protein extractions from whole testis from *Actl7b+/+*, *Actl7b+/-* and *Actl7b-/-* mice. **(A)** Membrane stained with (1.) anti-ACTL7B, then cut in half to prevent cross-staining. Upper half was stained against alpha-tubulin and lower half was stained against DYNLL1. **(B)** Membrane stained with (1.) an antibody not presented in this study. Samples not belonging to this study are marked. The lanes presented in this study (croped) were stained against alpha-tubulin and subsequently DYNLL2. Ladder: Thermo Scientific™ PageRuler™ Plus Prestained Protein Ladder, 10 to 250 kDa. Merged pictures of protein bands detected and colorimetric picture of the membrane with protein ladder are shown.

Note: The anti-ACTL7B antibody (13537-1-AP; Proteintech) has been shown to generate unspecific bands in western blot using mouse testis tissue subjected to SDS PAGE (see suppliers webpage). The specific band runs at a height of around 45 kDa.

Development • Supplementary information

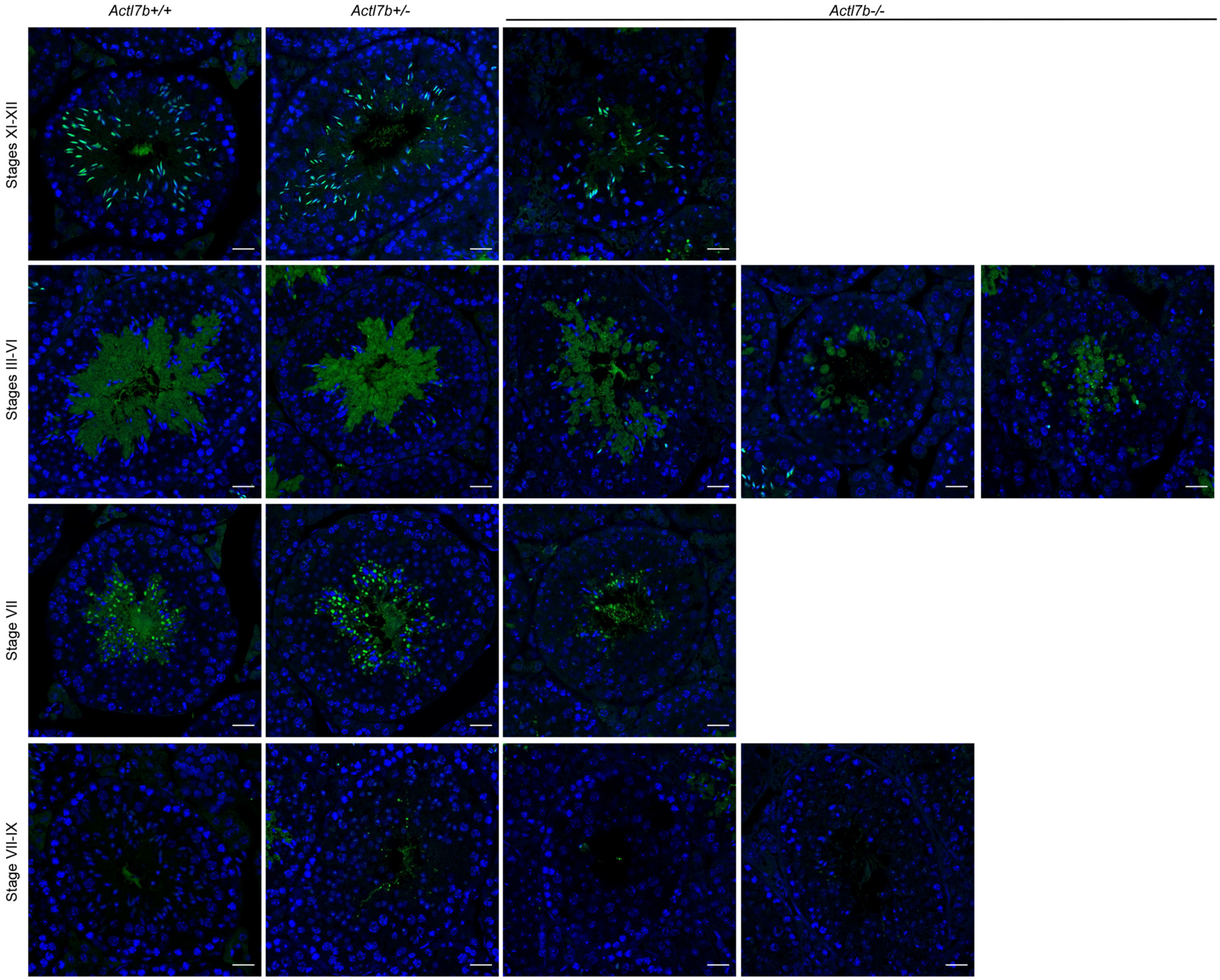

Fig. S11. Immunofluorescent staining against DYNLL1 in seminiferous tubules of *Actl7b*$^{+/+}$, *Aclt7b*$^{+/-}$ and *Actl7b*$^{-/-}$ *mice*. Scale: 20 µm.

Development • Supplementary information

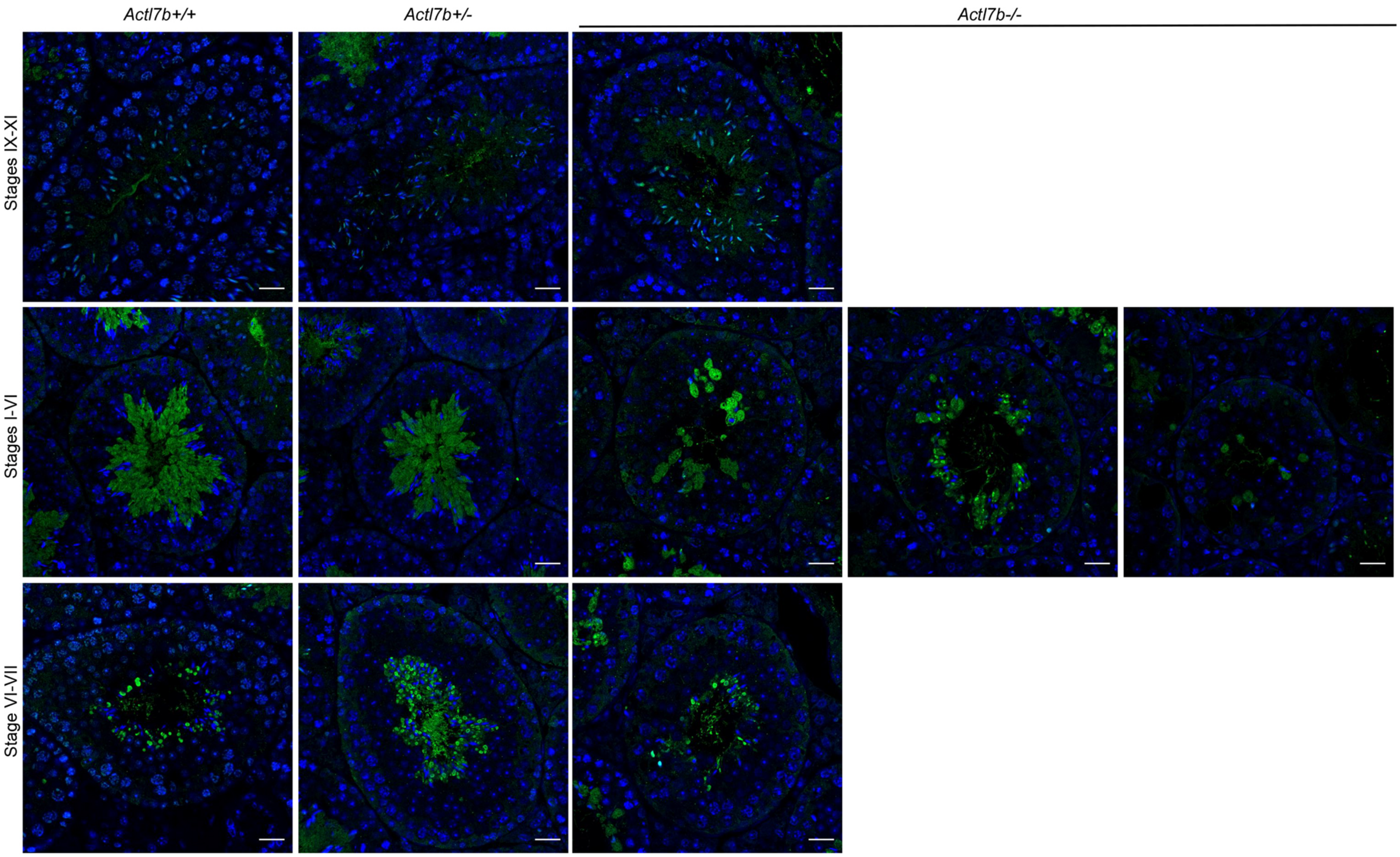

Fig. S12. Immunofluorescent staining against DYNLL2 in seminiferous tubules of *Actl7b*$^{+/+}$, *Aclt7b*$^{+/-}$ and *Actl7b*$^{-/-}$ *mice*. Scale: 20 μm.

Development • Supplementary information

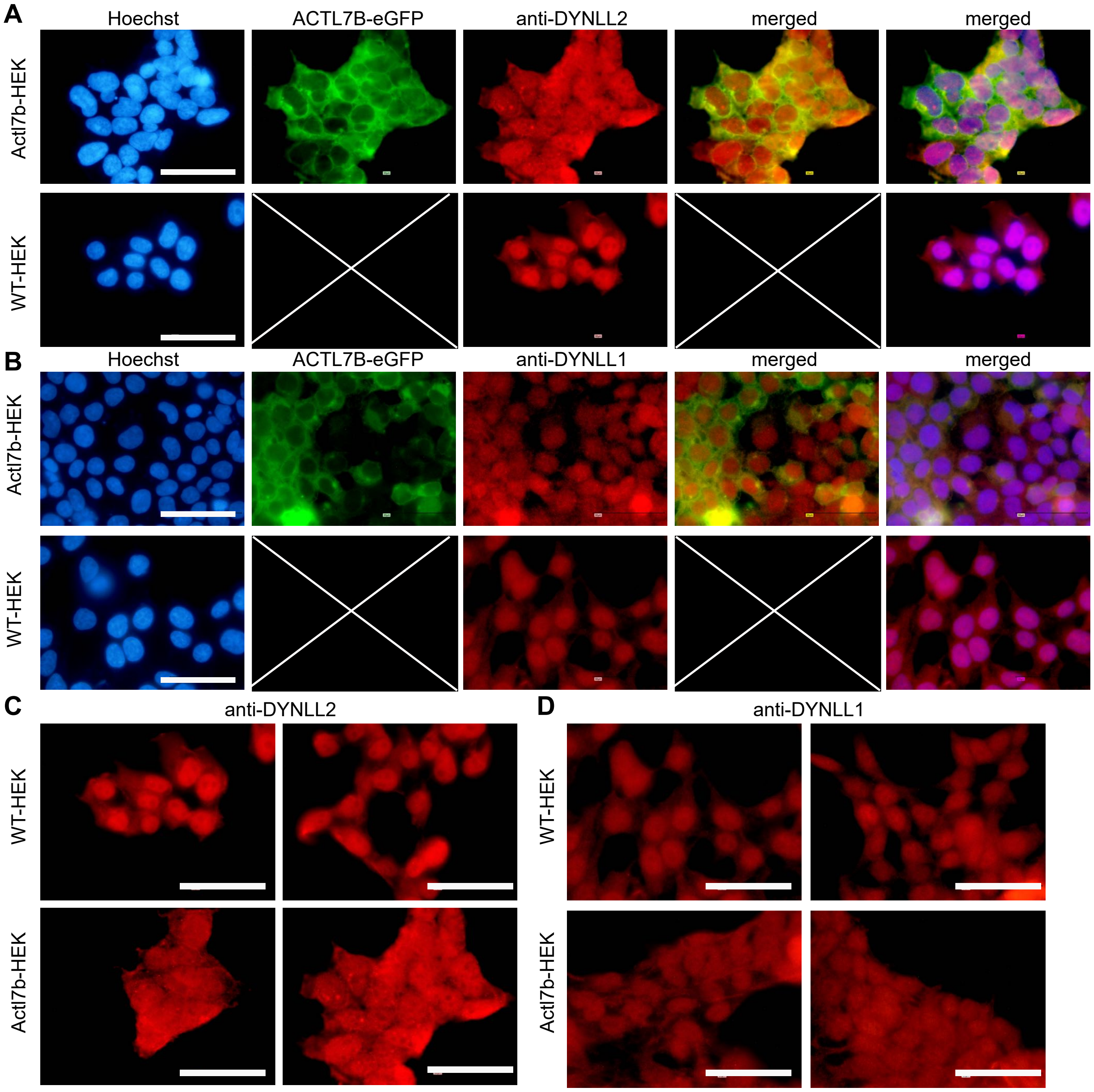

Fig. S13. ACTL7B and DYNLL1/2 *in vitro*. (A) IC staining against DYNLL2 in a stable ACTL7B-eGFP expressing HEK cell line and WT HEK cells. **(B)** IC staining against DYNLL1 in a stable ACTL7B-eGFP expressing HEK cell line and WT HEK cells. **(C)** IC staining against DYNLL2 in a stable ACTL7B-eGFP expressing HEK cell line and WT HEK cells. **(D)** IC staining against DYNLL1 in a stable ACTL7B-eGFP expressing HEK cell line and WT HEK cells. Scale: 50 µm

Development • Supplementary information

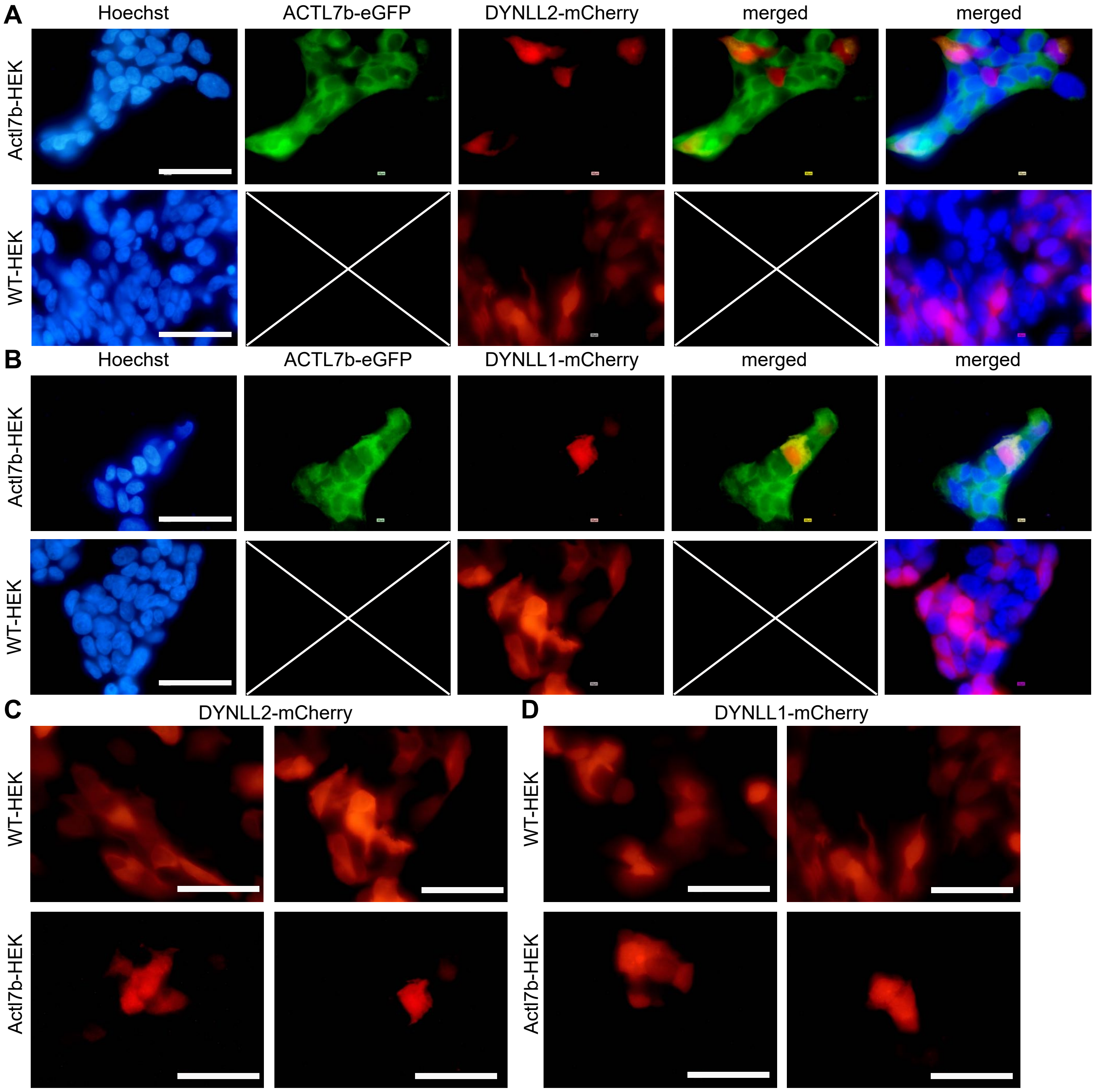

Fig. S14. ACTL7B and murine DYNLL1/2 *in vitro*. (A) Images of mCherry-tagged DYNLL2 in a stable ACTL7B-eGFP expressing HEK cell line and WT HEK cells. **(B)** Images of mCherry-tagged DYNLL1 in a stable ACTL7B-eGFP expressing HEK cell line and WT HEK cells. **(C)** Images of mCherry-tagged DYNLL2 in a stable ACTL7B-eGFP expressing HEK cell line and WT HEK cells. **(D)** Images of mCherry-tagged DYNLL1 in a stable ACTL7B-eGFP expressing HEK cell line and WT HEK cells. Scale: 50 µm.

Development • Supplementary information

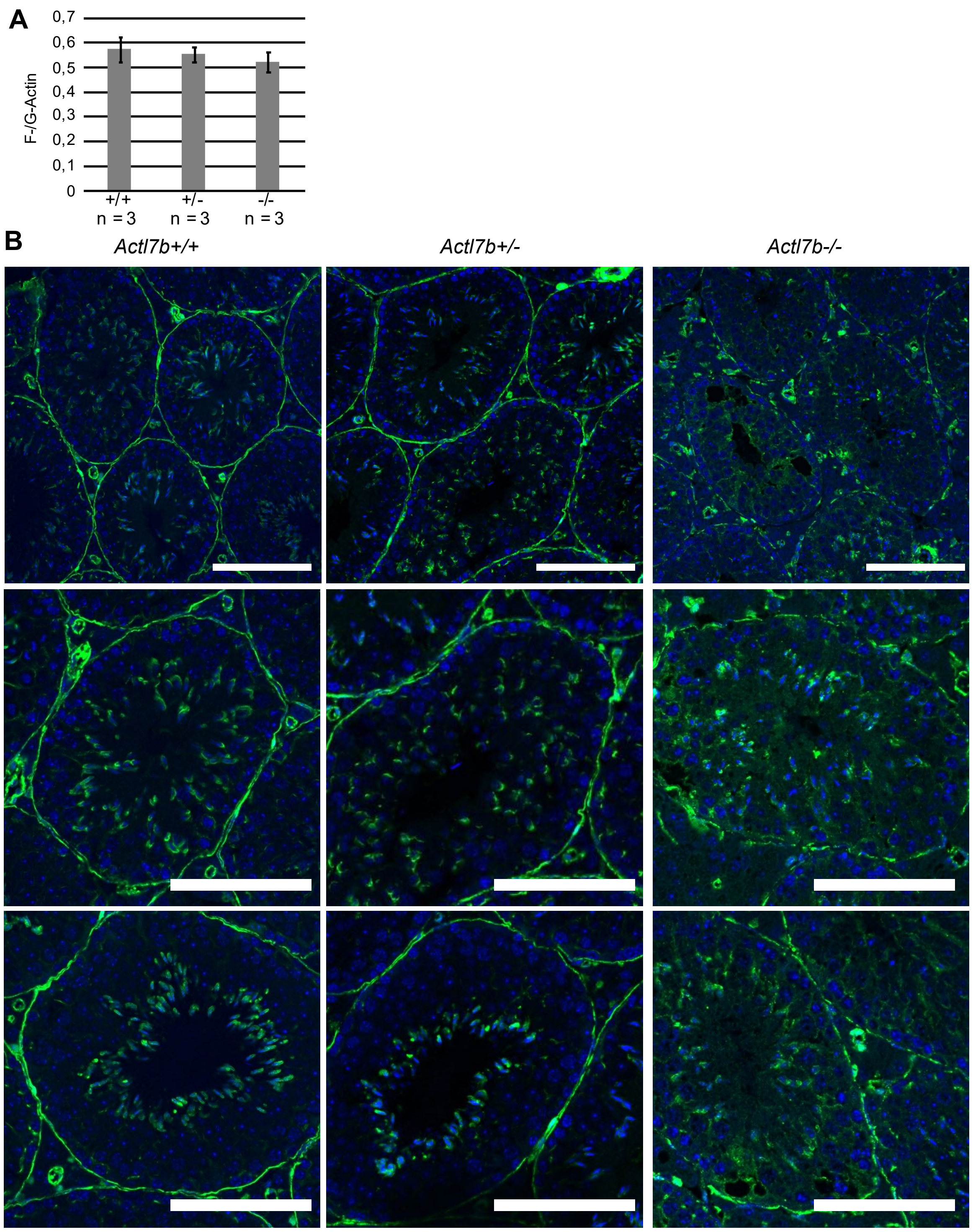

Fig. S15. Filamentous actin in seminiferous tubules of *Actl7b+/+*, *Aclt7b+/-* and *Actl7b-/- mice*. (A) F-/G-Actin ratio determined for whole testis extracts of *Actl7b+/+*, *Actl7b+/-* and *Actl7b-/-* mice. **(B)** Immunofluorescent staining of filamentous actin on *Actl7b+/+*, *Actl7b+/-* and *Actl7b-/-* testis. Scale: 100 µm.

Development • Supplementary information

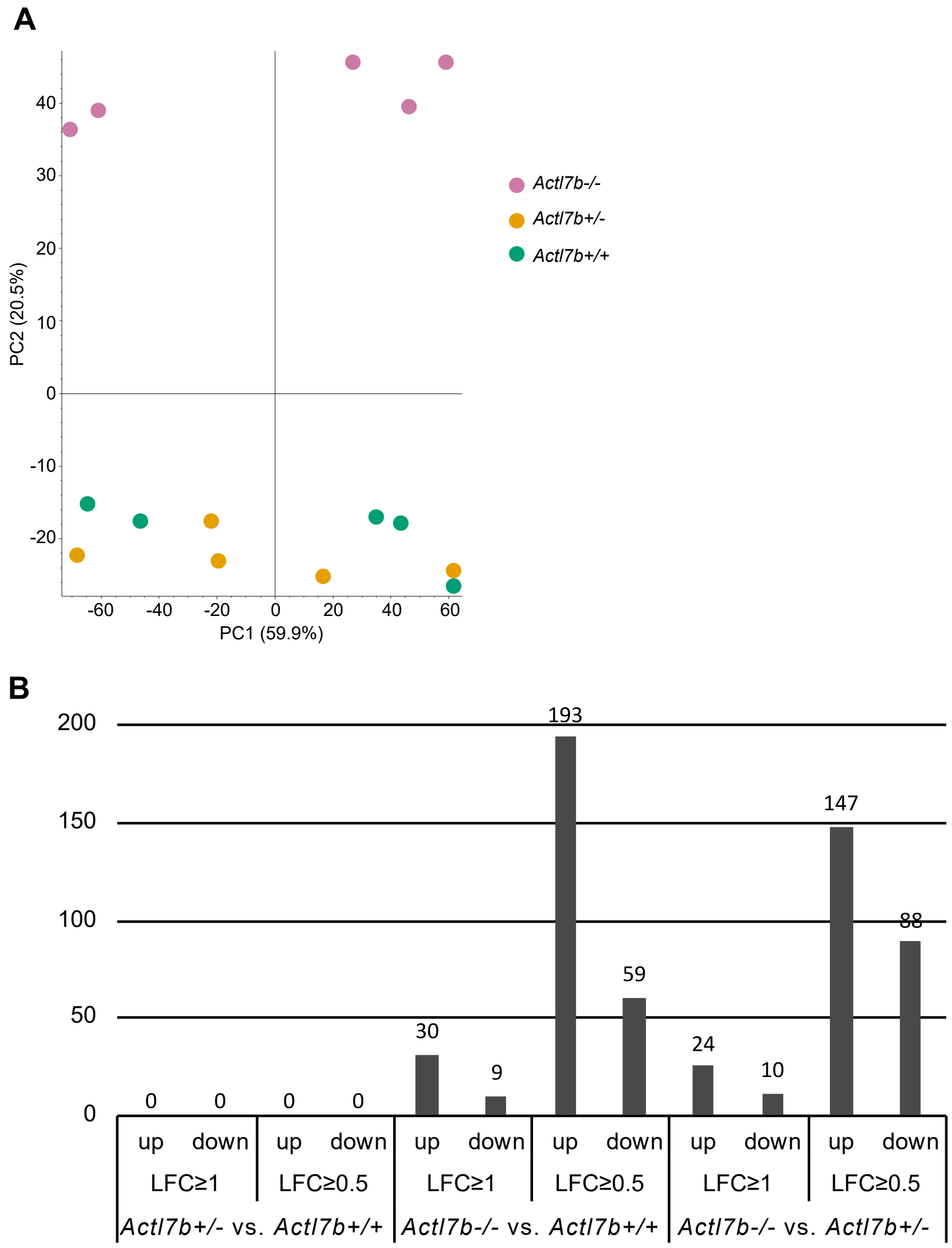

Fig. S16. Proteomic analysis of *Actl7b-/-*, *Actl7b+/-* and *Actl7b+/+* whole testis. (A) Principal component analysis (PCA) of *Actl7b+/+*, *Actl7b+/-* and *Actl7b-/-* testis (n = 5). **(B)** Number of differentially abundant (DA) proteins in *Actl7b+/-* vs. *Actl7b+/+*, *Actl7b-/-* vs. *Actl7b+/+* and *Actl7b-/-* vs. *Actl7b+/-* testis. The number of higher (up) and lower (down) abundant proteins are indicated. Results are given with an adjusted p-value ≤ 0.05 and a log2-fold change (LFC) of ≥1 and ≥0.05, respectively.

Development • Supplementary information

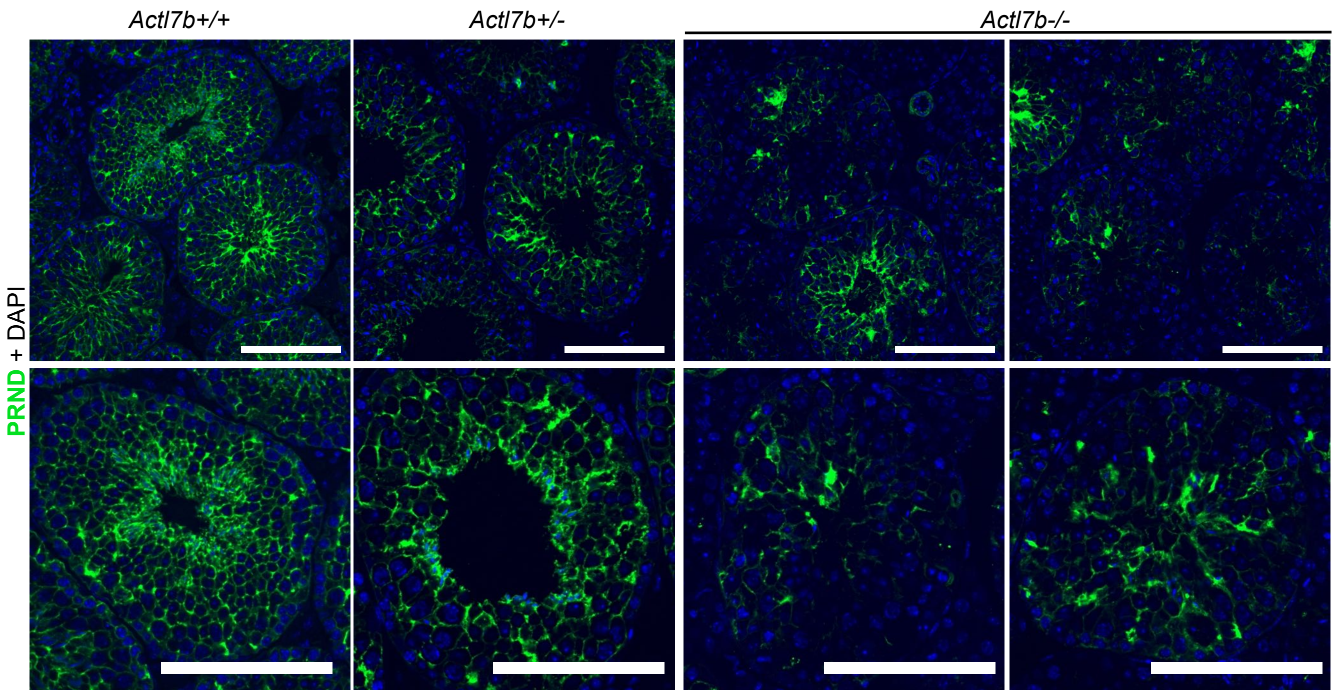

Fig. S17. IHC staining against PRND on *Actl7b*-deficient testicular sections. IHC stainings against PRND on *Actl7b+/+*, *Actl7b+/-* and *Actl7b-/-* testis sections. DAPI was used as the counterstain. Scale: 100 µm.

Development • Supplementary information

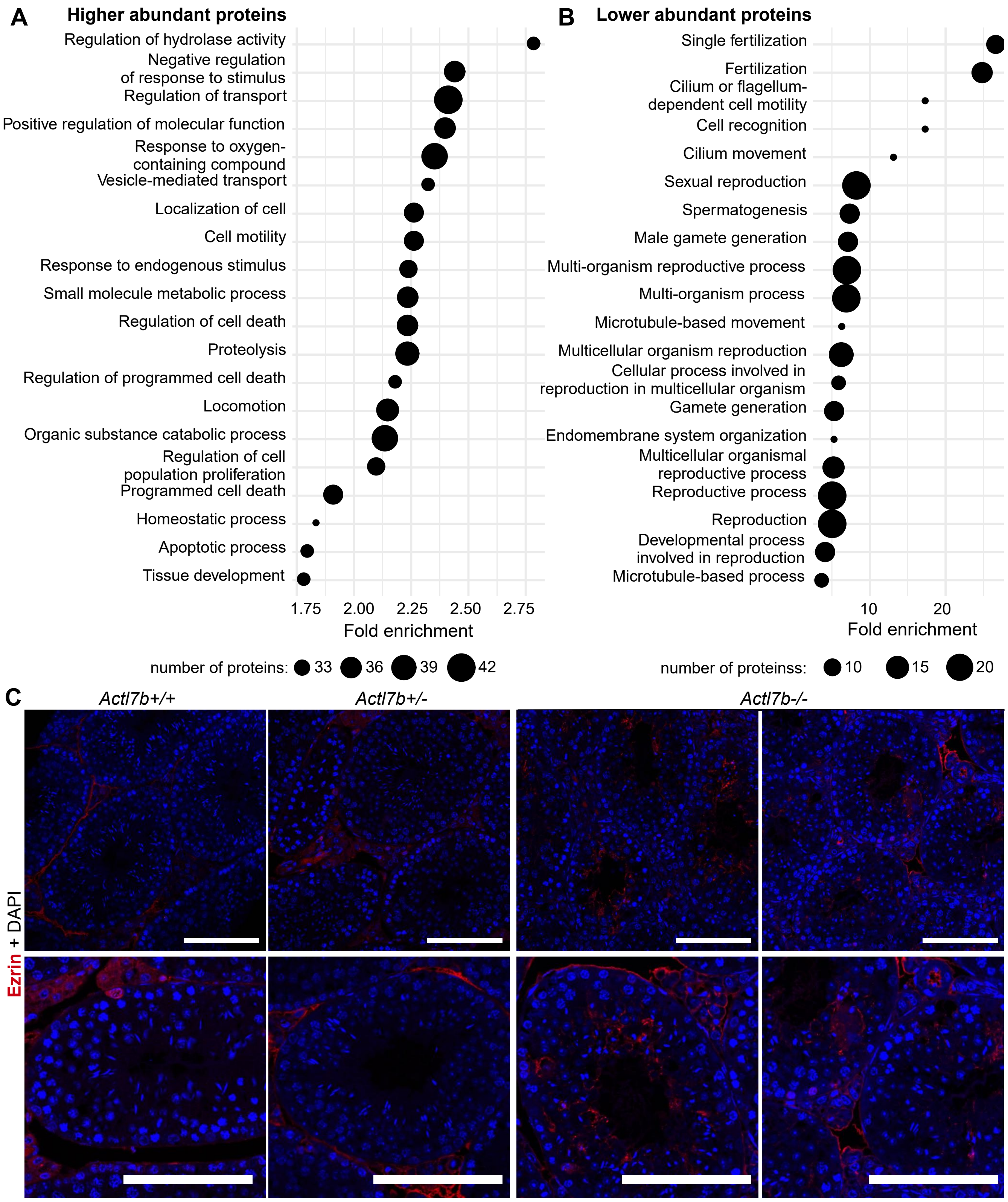

Fig. S18. Enrichment analysis and IHC staining against PRND on *Actl7b*-deficient testicular sections. (A-B) Dot plots of the top twenty gene ontology (GO)-biological process terms significantly enriched in the higher abundant **(A)** and lower abundant **(B)** proteins in *Actl7b-/-* vs. *Actl7b+/+* samples ($p \leq 0.05$, LFC ≤ 0.05). Dot sizes represent the number of proteins contributing to the GO-term. **(C)** IHC stainings against ezrin (red) on *Actl7b+/+*, *Actl7b+/-* and *Actl7b-/-* testis sections. DAPI was used as the counterstain. Scale: 100 μm.

Development • Supplementary information

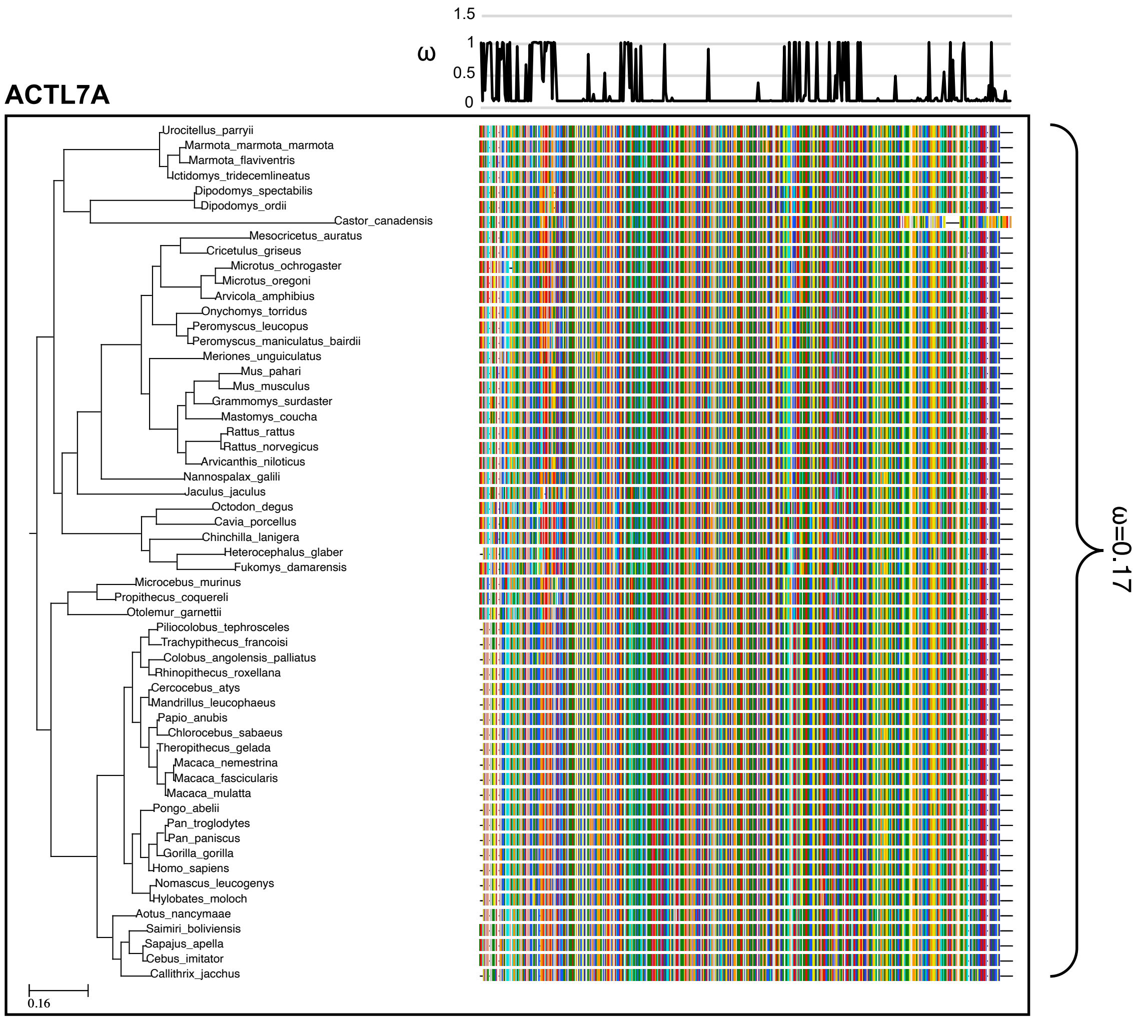

Fig. S19. Species phylogeny with branch length representing number of nucleotide substitutions per codon with schematic representation of ACTL7A amino acid alignment used in the PAML CodeML analysis. Evolutionary rate (ω) obtained by CodeML models M0 is shown for the whole alignment. The graph on top shows the evolutionary rate (ω) per codon sites across the whole tree (CodeML model M1a). See also: Table 1.

Development • Supplementary information

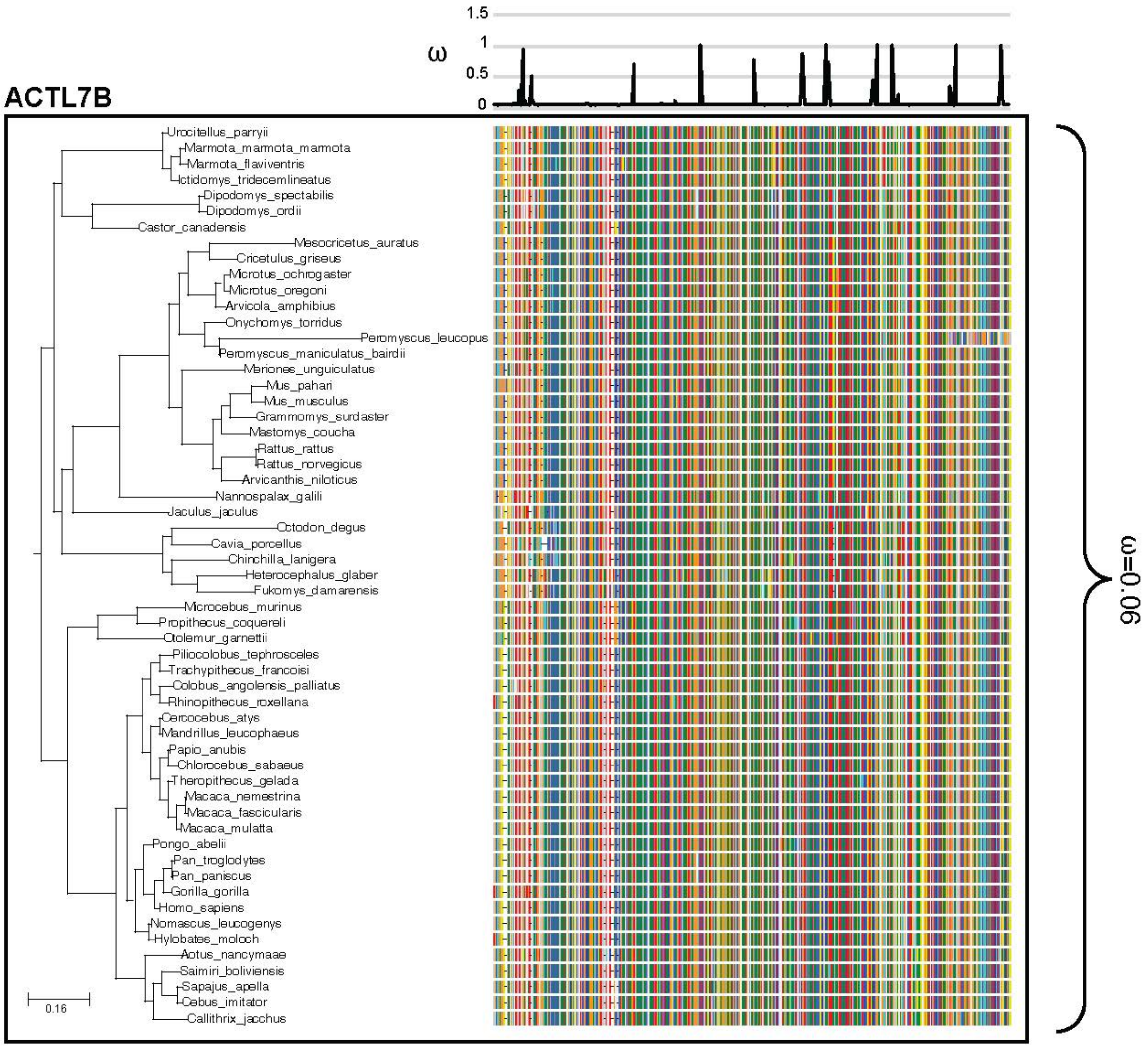

Fig. S20. Species phylogeny with branch length representing number of nucleotide substitutions per codon with schematic representation of ACTL7B amino acid alignment used in the PAML CodeML analysis. Evolutionary rate (ω) obtained by CodeML models M0 is shown for the whole alignment. The graph on top shows the evolutionary rate (ω) per codon sites across the whole tree (CodeML model M1a). See also: Table 1.

Development • Supplementary information

Table S1. Mass spectrometry datasets of proteins identified by ACTL7B CoIP using wild-type testes.

Available for download at
https://journals.biologists.com/dev/article-lookup/doi/10.1242/dev.201593#supplementary-data

Table S2. Dynll2 validated as interaction partner of ACTL7B by targeted analysis of mass spectrometry results from HEK cells expressing ACTL7B-GFP.

Available for download at
https://journals.biologists.com/dev/article-lookup/doi/10.1242/dev.201593#supplementary-data

Table S3. Mass spectrometry datasets of whole testes lysates from wild-type, Actl7b+/- and Aclt7b-/- male mice.

Available for download at
https://journals.biologists.com/dev/article-lookup/doi/10.1242/dev.201593#supplementary-data
