# Supplementary material for: Actl7b deficiency leads to mislocalization of LC8 type dynein light chains and disruption of murine spermatogenesis
Source: Development. 2023 Oct 27;150(21):dev201593. doi: 10.1242/dev.201593 (PMC10652042; doi:10.1242/dev.201593)
Supplement: Supplementary file 1 [file develop-150-201593_review_history.pdf]

Actl7b deficiency leads to mislocalization of LC8 type dynein light chains and disruption of murine spermatogenesis

Gina E. Merges, Lena Arévalo, Andjela Kovacevic, Keerthika Lohanadan, Dirk G. de Rooij, Carla Simon, Melanie Jokwitz, Walter Witke and Hubert Schorle
DOI: 10.1242/dev.201593

Editor: Haruhiko Koseki

Review timeline

Original submission:	9 January 2023
Editorial decision:	16 February 2023
First revision received:	26 April 2023
Editorial decision:	15 May 2023
Second revision received:	23 May 2023
Editorial decision:	14 June 2023
Third revision received:	14 September 2023
Accepted:	22 September 2023

Original submission

First decision letter

MS ID#: DEVELOP/2023/201593

MS TITLE: Actl7b-deficiency leads to mislocalization of LC8 type dynein light chains and disruption of murine spermatogenesis

AUTHORS: Gina Esther Merges, Lena Arevalo, Keerthika Lohanadan, Dirk G. de Rooij, Melanie Jokwitz, Walter Witke, and Hubert Schorle

I have now received all the referees' reports on the above manuscript, and have reached a decision. The referees' comments are appended below, or you can access them online: please go to BenchPress and click on the 'Manuscripts with Decisions' queue in the Author Area.

As you will see, the referees express considerable interest in your work, but have some significant criticisms and recommend a substantial revision of your manuscript before we can consider publication. If you are able to revise the manuscript along the lines suggested, which may involve further experiments, I will be happy receive a revised version of the manuscript. Your revised paper will be re-reviewed by one or more of the original referees, and acceptance of your manuscript will depend on your addressing satisfactorily the reviewers' major concerns. Please also note that Development will normally permit only one round of major revision. If it would be helpful, you are welcome to contact us to discuss your revision in greater detail. Please send us a point-by-point response indicating your plans for addressing the referee’s comments, and we will look over this and provide further guidance.

Please attend to all of the reviewers' comments and ensure that you clearly highlight all changes made in the revised manuscript. Please avoid using 'Tracked changes' in Word files as these are lost in PDF conversion. I should be grateful if you would also provide a point-by-point response detailing how you have dealt with the points raised by the reviewers in the 'Response to Reviewers' box. If you do not agree with any of their criticisms or suggestions please explain clearly why this is so.

© 2023. Published by The Company of Biologists under the terms of the Creative Commons Attribution License (https://creativecommons.org/licenses/by/4.0/).

Reviewer 1

Advance summary and potential significance to field

Spermiogenesis is a complicated cell morphological process that is important for giving rise to functional sperm, the male gamete. Abnormalities that occur during spermiogenesis are often the etiological causes for human infertility, particularly in cases showing decreased number, motility and malformation of sperm, collectively called oligo-atheno-terato-zoospermia (OAT) diseases. Intensive studies in the past have shown that numerous genes are involved in the regulation of spermiogenesis, however, much remain to be understood. The authors of this manuscript found that ACTIN like protein ACTL7B plays important roles during the elongation stage of spermiogenesis. Using gene targeting in mouse, they found that male Actl7b homozygous mutants are infertile, possibly due to the drastic defects in cellular morphogenesis of haploid spermatids, which carried malformed nuclei and flagella. Using biochemical and proteomics methods, they further showed that ACTL7B form complexes with dynein light chain proteins DYNLL1 and DYNLL2, suggesting an interesting link between cytoskeletal networks for the coordinated formation of sperm. Mutant testis contained varied levels of numerous proteins comparing to those of wild type and heterozygous controls. Their results showed an different functional roles of ACTL7B to its homolog (paralog) ACTL7A, which has been found to affect acrosome formation, adding an important clue to the understanding of how spermatogenic cells coordinate complexed morphological changes during spermiogenesis and what molecular players may be involved in this process.

Comments for the author

While the data presented in general are clear at macroscopic levels and suggestive to interesting mechanisms that warrant for further studies, results at the current stage are descriptive and short of in-depth mechanistic studies, some of the conclusions are speculative from the data, additional experiments may be needed to establish stronger ground.

Main concerns are:

1. Histological studies using non-specific staining for cell morphologies may not lead to conclusions described in the manuscript. For example, the authors conclude that, in Actl7b mutants, acrosomes are dis-organized (Fig. 4A), spermatids are engulfed by Sertoli cells (Fig. 3B-D) and residue bodies may not be formed properly (Fig. 5A), however, data showing the differences in these processes are mainly from microscopic observation. Some immuno-fluorescent staining coupled with confocal microscopy using cell or process-specific antibodies may be needed to back up these claims, e.g. PNA or acrosin staining for acrosomes, autophagy or lysosome marker staining for degradation, and purifications of residue bodies using BSA sedimentation method. In the same vein, changes of representative Sertoli and spermatids' proteins as identified in LC-MS/MS experiments should be verified using immuno-staining and Western blotting.

2. Co-immunoprecipitation experiments showing the complex formed between ACTL7B and DYNLL1/DYNLL2 should be verified using at least one other type of experiment, for example, in vitro binding assays. It is not explained in the manuscript why higher molecular complex maintained in denaturing gels during Western blotting (Fig. 6A-B), where no higher M.W. band was seen in n.b. fraction for DYNLL1 (Fig. 6A).

3. Relevant to the conclusion that sub-cellular localizations of DYNLL1/2 were changed in the absence of ACTL7B, the immunostaining showing in Fig. 6E-F seem different from staining shown in Fig. S6 and S7, in which wild type cells also contain granular structures in the cytoplasm at stage VII, the mutants only contained less. Is this due to the cells presented in Fig.6E-F are at different stages? Also, in lines 233-237, could co-immunostaining and confocal microscopy of ACTL7B and DYNLL1/2 be performed to see the sub-cellular distribution of them in wild type mice. Is the localization change a cause or a consequence of defected spermatids due to Actl7b deletion?

4. The link between Actin like proteins and microtubule through dynein light chain protein is very interesting, however, the comparison of F/G-actin, including the immunostaining shown in Fig. S8,

© 2023. Published by The Company of Biologists under the terms of the Creative Commons Attribution License (https://creativecommons.org/licenses/by/4.0/).

as well as Ezrin changes shown in Fig S10 are of low resolution. Better confocal microscopic images at higher magnifications and quantitative data may be needed.

Minor concerns:

1. Lines 160-161, Fig. 4A: What the “later stages” referring to? What are the upper and lower panels?

2. Lines 174-175, Fig. 5A: how is the “eviction of cytoplasm” different from wild type? The genotypes of mice used should be indicated on the graphs for better readability. A control image (wild type) may be included for data shown in Fig. 5C-F. In this figure and Fig. 3B-C and Fig. S4, what are the dark granules that look like gold particles used for immuno-staining?

3. Lines 184-186, the malformation of nuclei in the KO sperm is quite obvious, however, the conclusion of chromatin condensation defect may not be drawn from microscopic images (Fig. 4, Fig. S5), should immuno-staining of transition proteins or protamines reveal some differences?

4. Please check if all labels (arrows, arrowheads) are placed correctly (i.e. Fig. 3, Fig. 4 and Fig. S4).

5. Lines 206-208, only DYNLL1/2 is mentioned, what are the other proteins identified as positive hits in the Co-IP experiments?

6. Lines 224-225, while WB shows that DYNLL1/2 maintained in KO mice, staining of them shown in Fig. S6 and S7 appeared less than those of wild type, is this due to the loading of proteins on gel or the number of cells used to extract lysates?

7. Lines 253-255, lines 584-586, please indicate the number of mice/testes used and number of experimental repeats performed for mass spectrometry.

8. The off-target effects of sgRNAs should be examined in the mouse line used, at least for the top potential candidates.

9. Since the experiments were performed using fertilized eggs, “oocytes” should be changed to “fertilized eggs” or “zygotes” or “1-cell embryos”, e.g. in lines 455-458.

10. Lines 486, 489, what are the pH 6 and pH 9 for? Please indicate the antibodies as mouse monoclonal or rabbit polyclonal, etc. for the primary antibodies used in Materials and Methods.

11. Fig. 2A, how the pregnancy rate was calculated should be described in the Materials and Methods.

Please indicate the age of mice used for Fig. 2 (e.g. in the legend).

Reviewer 2

Advance summary and potential significance to field

In the present manuscript, Merges et al generated a knockout mouse model of Actl7b, an acrosome associated actin-like protein. They show homozygous loss of Actl7b results in male sterility, due to defects in spermiogenesis and the production of abnormal sperm. The strength of the paper is its proteomics analysis; however the histological (light and EM level) characterisation of spermatogenesis needs to be improved for it to be acceptable for publication.

In regard to novelty, the authors are unfortunate in their timing as a characterisation of male infertility in an Actl7b knockout mouse has just been published within the last month in the Biology of Reproduction (Clement et al 2023). However, the proteomics aspects of the current paper will still add valuable and novel information to field. The Clement et al 2023 paper, also focussed on a

© 2023. Published by The Company of Biologists under the terms of the Creative Commons Attribution License (https://creativecommons.org/licenses/by/4.0/).

flagella defect not characterised herein, as opposed to the acrosome defects that the current paper suggests. As such, ensuring the acrosome phenotype is properly characterised is essential.

Comments for the author

1. The authors will need to update the manuscript throughout to integrate the new insights from the recently published Actl7b paper in the Biology of Reproduction.

2. Please include a comment on overall health of mice, body weight data etc

3. Lines 142 ‘ Late stage spermatids show abnormal morphologies’ – this is vague need to clarify what abnormalities you are referring.

4. Fig 3 needs to be improved to include comparison control images, to ensure all differences reported in KO are valid and to illustrate the normal light and EM histology.

5. In Fig 3B no lumen is evident suggesting it is not a perfect transverse section through a seminiferous tubule, as such the appearance of multiple stages of spermatids within one tubule needs to be interpreted with caution. It should be confirmed using PAS-stained testis sections and EM sections wherein the lumen is evident.

6. The appearance of flagellar structures throughout the epithelium in 3B should also not be over interpreted. Flagella development initiates in early spermiogenesis. As such, round and elongating spermatid flagella are often projecting into the lumen from deep within the epithelium depending on the stage and the position of the spermatids. Noting there are some stages when spermatids are drawn close to the basement membrane (i.e., step 15 spermatids in stage V).

7. More care needs to be taken with the formatting of many of the figures.
Please include seminiferous tubule stage and genotype on all figures. For many of the EM images, the authors should crop them appropriately i.e., to focus specifically on the structure/cell of interest.

8. Lines 149-153 – the authors have only included sperm counts and the percentage of viable sperm for heterozygous and WT males. The counts for Actl7b-/- males should also be included (even if is very low).

9. The authors should try to include data showing morphology of epididymal sperm. Swim out or black flush into a small volume (0.5 ml) for sperm smears for staining.

10. The reduction in testis weight suggests a loss of germ cells in spermatogonia and/or spermatocytes, this is because spermatids, particularly in final half of spermiogenesis, contribute minimally to the overall weight of the testis. This would also be consistent with the authors observations of vacuoles near the basement membrane The authors should assess whether spermatogonia or meiosis is abnormal. Indicative of abnormal meiosis a double nucleated spermatids is evident in Fig 5E.

11. Line 163 – ‘generally, less spermatids develop in Actl7b-/- testis.’ This statement should be supported by a quantitative assessment of testis daily sperm production (which is distinct from epididymal sperm content)

12. The characterisation of the acrosome phenotype is an integral part of this paper and needs to be improved.

a. The power of the images in 4A is insufficient for a proper assessment of acrosome morphology and PAS-positive vesicles. These should be taken at 100x and Golgi phase should also be assessed. The authors should quantify the number of spermatids with abnormalities at the elongated spermatid stage.
b. Fig S3, why is no step 3 KO presented? Higher magnification insets focussing just on the acrosome. Golgi and acroplaxome are needed.

© 2023. Published by The Company of Biologists under the terms of the Creative Commons Attribution License (https://creativecommons.org/licenses/by/4.0/).

c. Fig S4, again higher magnification insets focussing just on the acrosome and acroplaxome are needed, and step 13-14 ko panel is missing. Many of the elongating spermatids are incorrectly staged. The ‘step 9’ WT and the two far right ‘step 9’ Actl7b-/- spermatids are is at least step 10 or 11 based on their shape. The two far right two step ‘12’ Actl7b-/- spermatids are at least step 13 based on their shape and the manchette position.
13. Line ‘178-179’ ‘the general architecture of elongating spermatids appears disrupted’ - please be more specific in your description

14. Lines 181-182 ‘a subset of spermatids lacks the manchette, several spermatids show partly detached ectoplasmic F-Actin bundles’. This is not clear in the presented images. Higher magnification is needed. The manchette phenotypes should be confirmed with IHC of tubulin on testis sections.

15. Fig 5A, please include appropriate control comparison images.

16. The characterisation of flagella defects if included needs to be improved.
In Lines 187-188 the authors state ‘the proximal centriole locates to the posterior region of the nucleus to form the basal body, but often the extension of the axoneme fails in Actl7b-/- spermatids. In later stage spermatids the flagellum is absent. However, cross- sections of properly formed flagella can be found detached from spermatids’

These observations seem to rely solely on EM, given that the plane of section in EM can sometimes mean that HTCA is not observed the authors should use another method to confirm this (e.g. epididymal sperm morphology, or spermatid dry downs staining for the axoneme). The authors should give an indication of the frequency of these phenotypes. As highlighted in the other comments, for the EM higher magnification images should be included to allow ultrastructure of the sperm tail to be more readily assessed by readers.

17. For the MS, why was KO testis not used as the control?

18. Lines 265 -266, might the increase in Sertoli cell proteins just be a function of the decreased amount of germ cells in the epithelium i.e., resulting in a higher ration of Sertoli cells to germ cells?

19. As a general comment the authors need to be careful in the description of their results, there is a tendency to make sweeping/vague statements. Need to be more precise in you wording, citing specific arrows on specific panels of figures in the in text description would help.

Reviewer 3

Advance summary and potential significance to field

This is an exciting paper examining the role of a testis specific Actin-related proteins (Arp), ACT7LB, a highly conserved protein in primates and rodents in spermiogenesis. ACTL7B is specifically expressed in round and elongating spermatids, and the Actl7b-KO mice are infertile and display defects in sperm formation, cytoskeleton organization, and chromatin condensation, leading to spermatid arrest or loss at various stages of the seminiferous tubule cycle. To better understand the role of ACT7LB the authors performed IP-MS and find that ACT7LB interacts with dynein protein, which implicate ACT7LB and Dynein’s in the global sperm remodeling process. This is an important body work, but additional experiments, clarifications and validations are needed to strengthen the work. Please see comments below:

1) It seems that prm2 in the mutant is cytoplasmic and not nuclear? Is that correct?

2) The EM images in 4C seem that the Actl7b-KO spermatids more dense – more contrast. Is this an imaging staining artifact or an indication of abnormal packaging?

3) In figure 5 including reference panels for WT and also using similar magnification for WT/Mutant cell comparisons is necessary to make informed decisions by reviewer.

© 2023. Published by The Company of Biologists under the terms of the Creative Commons Attribution License (https://creativecommons.org/licenses/by/4.0/).

4) In figure 6 — why do the eluate bands run much higher than the input? The bands are not as convincing.

5) Is the reduction in figure 6 for ACTl7A and Hook1 just due to the loss of round and elongating spermatid populations. Given the proportion of different cell populations differ between the WT and mutant – wouldn’t it be better to sort different populations from WT and mutant and run westerns on equivalent number of cells from each type? This will give a better representation of the changes in protein levels in respective population. This approach will work if these proteins are broadly expressed in spermatids etc… because then you won’t have population loss as a confounding factor.

6) The file I downloaded had missing supplemental figures

7) In Figure 6E and F – is the mislocalization of DynLL1/2 just because spermatids do not shed the cytoplasmic droplet? If you compare the mutant spermatids in KO to an earlier stage in WT, do you see the same pattern? If spermatids stop progressing the stage your analyzing in the mutant may not match exactly the WT?

8) Validating proteomic differences on pure populations will circumvent the differences in cell populations in the wt, het and mutant testis.

Comments for the author

This is an exciting paper examining the role of a testis specific Actin-related proteins (Arp), ACT7LB, a highly conserved protein in primates and rodents in spermiogenesis. ACTL7B is specifically expressed in round and elongating spermatids, and the Actl7b-KO mice are infertile and display defects in sperm formation, cytoskeleton organization, and chromatin condensation, leading to spermatid arrest or loss at various stages of the seminiferous tubule cycle. To better understand the role of ACT7LB the authors performed IP-MS and find that ACT7LB interacts with dynein protein, which implicate ACT7LB and Dynein’s in the global sperm remodeling process. This is an important body work, but additional experiments, clarifications and validations are needed to strengthen the work. Please see comments below:

1) It seems that prm2 in the mutant is cytoplasmic and not nuclear? Is that correct?

2) The EM images in 4C seem that the Actl7b-KO spermatids more dense – more contrast. Is this an imaging staining artifact or an indication of abnormal packaging?

3) In figure 5 including reference panels for WT and also using similar magnification for WT/Mutant cell comparisons is necessary to make informed decisions by reviewer.

4) In figure 6 – why do the eluate bands run much higher than the input? The bands are not as convincing.

5) Is the reduction in figure 6 for ACTl7A and Hook1 just due to the loss of round and elongating spermatid populations. Given the proportion of different cell populations differ between the WT and mutant – wouldn’t it be better to sort different populations from WT and mutant and run westerns on equivalent number of cells from each type? This will give a better representation of the changes in protein levels in respective population. This approach will work if these proteins are broadly expressed in spermatids etc… because then you won’t have population loss as a confounding factor.

6) The file I downloaded had missing supplemental figures

7) In Figure 6E and F – is the mislocalization of DynLL1/2 just because spermatids do not shed the cytoplasmic droplet? If you compare the mutant spermatids in KO to an earlier stage in WT, do you see the same pattern? If spermatids stop progressing the stage your analyzing in the mutant may not match exactly the WT?

© 2023. Published by The Company of Biologists under the terms of the Creative Commons Attribution License (https://creativecommons.org/licenses/by/4.0/).

8) Validating proteomic differences on pure populations will circumvent the differences in cell populations in the wt, het and mutant testis.

First revision

Author response to reviewers' comments

Dear Reviewers,

Thank you very much for reviewing our manuscript and providing us with your comments. We have done extensive re-writing and experimentation in order to include all your comments. We believe, that your input helped to significantly improve our work and add some more aspects to the phenotype.
Please find below a point by point rebuttal, as well as a marked up copy of the manuscript.

Reviewer 1 Advance Summary and Potential Significance to Field:
Spermiogenesis is a complicated cell morphological process that is important for giving rise to functional sperm, the male gamete. Abnormalities that occur during spermiogenesis are often the etiological causes for human infertility, particularly in cases showing decreased number, motility and malformation of sperm, collectively called oligo-atheno-terato- zoospermia (OAT) diseases. Intensive studies in the past have shown that numerous genes are involved in the regulation of spermiogenesis, however, much remain to be understood. The authors of this manuscript found that ACTIN like protein ACTL7B plays important roles during the elongation stage of spermiogenesis. Using gene targeting in mouse, they found that male Actl7b homozygous mutants are infertile, possibly due to the drastic defects in cellular morphogenesis of haploid spermatids, which carried malformed nuclei and flagella. Using biochemical and proteomics methods, they further showed that ACTL7B form complexes with dynein light chain proteins DYNLL1 and DYNLL2, suggesting an interesting link between cytoskeletal networks for the coordinated formation of sperm. Mutant testis contained varied levels of numerous proteins comparing to those of wild type and heterozygous controls. Their results showed an different functional roles of ACTL7B to its homolog (paralog) ACTL7A, which has been found to affect acrosome formation, adding an important clue to the understanding of how spermatogenic cells coordinate complexed morphological changes during spermiogenesis and what molecular players may be involved in this process.

Reviewer 1 Comments for the Author:
While the data presented in general are clear at macroscopic levels and suggestive to interesting mechanisms that warrant for further studies, results at the current stage are descriptive and short of in-depth mechanistic studies, some of the conclusions are speculative from the data, additional experiments may be needed to establish stronger ground. Main concerns are:

1. Histological studies using non-specific staining for cell morphologies may not lead to conclusions described in the manuscript. For example, the authors conclude that, in Actl7b mutants, acrosomes are dis-organized (Fig. 4A), spermatids are engulfed by Sertoli cells (Fig. 3B-D) and residue bodies may not be formed properly (Fig. 5A), however, data showing the differences in these processes are mainly from microscopic observation. Some immuno-fluorescent staining coupled with confocal microscopy using cell or process-specific antibodies may be needed to back up these claims, e.g. PNA or acrosin staining for acrosomes, autophagy or lysosome marker staining for degradation, and purifications of residue bodies using BSA sedimentation method. In the same vein, changes of representative Sertoli and spermatids' proteins as identified in LC-MS/MS experiments should be verified using immuno-staining and Western blotting.

© 2023. Published by The Company of Biologists under the terms of the Creative Commons Attribution License (https://creativecommons.org/licenses/by/4.0/).

Authors reply: We address these comments in different sections of the manuscript and hope to address all points sufficiently.

Figure 4 has been completely changed in the course of this Revision. PAS pictures have been replaced by images of PNA stained sections to evaluate acrosomal structures in more detail (see Fig. 4). See lines 197-201:
Since, ACTL7B is first present in round spermatids, effects were expected to manifest from round spermatid stage onwards. In comparison to *Actl7b+/-* and *Actl7b+/+*, in *Actl7b-/-* testis, acrosomal structures are less frequent and disorganized after spermatids start to elongate (Fig. 4A, Fig. S4A-B). In Golgi and Cap phase *Actl7b-/-* acrosomal structures appear normal, abnormalities become apparent in Maturation phase (Fig. 4A).
The following section has been added to the method section (lines 596-600):
Peanut agglutinin (PNA)-fluorescein isothiocyanite (FITC) Alexa 615 Fluor 488 conjugate (Molecular Probes, Invitrogen, Waltham, USA) was used on deparaffinized sections who were fixed using Bouin . After permeabilization with 0.1% Triton-X 100 for 5 min at RT, slides were blocked for 1 h with 1% BSA and incubated for 1 h with PNA (1:200). Slides were mounted with or ROTI Mount FluorCare DAPI (Carl Roth).

To look at autophagy markers we added western blots to Fig. 3.
The following section was added to the result part (lines 172-178):
Of note, we found that a mammalian autophagy marker, microtubule-associated protein 1A/1B-light chain 3 (LC3) and cathepsin B (CTSB) levels significantly increased in *Act7b-/-* testis (Fig. 3D). Interestingly, deletion of CTSB in mice leads to inhibition of autophagy and promotion of apoptosis in murine testis, suggesting CTSB to be a regulator between autophagy and apoptosis during spermatid development (Wen et al., 2022). Hence, based on our data we favor autophagy to be the main cause of spermatid degradation.
See also lines 331-332:
Of note CTSB was also shown to be higher abundant via western blot (Fig. 8D, Fig. 3D). See also lines 348-350:
The autophagy-related protein LC3B protein levels, which were shown to be increased in *Actl7b-/-* testis compared to *Actl7b+/+* testis via western blot (Fig.3D), was also identified as higher abundant in mass spec (*Map1lc3b*; Table S1).
The following section was added to the discussion part (lines 460-463):
We show that key autophagy marker proteins show higher levels in *Actl7b-/-* testes. Since TUNEL staining was negative, we favor autophagy to be the main cause of spermatid degradation. Even so, apoptosis related proteins were also found to be higher abundant in *Actl7b-/-* testis.
The following section was added to the method part (lines 601-603):
TUNEL assay was performed utilizing the TUNEL Assay Kit- HRP-DAB (Abcam, ab206386) according to manufacturers instructions. Positive control slides were treated with 1 μg/μl DNase I in TBS/ 1 mM $MgSO_4$ for 20 min at RT as recommended by the manufacturer.

To address the presence of residual bodies we added a staining against cleaved protamine 2.
See lines 218-221:
Staining against cP2 (to visualize residual cytoplasm) on caput epididymal sections showed, that large amounts of cP2 are retained in the cytoplasm of immature germ cells in *Actl7b-/-* mice (Fig. 5C). This again suggests defects in the eviction of cytoplasm.
We repeated the staining against ezrin and imaged the testis sections with a confocal microscope (Fig. S18). We believe that we were able to validate the results gathered with mass spec. The same is true for other proteins validated (CTSB, Fig. 3D; PRND, Fig. S17)

2. Co-immunoprecipitation experiments showing the complex formed between ACTL7B and DYNLL1/DYNLL2 should be verified using at least one other type of experiment, for example, in vitro binding assays.
It is not explained in the manuscript why higher molecular complex maintained in denaturing gels during Western blotting (Fig. 6A-B), where no higher M.W. band was seen in n.b. fraction for DYNLL1 (Fig. 6A).

Authors reply: To validate ACTL7B-DYNLL1/2 interaction, we generated a stable *Actl7b- eGFP*

© 2023. Published by The Company of Biologists under the terms of the Creative Commons Attribution License (https://creativecommons.org/licenses/by/4.0/).

expressing HEK cell line. Staining of the endogenous human DYNLL1 and DYNLL2 showed, that both light chains show a different immunolocalization when ACTL7B is present compared to WT HEK cells (see Fig. 7E, Fig. S13). This was also seen when transfecting the cells transiently with murine DYNLL1 or DYNLL2 fused to mCherry (see Fig. S14).
Additionally, we were able to pull down DYNLL2 using GFP trap beads followed by western blot (see Fig. 6C). The same higher molecular weight complex was identified as seen for whole testis extractions.

Additions made to Results part:

Lines 273-281:

Next, we generated HEK cells stably expressing *Actl7b* fused to *eGFP* (HEK$^{Actl7b\text{-}eGFP}$) and performed a pull-down with GFP nanobody-coupled beads on whole protein extracts (Fig. 6C, Fig. S10). Protein extracts from WT HEK cells were used as control. We identified ACTL7B via Western blot both in the input and the IP eluate. ACTL7B was, as expected, not identified in the WT cells. Western blot using the DYNLL2 antibody identified multiple bands in the input from both cell lines, corresponding to the size of DYNLL2 alone and potential protein complexes of larger size. In the IP eluate from HEK$^{Actl7b\text{-}eGFP}$ cells a band positive for DYNLL2 was identified at the size of around 60 kDa, suggesting that DYNLL2 is bound to ACTL7B.

Lines 297-305:

In HEK$^{Actl7b\text{-}eGFP}$ cells, ACTL7B localized mainly to the cytoplasm and DYNLL1 and DYNLL2 were localized throughout the whole cytoplasm and the staining appeared fibrous (Fig. 7E-F, Fig. S13). In comparison, DYNLL1 and DYNLL2 localized mainly in the nucleus in WT HEK cells and only weakly in the cytoplasm. Further the staining looked much more even in WT HEK cells. Next, we transfected HEK cells with an expression plasmid containing the murine DYNLL1 or DYNLL2 fused to mCherry. Here, a similar staining pattern can be seen (Fig. S14). This supports the notion that ACTL7B interacts with and controls DYNLL1 and DYNLL2 localization in the cell.

Additions made to Discussion part (lines: 439-440):

Further, we show that expression of *Actl7b* in HEK cells alters DYNLL1 and DYNLL2 localization in the cell.

Additions made to Methods section:

Lines 689-719:

Generation of HEK$^{Actl7b\text{-}eGFP}$ cells

Actl7b CDS was amplified using the Q5 high fidelity thermostable DNA polymerase (NEB) from mouse testis cDNA using the following primers (GFP-Actl7b_fw and GFP-Actl7b_rv: ctcgagctcaagcttcgatggcgacaaagaacag, ggtaccgtcgactgcagttagcacttgctgtagatgg) and inserted into the pEGFP-C1 vector (Clontech) digested with EcoRI (NEB) using the NEBuilder HiFi DNA Assembly kit (NEB) according to the manufacturer's instructions. The pEGFP-C1-Actl7b plasmid was then linearized with ApaLI (NEB) and transfected into HEK293 cells using Lipofectamine™ 2000 (ThermoFisher Scientific). Next, cells were selected for 3 weeks with 1 mg/ml geneticin™ (G418 sulfate, Gibco), trypsinized, diluted to 0.5 cells/100 µl and plated 100 µl/well on a 96-well plate. After 6 weeks five clones with different degrees of fluorescence were selected, expanded in 0.8 mg/ml geneticin™ and cryopreserved. Protein lysates of the five clones were tested by western blotting with polyclonal anti-ACTL7B (Invitrogen, PA5-113560) and anti-GFP (kind gift from Prof. Dr. Oliver Gruss, Institute of Genetics, Bonn University). All clones showed different expression levels of the 76 kDa fusion protein in agreement with the fluorescence intensity levels. We chose the clone with the highest fluorescence intensity for further experiments.

Generation of DYNLL1-mCherry and DYNLL2-mCherry expression plasmids

Dynll1 and Dynll2 CDC was amplified from mouse testis cDNA using the following primers: Dynll1_fw and Dynll1_rv: AAAAGAATTCATGTGCGACCGGAAGGC, TTTTGGATCCTTACCAGATTTGAACAGAAGAATG; Dynll2_fw and Dynll2_rv: AAAAGAATTCATGTCTGACCGGAAGGCAG, TTTTGGATCCTTGCCCGACTTGAGAGGAG). The amplified cDNA cloned into the p- mCherry-N1 plasmid (Clontech, PT3974-5). Plasmids were sent to GATC/Eurofins (Cologne, Germany) for sequencing.

Transfection of cells

HEK cells were cultured in DMEM (Gibco) with 10% FBS and transfected at 80% confluency. Transfection was performed using 3µg of expression plasmid with FuGENE HD Transfection

© 2023. Published by The Company of Biologists under the terms of the Creative Commons Attribution License (https://creativecommons.org/licenses/by/4.0/).

Reagent (Promega, Madison, USA) according to the manufacturer's instructions. Cells were imaged 12 h later using a Leica DM5500 B microscope.

Lines 725-728:

Proteins from HEK cells were isolated utilizing the M-PER^{TM} Mammalian Protein Extraction Reagent (Thermo Fisher Scientific) supplemented with Halt Protease Inhibitor Single-Use Cocktail EDTA-Free (Thermo Fisher Scientific) according to the manufacturers' instructions.
Lines 743-746:
GFP pull-down assay was performed using GFP nanobody-coupled magnetic beads (GFP- Trap Magnetic Particles Kit, Chromotek GmbH, Munich, Germany) according to the manufacturer's instructions. After IP, the bound proteins were eluted in Laemmli buffer and analyzed.

We have shown ACTL7B-DYNLL1/2 interaction both in whole testis protein extractions and *in situ*. Further we showed aberrant localization of DYNLL1/2 in presence/ absence of ACTL7B. The protein complex formed seems not to be denatured by the methods used.
Since same results were gathered *ex vivo* and *in situ*, as well as by Mass spec identification, we believe the interaction of ACTL7B and DYNLL1/2 to be sufficiently proven. Enrichment of the protein complex by antibody coupled beads may explain, why the band of higher molecular weight has not been identified in the n.b. fraction.

3. Relevant to the conclusion that sub-cellular localizations of DYNLL1/2 were changed in the absence of ACTL7B, the immunostaining showing in Fig. 6E-F seem different from staining shown in Fig. S6 and S7, in which wild type cells also contain granular structures in the cytoplasm at stage VII, the mutants only contained less. Is this due to the cells presented in Fig.6E-F are at different stages? Also, in lines 233-237, could co-immunostaining and confocal microscopy of ACTL7B and DYNLL1/2 be performed to see the sub-cellular distribution of them in wild type mice. Is the localization change a cause or a consequence of defected spermatids due to Actl7b deletion?

Authors reply: We agree that co-immunostaining of ACTL7B and DYNLL1 and DYNLL2 would add valuable information to this study. Co-immunostaining of ACTL7B and DYNLL1 or DYNLL2, however, could not be performed, since all three antibodies were raised in rabbit. Foci of DYNLL1 and DYNLL2 were rarely seen in WT or HET, but in increased numbers in KO. Vacuoles in the staining were only encountered in KO tubules. We consulted an expert, who looked again at our staging of tubules. He confirmed that these are correctly staged and in similar stages. Not in any stage did we encounter more foci in WT or HET tubules. The pictures presented in 6E-F are actually cut-outs from the overviews shown in Fig. S6 and S7 (now S11 and S12), and are therefore the same stages. This was now additionally indicated in the legend of Fig.6
Addition made to Figure legend (see lines: 1168-1169):
Note, that images shown in (E) and (F) are cut-outs from the overviews displayed in Fig. S11 and S12.
Changed localization of DYNLL1 and DYNNL2 in absence/presence of ACTL7B has now been additionally shown in HEK cells *in vitro* (see: Fig. 7D, S13 and S14). We believe that this is sufficient to show that ACTL7B influences DYNLL1 and DYNLL2 immunolocalization.
Addition made to Results part (lines: 297-305):
In HEK$^{Actl7b\text{-}eGFP}$cells, ACTL7B localized mainly to the cytoplasm and DYNLL1 and DYNLL2 were localized throughout the whole cytoplasm and the staining appeared fibrous (Fig. 7E-F, Fig. S13). In comparison, DYNLL1 and DYNLL2 localized mainly in the nucleus in WT HEK cells and only weakly in the cytoplasm. Further the staining looked much more even in WT HEK cells. Next, we transfected HEK cells with an expression plasmid containing the murine DYNLL1 or DYNLL2 fused to mCherry. Here, a similar staining pattern can be seen (Fig. S14). This supports the notion that ACTL7B interacts with and controls DYNLL1 and DYNLL2 localization in the cell.
Addition made to Discussion part (lines: 439-440):
Further, we show that expression of *Actl7b* in HEK cells alters DYNLL1 and DYNLL2 localization in the cell.

4. The link between Actin like proteins and microtubule through dynein light chain protein is very interesting, however, the comparison of F/G-actin, including the immunostaining shown in Fig. S8, as well as Ezrin changes shown in Fig S10 are of low resolution. Better confocal microscopic images at higher magnifications and quantitative data may be needed.

© 2023. Published by The Company of Biologists under the terms of the Creative Commons Attribution License (https://creativecommons.org/licenses/by/4.0/).

Authors reply: Thank you for this suggestion. The stainings for actin and ezrin have been repeated and imaged on a confocal microscope. The differences in staining are clearer now. The F/G-actin ratio was intended to serve as quantitative method, since a reduction or increase of F-actin would be reflected by a changed ratio. The newly imaged actin-staining clearly shows aberrant actin distribution (see Fig. S15)
The increase in ezrin level has been shown via mass spec (Table S1, Supplementary Material 2). Additionally, in IHC ezrin is detected only around tubuli in WT and HET, but additionally inside the tubuli of KO males. It is mainly seen at the borders of vacuolations in the epithelial tissue (See: Fig. S18C). We do not think that an additional method is needed to confirm the increase in ezrin levels.

Minor concerns:

1. Lines 160-161, Fig. 4A: What the “later stages” referring to? What are the upper and lower panels?

Authors reply: Figure 4 has been completely changed in the course of this Revision. PAS pictures have been replaced by images of PNA stained sections to evaluate acrosomal structures in more detail (see Fig. 4). See lines 197-201:
Since, ACTL7B is first present in round spermatids, effects were expected to manifest from round spermatid stage onwards. In comparison to *Actl7b+/-* and *Actl7b+/+*, in *Actl7b-/-* testis, acrosomal structures are less frequent and disorganized after spermatids start to elongate (Fig. 4A, Fig. S4A-B). In Golgi and Cap phase *Actl7b-/-* acrosomal structures appear normal, abnormalities become apparent in Maturation phase (Fig. 4A).
The following section has been added to the method section (lines 596-600):
Peanut agglutinin (PNA)-fluorescein isothiocyanite (FITC) Alexa 615 Fluor 488 conjugate (Molecular Probes, Invitrogen, Waltham, USA) was used on deparaffinized sections who were fixed using Bouin . After permeabilization with 0.1% Triton-X 100 for 5 min at RT, slides were blocked for 1 h with 1% BSA and incubated for 1 h with PNA (1:200). Slides were mounted with or ROTI Mount FluorCare DAPI (Carl Roth).

2. Lines 174-175, Fig. 5A: how is the “eviction of cytoplasm” different from wild type? The genotypes of mice used should be indicated on the graphs for better readability. A control image (wild type) may be included for data shown in Fig. 5C-F.

In this figure and Fig. 3B-C and Fig. S4, what are the dark granules that look like gold particles used for immuno-staining?

Authors reply: Thank you for this comment. We realized that we need to be clearer at this point of the manuscript. We added the genotypes to Fig. 5 and added a staining against cleaved protamine 2 to discuss the eviction of cytoplasm. See lines 218-221:
Staining against cP2 (to visualize residual cytoplasm) on caput epididymal sections showed, that large amounts of cP2 are retained in the cytoplasm of immature germ cells in *Actl7b-/-* mice (Fig. 5C). This again suggests defects in the eviction of cytoplasm.
We did not add an additional WT image for Fig. 5C-F (now: Fig.5D-E), since WT spermatid development is depicted in Fig. S7 and Fig. S8.

The dark granules on the TEM pictures shown do not originate from amino gold stainings. As far as we are aware these are liposomes and degrading RNAs. We see these granules on all TEM images taken regardless of the genotype or mouse line analyzed.

3. Lines 184-186, the malformation of nuclei in the KO sperm is quite obvious, however, the conclusion of chromatin condensation defect may not be drawn from microscopic images (Fig. 4, Fig. S5), should immuno-staining of transition proteins or protamines reveal some differences?
Authors reply: Thank you for this suggestion. To have a closer look at chromatin remodeling in *Actl7b-/-* testis we added new confocal stainings of PRM2 (see Fig. 4C) and an additional supplementary Figure (Fig. S5). See lines 205-214:
Staining against PRM2 in the nuclei of elongating spermatids in *Actl7b-/-*, *Actl7b+/-* and *Actl7b+/+*

© 2023. Published by The Company of Biologists under the terms of the Creative Commons Attribution License (https://creativecommons.org/licenses/by/4.0/).

testis sections indicates that nuclear remodeling and chromatin condensation is initiated (Fig. 4C). Transition proteins are loaded onto the DNA and later protamines are detected in the nuclei of spermatids (Fig. S5A-B). Staining against the cleaved domain of PRM2 (cP2) showed that PRM2 localizes to the nucleus of developing spermatids. Finally, in WT mice remaining full-length unprocessed PRM2 is evicted to the residual bodies (Fig. S5C), as described (Arévalo et al., 2022). In contrast, in *Actl7b-/-* testis sections residual bodies seem enlarged and less clearly separated from spermatid nuclei. Seemingly, the eviction of cytoplasm fails in large parts of *Actl7b-/-* spermatids and cP2 filled cytoplasm is retained

Additions were made to the method section (lines 570-595):

Immunohistochemistry (IHC)/ Immunofluorescence (IF)

Tissues were fixed in Bouin´s solution (4°C, overnight) processed in paraffin and 3 µm sections were generated utilizing a microtome (Microm CP60). Heat mediated antigen retrieval was performed at pH6 or pH9 (pH6: rabbit monoclonal (clone SD08-04) anti- DYNLL1 (Invitrogen, SD08-04, 1:1500), rabbit polyclonal anti-DYNLL2 (Proteintech, 16811- 1-AP, 1:1500), rabbit polyclonal anti-ACTL7B (Proteintech, 13537-1-AP, 1:750), mouse anti- PRM2 (Briar Patch Biosciences, Hup2B, 1:200), rabbit polyclonal anti-ODF2 (Proteintech, 12058-1-AP, 1:500), monoclonal (clone 3C12) mouse anti-Erzin (Santa Cruz, sc-58758, 1:100), rabbit polyclonal anti-TNP1 (Abcam, ab73135, 1:1000), rabbit anti-cP2 (custom antibody, Davids Biotechnologie GmbH, Regensburg, Germany, 1:500, (Arévalo et al., 2022)); pH9: rabbit anti-ACTIN monoclonal (clone EPR16769) (Abcam, ab179467, 1:500), rabbit polyclonal anti-PRND (Proteintech, 26947-1-AP, 1:500)). For slides stained with anti- ACTIN and anti-PRND an additional peroxidase blocking step was performed. Slides stained against PRM2, cP2 and TNP1 were additionally treated with decondensation buffer, as described previously (Schneider et al., 2020).

Sections stained with anti-DYNLL1, anti-DYNLL2, anti-TNP1, anti-cP2, anti-PRND, anti- ODF2 and anti-ACTIN were processed using the VectaFluor™ Horse Anti-Rabbit IgG, DyLight® 488 Antibody Kit (Vector Laboratories, Burlingame, CA, USA; DI-1788), sections stained against Ezrin were processed with the VectaFluor^{TM} Anti-Mouse IgG, DyLight® 594 Kit (Vector Laboratories, DI-2794), sections stained against PRM2 were processed with the VectaFluor™ Duet Immunofluorescence Double Labeling Kit, DyLight® 594 Anti-Rabbit, DyLight® 488 Anti-Mouse (Vector Laboratories; DK-8828) and sections stained with anti- ACTL7B were processed using the Vectastain□ ABC-AP Kit (Vector Laboratories; AK-5001) and ImmPACT□ Vector□ Red alkaline phosphatase substrate (Vector Laboratories; SK- 5105). For all stainings an extra 30 min blocking step with 5% BSA in PBS was performed. Fluorescent stainings were DAPI counterstained with ProLong™ Gold antifade reagent (Thermo Fisher Scientific) or ROTI Mount FluorCare DAPI (Carl Roth).

4. Please check if all labels (arrows, arrowheads) are placed correctly (i.e. Fig. 3, Fig. 4 and Fig. S4).

Authors reply: We re-checked the arrows and arrow heads in the indicated figures to assure that these are placed correctly.

5. Lines 206-208, only DYNLL1/2 is mentioned, what are the other proteins identified as positive hits in the Co-IP experiments?

Authors reply: We have added two sentences to this part of the manuscript to mention other proteins identified and to justify why we concentrated our analysis on the LC8 dynein light chains.

See lines 252-258:

Further, a published bead proteome from HeLa cells was used to filter out proteins which nonspecifically bind Dynabeads (Trinkle-Mulcahy et al., 2008). Those included various H2B histone variants. In the Co-IP using the anti-ACTL7B-coupled beads, we identified LC8 light chains, Dynein light chain 1 (DYNLL1) and its paralog dynein light chain 2 (DYNLL2) in a similar abundance as ACTL7B. Other proteins identified were ribonucleoproteins and ribosomal proteins, which were excluded from further analysis.

6. Lines 224-225, while WB shows that DYNLL1/2 maintained in KO mice, staining of them shown in Fig. S6 and S7 appeared less than those of wild type, is this due to the loading of proteins on gel or the number of cells used to extract lysates?

Authors reply: The western blots presented for DYNLL1 and DYNLL2 were used for quantification

© 2023. Published by The Company of Biologists under the terms of the Creative Commons Attribution License (https://creativecommons.org/licenses/by/4.0/).

including a standard loading control (alpha-tubulin) (see Fig. 7B). The IHC staining was intended to provide information on aberrant localization of the proteins.
We believe that the question we intend to answer with the IHC is not of higher or lower protein level, but altered localization. Since the DYNLL1/2 staining is less uniform in the KO (see Fig. S6 and S7, i.e. foci of more intense DYNLL1/2 staining indicative of clusters of accumulated DYNLL1/2), we do not expect the IHC images to give valuable information on protein quantity.

7. Lines 253-255, lines 584-586, please indicate the number of mice/testes used and number of experimental repeats performed for mass spectrometry.

Authors reply: As mentioned in the test, testes of 5 animals per genotype were used. All samples were processed in parallel and one experimental run. Hence, we have 5 biological replicates in one experiment. This why we do not mention experimental repeats.
See lines 312-314:
In order to analyze alterations in the testicular proteome in *Actl7b*-deficient mice, protein samples isolated from whole testes of each five *Actl7b-/-*, *Actl7b+/-* and *Aclt7b+/+* mice were used for mass spectrometric analysis (Supplementary material 2).
See lines 749-750:
Proteins from whole testis from five WT, Actl7b+/− and Actl7b−/− mice were isolated as described and used for mass spectrometric analysis.

8. The off-target effects of sgRNAs should be examined in the mouse line used, at least for the top potential candidates.

Authors reply: We are happy to comment on this: Through our many years of experience with gene editing using CRISPR/Cas, we have reached a high standard in terms of both personnel and equipment. Several CRISPR/Cas-based mouse models have already been published by us (Schneider *et al*. 2016, Schneider *et al*. 2020, Umer *et al*. 2021, Arévalo *et al*. 2021). Guides were tested in cell culture in advance.
See Methods section lines 524-528:
Single guide RNAs (sg1_ts: 5´-CACCCGGACACGGCGTGTCGCAT; sg1_bs: 5´-AAACCATGCGACACGCCGTGTCC; sg2_ts: 5´-CACCAATACGGAAGATCAAGGCG, sg2_bs: 5´-AAACGCGCCTTGATCTTCCGTAT) were designed using the Benchling CRISPR Guide RNA design tool (https://www.benchling.com/crispr/; ENSMUSG00000070980) and tested in ES cells as described (Schneider et al., 2016).
Additionally, we have now added the off-target scores of the guides.
See lines 531-535:
Potential off-targets of the single guide RNAs were analyzed using the “CRISPR-Cas9 guide RNA design checker” from IDT (https://eu.idtdna.com/site/order/designtool/index/CRISPR_SEQUENCE). The guides had off-target scores of 91/100 and 86/100, respectively. Only two potential off-targets on chromosome 4 were detected (for guide 2), both of which are localized in non-coding areas. We expect that if off-targets arise, they will arise on other, non-targeted chromosomes and be segregated by repeated backcrossing. Potential off-targets identified on chromosome 4 were localized in non-coding areas and are not expected to influence the phenotype of the mice analyzed.

9. Since the experiments were performed using fertilized eggs, “oocytes” should be changed to “fertilized eggs” or “zygotes” or “1-cell embryos”, e.g. in lines 455-458.

Authors reply: Thank you for this comment. We corrected our wording (see line: 111-113): We applied CRSIPR/Cas9-mediated gene editing in zygotes to generate *Actl7b*-deficient mice. Two guides were used targeting the intron-less coding sequence of *Actl7b* (Fig. 1A, arrowheads).

10. Lines 486, 489, what are the pH 6 and pH 9 for? Please indicate the antibodies as mouse monoclonal or rabbit polyclonal, etc. for the primary antibodies used in Materials and Methods.

Authors reply: The pH 6 and pH 9 describe the pH of the antigen retrieval buffers used for IHC. We added more detailed information on the antibodies used. See lines: 570-583 Tissues were fixed in Bouin´s solution (4°C, overnight) processed in paraffin and 3 µm sections were generated utilizing a microtome (Microm CP60). Heat mediated antigen retrieval was

© 2023. Published by The Company of Biologists under the terms of the Creative Commons Attribution License (https://creativecommons.org/licenses/by/4.0/).

performed at pH6 or pH9 (pH6: rabbit monoclonal (clone SD08-04) anti- DYNLL1 (Invitrogen, SD08-04, 1:1500), rabbit polyclonal anti-DYNLL2 (Proteintech, 16811- 1-AP, 1:1500), rabbit polyclonal anti-ACTL7B (Proteintech, 13537-1-AP, 1:750), mouse anti- PRM2 (Briar Patch Biosciences, Hup2B, 1:200), rabbit polyclonal anti-ODF2 (Proteintech, 12058-1-AP, 1:500), monoclonal (clone 3C12) mouse anti-Erzin (Santa Cruz, sc-58758, 1:100), rabbit polyclonal anti-TNP1 (Abcam, ab73135, 1:1000), rabbit anti-cP2 (custom antibody, Davids Biotechnologie GmbH, Regensburg, Germany, 1:500, (Arévalo et al., 2022)); pH9: rabbit anti-ACTIN monoclonal (clone EPR16769) (Abcam, ab179467, 1:500), rabbit polyclonal anti-PRND (Proteintech, 26947-1-AP, 1:500)). For slides stained with anti- ACTIN and anti-PRND an additional peroxidase blocking step was performed. Slides stained against PRM2, cP2 and TNP1 were additionally treated with decondensation buffer, as described previously (Schneider et al., 2020).

11. Fig. 2A, how the pregnancy rate was calculated should be described in the Materials and Methods. Please indicate the age of mice used for Fig. 2 (e.g. in the legend).
Authors reply: We added a sentence to the fertility assessment section in the methods descriptions. The age of mice used is also mentioned in the method section.
See lines 563-567:
Fertility assessment
Male mice, aged between 8-12 weeks, were mated 1:1/1:2 to C57BL/6J females and females were examined for presence of a vaginal plug daily. Plug positive females were separated and monitored for pregnancies and litter sizes. A minimum of five plugs per male were monitored. Pregnancy rate was determined by calculating the percentage of plugs resulting in life born litter.

***** Reviewer 2 Advance Summary and Potential Significance to Field:
In the present manuscript, Merges et al generated a knockout mouse model of Actl7b, an acrosome associated actin-like protein. They show homozygous loss of Actl7b results in male sterility, due to defects in spermiogenesis and the production of abnormal sperm. The strength of the paper is its proteomics analysis; however the histological (light and EM level) characterisation of spermatogenesis needs to be improved for it to be acceptable for publication.

In regard to novelty, the authors are unfortunate in their timing as a characterisation of male infertility in an Actl7b knockout mouse has just been published within the last month in the Biology of Reproduction (Clement et al 2023). However, the proteomics aspects of the current paper will still add valuable and novel information to field. The Clement et al 2023 paper, also focussed on a flagella defect not characterised herein, as opposed to the acrosome defects that the current paper suggests. As such, ensuring the acrosome phenotype is properly characterised is essential.

Reviewer 2 Comments for the Author:
[1.The]1.The authors will need to update the manuscript throughout to integrate the new insights from the recently published Actl7b paper in the Biology of Reproduction.

Authors reply: We want to thank the Reviewer to pointing out the publication by Clement et al. Indeed, we believe that integration of the findings by Clement et al. is not only mandatory, but adds to our study, since this study had a completely different focus.
The following statements were added to the manuscript.
Addition to Introduction (Lines 95-101):
A recent study by Clement *et al.* described the generation and analysis of *Actl7b*-null mice, (Clement et al., 2023). Male *Actl7b*-null mice are infertile and show severe oligoteratozoospermia with malformations of the sperm tails and heads.
Here, we generated an *Actl7b*-null allele using CRISPR/Cas9-mediated gene editing to investigate the role of ACTL7B in more detail. While *Aclt7b*+/- males were unaffected, *Actl7b*-/- males were infertile showing morphological sperm abnormalities as described previously (Clement et al., 2023).
Addition to Results (Lines 133-135):
Similar results have been described by Clement *et al.*, were homozygous male mice were

© 2023. Published by The Company of Biologists under the terms of the Creative Commons Attribution License (https://creativecommons.org/licenses/by/4.0/).

infertile and showed an approximately 20% reduction in testis weight compared to WT controls (Clement et al., 2023).
Addition to Results (Lines 166-178):
Clement *et al*. described the roundish cells being shed into the lumen of seminiferous tubules in their *Actl7b*-KO mouse model to be TUNEL-positive (Clement et al., 2023). Here, we detected vesicles filled with degrading spermatids in *Actl7b-/-* seminiferous tubules (Fig. 3B- C). These contained condensed nuclei, acrosomal and tail structures, mitochondria and granular material. This suggests, that Sertoli cells have engulfed and are degrading spermatids. However, TUNEL staining did not reveal significant differences (Fig. S2H). Of note, we found that a mammalian autophagy marker, microtubule-associated protein 1A/1B- light chain 3 (LC3) and cathepsin B (CTSB) levels significantly increased in *Act7b-/-* testis (Fig. 3D). Interestingly, deletion of CTSB in mice leads to inhibition of autophagy and promotion of apoptosis in murine testis, suggesting CTSB to be a regulator between autophagy and apoptosis during spermatid development (Wen et al., 2022). Hence, based on our data we favor autophagy to be the main cause of spermatid degradation.

Addition to Results (Lines 148-152):
We isolated sperm from the cauda epididymis from *Actl7b+/+*, *Actl7b+/-* and *Actl7b-/-* males via swim-out. Sperm count was severely reduced in *Actl7b-/-* with an average of 32,250 sperm from both cauda epididymides (Fig. S2B-C). This corresponds to a 1000-fold reduction in sperm number and differs from Clement *et al*. who showed an approximately 10- fold reduction (Clement et al., 2023).
Addition to Results (Lines 224-233):
Staining of epididymal sperm with PNA and Mitotracker showed no significant differences between *Actl7b+/-* and WT sperm (Fig. S6A). In contrast, *Actl7b-/-* epididymal sperm show mislocalization of acrosomal structures and mitochondria. In most *Actl7b-/-* sperm, PNA and Mitotracker signals were found along the whole tail and in the head region. Of note, Clement *et al*. reported mislocalization of mitochondria in their *Actl7b*-KO model (Clement et al., 2023). Clement *et al*. further describe mislocalization of flagellar proteins in KO sperm and multiple morphological malformations of flagellae. Therefore, we used IC staining against α- tubulin to analyze manchette formation in *Actl7b-/-* spermatids. Manchette appear irregular compared to WT and *Actl7b-/-* spermatid head shapes are abnormal (Fig. S6B).
Addition to Discussion (Lines 397-398):
We show that, in line with published data (Clement et al., 2023), loss of ACTL7B led to male infertility in mice due to absence of functional, mature sperm.
Addition to Discussion (Lines 411-420):
The phenotype of *Actl7b*-deficiency described here is highly similar to the one described by Clement *et al*., who used homologous recombination in ESC to generate and analyze an *Actlt7b*-KO mouse (Clement et al., 2023). Both models demonstrate severe reductions in epididymal sperm number, germ cell loss in testis, immature germ cell release from testis and multifaceted morphological sperm abnormalities. Of note, while we observed a 1000-fold reduction in sperm count (approximately 32,000,000 in WT vs. 32,000 in KO), Clement *et al*. reported this reduction to be 10-fold only. Both models were generated by deleting the entire Actl7b coding sequence (CDS), but the lines were established on C57Bl/6J by us and on C57BL/6N by Clement *et al*. (Clement et al., 2023). This might explain the phenotypic differences (Simon et al., 2013).

2. Please include a comment on overall health of mice, body weight data etc

Authors reply: Thank you for the comment. The health of our laboratory mice is checked regularly in form of a “Severity Assessment”. We have added the following statement to the Material and Method section (lines 549-552):
No significant differences in body weight were monitored for genetically altered mice compared to WT mice. Mice were assessed as newborn litter, litter at weaning and individual animals every three months starting at the age of 8 weeks. Nutritional status, posture, coat and orifices as well as behavior and reaction to handling were normal in *Actl7b*-deficient mice.

3. Lines 142 ‘ Late stage spermatids show abnormal morphologies’ – this is vague need to clarify what abnormalities you are referring.

Authors reply: We agree that this sentence was too vague and deleted it from the manuscript.

© 2023. Published by The Company of Biologists under the terms of the Creative Commons Attribution License (https://creativecommons.org/licenses/by/4.0/).

Since various new experiments were added to the study in the course of this revision process, we believe that sperm abnormalities are now described in sufficient detail.

[4.Fig]4.Fig 3 needs to be improved to include comparison control images, to ensure all differences reported in KO are valid and to illustrate the normal light and EM histology.

Authors reply: We have changed Fig. 3 and additionally included Fig. S3 to be more precise with our claims. WT control images can be seen in Fig. 2 and Fig. S3.

[5.In]5.In Fig 3B no lumen is evident suggesting it is not a perfect transverse section through a seminiferous tubule, as such the appearance of multiple stages of spermatids within one tubule needs to be interpreted with caution. It should be confirmed using PAS-stained testis sections and EM sections wherein the lumen is evident.

Authors reply: We have deleted the image 3B from the manuscript, since it seemed insufficient to show what we wanted to discuss. Instead we have included an additional supplementary figure (see Fig. S3).
The following description was added to the manuscript (lines 179-191):
Closer inspection of testicular sections revealed defects in synchronization of the epithelial cycle in *Actl7b-/-* mice. Cohorts of spermatids in stage IX start elongation but it appears they do not elongate in a synchronous manner (Fig.S3A). Spermatids do not condense properly and seem to localize too close to the basal lamina. Further, cohorts of round spermatids appear abnormal and seem disorganized at stage IX (Fig.S3B). In comparison, WT stage IX tubules appear more synchronized. Next, in stage X, morphologically abnormal elongating spermatids are detected, while in WT stage X tubules all elongating spermatids appear to be at the same stage and look normal (Fig. S3C). Finally, in stage VII seminiferous tubules of *Actl7b-/-* males all elongating spermatids were morphologically abnormal (Fig. S3D).
Compared to WT, *Actl7b-/-* elongating spermatids do not align properly at the lumen and are very few in number. Substantial numbers of round spermatids are formed in *Actl7b-/-* testis and spermatogenesis appears normal until this stage. As expected, meiosis was inconspicuous in *Actl7b-/-* testis and no increased numbers of apoptotic divisions were detected (Fig. S3E).

[6.The]6.The appearance of flagellar structures throughout the epithelium in 3B should also not be over interpreted. Flagella development initiates in early spermiogenesis. As such, round and elongating spermatid flagella are often projecting into the lumen from deep within the epithelium depending on the stage and the position of the spermatids. Noting there are some stages when spermatids are drawn close to the basement membrane (i.e., step 15 spermatids in stage V).

Authors reply: We agree that Fig. 3B has not been sufficient for the claims we have made. Fig. 3B was deleted from the manuscript. As shown in the answers to point 4 and 5, we have added a new supplementary figure (Fig. S3). Additionally, we have repeated the ODF2 staining and imaged the testis sections with a confocal microscope (see Fig. 4B, Fig. S4C).
The following statement was added to the results section (lines: 201-205):
A signal for ODF2 in the lumen of seminiferous tubules of *Actl7b-/-*, *Actl7b+/-* and *Actl7b+/+* testis sections indicated that sperm flagellar structures are formed (Fig. 4B, Fig S4C). These are, however, less frequent and more often found in clusters close to the basal membrane, again suggesting engulfment and degradation of spermatids (Fig. S4C).

7. More care needs to be taken with the formatting of many of the figures.
Please include seminiferous tubule stage and genotype on all figures. For many of the EM images, the authors should crop them appropriately i.e., to focus specifically on the structure/cell of interest.

Authors reply: We have added the stages of tubules depicted were we thought its necessary and re-checked with an expert in the field to ensure all staging was done correctly. The genotypes are given directly in the figure or the figure legends. Since various new stainings and new confocal images were added to the manuscript during the revision process, we think that all defects described are shown in sufficient detail. Further, we think that the TEM images used for staging the spermatids (Fig. S7 and S8) serve the reader best, when the whole cell is visible.

© 2023. Published by The Company of Biologists under the terms of the Creative Commons Attribution License (https://creativecommons.org/licenses/by/4.0/).

8. Lines 149-153 – the authors have only included sperm counts and the percentage of viable sperm for heterozygous and WT males. The counts for Actl7b-/- males should also be included (even if is very low).

Authors reply: We have conducted new experiments and added data to Fig. S2 and the following statement in the Results section (lines 146-159):
Differences between cauda epididymides of WT and *Actl7b-/-* males were already apparent upon inspecting the dissected organs (Fig. S2A). Cauda from *Actl7b-/-* males appeared smoother and less filled. We isolated sperm from the cauda epididymis from *Actl7b+/+*, *Actl7b+/-* and *Actl7b-/-* males via swim-out. Sperm count was severely reduced in *Actl7b-/-* with an average of 32,250 sperm from both cauda epididymides (Fig. S2B-C). This corresponds to a 1000-fold reduction in sperm number and differs from Clement *et al.* who showed an approximately 10-fold reduction (Clement et al., 2023). Sperm count was not significantly different between *Actl7b+/-* and WT males. *Actl7b+/-* sperm appear morphologically normal (Fig. S2D-E), viable (Fig. S2F) and motile (not shown). While *Actl7b-/-* males show a pathomorphological phenotype, loss of one allele of *Actl7b* seems to be phenotypically inconspicuous. In *Actl7b-/- mice*, daily sperm production (Fig. S2F) and the number of elongating spermatids per seminiferous tubule cross section (Fig. S2G) were significantly reduced, indicating that the reduction in spermatids originates at least partially from defective spermiogenesis in the testis.

[9.The]9.The authors should try to include data showing morphology of epididymal sperm. Swim out or black flush into a small volume (0.5 ml) for sperm smears for staining.

Authors reply: We have performed a swim-out of KO sperm. As described, only very few sperm can be isolated from the KO epididymis. We have included pictures of the swim-out (Fig. S2) and stained epididymal sperm with PNA and Mitored (Fig. S6). The following statement has been added to the manuscript:
Lines 224-233:
Staining of epididymal sperm with PNA and Mitotracker showed no significant differences between *Actl7b+/-* and WT sperm (Fig. S6A). In contrast, *Actl7b-/-* epididymal sperm show mislocalization of acrosomal structures and mitochondria. In most *Actl7b-/-* sperm, PNA and Mitotracker signals were found along the whole tail and in the head region. Of note, Clement *et al.* reported mislocalization of mitochondria in their *Actl7b*-KO model (Clement et al., 2023). Clement *et al.* further describe mislocalization of flagellar proteins in KO sperm and multiple morphological malformations of flagellae. Therefore, we used IC staining against α- tubulin to analyze manchette formation in *Actl7b-/-* spermatids. Manchette appear irregular compared to WT and *Actl7b-/-* spermatid head shapes are abnormal (Fig. S6B).

The following descriptions have been added to the methods section (lines 608-612):
PNA-Mito red staining was performed on PFA-fixed (4%, 20 min at RT) epididymal sperm. Sperm were incubated with 5 µg/ml Peanut agglutinin (PNA)-fluorescein isothiocyanite (FITC) Alexa 615 Fluor 488 conjugate (Molecular Probes) and 20 nm MitoTracker Red CMXRos (Cell Signaling, #9082) for 45 min at RT, washed in PBS and smeared on slides. Slides were mounted with or ROTI Mount FluorCare DAPI (Carl Roth).

[10.The]10.The reduction in testis weight suggests a loss of germ cells in spermatogonia and/or spermatocytes, this is because spermatids, particularly in final half of spermiogenesis, contribute minimally to the overall weight of the testis. This would also be consistent with the authors observations of vacuoles near the basement membrane The authors should assess whether spermatogonia or meiosis is abnormal. Indicative of abnormal meiosis a double nucleated spermatids is evident in Fig 5E.

Authors reply: Double nucleated spermatids were rarely detected in KO testis section and no prominent picture in the KO compared to WT and HET samples. Nevertheless, we consulted an expert and took a closer look at meiosis by specifically looking through images of stage I tubuli. Representative images can be seen in Fig. S3.
The following sentence was added to the manuscript (lines: 190-191):
As expected, meiosis was inconspicuous in *Actl7b-/-* testis and no increased numbers of apoptotic divisions were detected (Fig. S3E).

© 2023. Published by The Company of Biologists under the terms of the Creative Commons Attribution License (https://creativecommons.org/licenses/by/4.0/).

11.Line 163 – 'generally, less spermatids develop in Actl7b-/- testis.' This statement should be supported by a quantitative assessment of testis daily sperm production (which is distinct from epididymal sperm content)

Authors reply: Thank you very much for this suggestion. We took a closer look at sperm production and this added valuable data to the manuscript. The following section was added to the manuscript (lines: 156-159):
In *Actl7b-/- mice*, daily sperm production (Fig. S2F) and the number of elongating spermatids per seminiferous tubule cross section (Fig. S2G) were significantly reduced, indicating that the reduction in spermatids originates at least partially from defective spermiogenesis in the testis.

The following section was added to the methods section (lines 664-670):
Testicular daily sperm production
Daily sperm production was determined as described by Juma *et al.* (Juma et al., 2017) with modifications. In brief, after removal of the tunica albuginea, testes were homogenized in 400 µL DSP buffer (0.15 M NaCl, 0.1 M NaN3 and 0.05% Triton-X 100 in water). WT and heterozygous samples were adjusted to 4 ml, KO samples to 2 ml final volume using DSP buffer. Elongating spermatids were counted using a Neubauer counting chamber, the result was divided by 4.84.

[12.The]12.The characterisation of the acrosome phenotype is an integral part of this paper and needs to be improved.
[a.The]a.The power of the images in 4A is insufficient for a proper assessment of acrosome morphology and PAS-positive vesicles. These should be taken at 100x and Golgi phase should also be assessed. The authors should quantify the number of spermatids with abnormalities at the elongated spermatid stage.

Authors reply: We have included new confocal images of PNA stained testis sections (Fig. 4A, Fig. S4A-B). Acrosome biogenesis appears normal at Golgi phase. Like other defects that we see in KO mice, defects in acrosome biogenesis, appear later in spermiogenesis. This can also be seen in Fig. S7 and Fig. S8. Since KO sperm show defects in all structures (manchette, flagella and acrosome), we do not think that focusing even more on the acrosome is necessary. Sufficient descriptions of secondary effects have been made.
The following statement has been added (lines: 197-201):
Since, ACTL7B is first present in round spermatids, effects were expected to manifest from round spermatid stage onwards. In comparison to *Actl7b+/-* and *Actl7b+/+*, in *Actl7b-/-* testis, acrosomal structures are less frequent and disorganized after spermatids start to elongate (Fig. 4A, Fig. S4A-B). In Golgi and Cap phase *Actl7b-/-* acrosomal structures appear normal, abnormalities become apparent in Maturation phase (Fig. 4A).
The following statement has been added to the method section (lines: 596-600):
Peanut agglutinin (PNA)-fluorescein isothiocyanite (FITC) Alexa 615 Fluor 488 conjugate (Molecular Probes, Invitrogen, Waltham, USA) was used on deparaffinized sections who were fixed using Bouin. After permeabilization with 0.1% Triton-X 100 for 5 min at RT, slides were blocked for 1 h with 1% BSA and incubated for 1 h with PNA (1:200). Slides were mounted with or ROTI Mount FluorCare DAPI (Carl Roth).

[b.Fig]b.Fig S3, why is no step 3 KO presented? Higher magnification insets focussing just on the acrosome. Golgi and acroplaxome are needed.
Authors reply: We excluded the step 3 WT image, since we did not find an appropriate KO image in the images we took. Again, we think that we clearly showed that all defects seen start after step 9 of spermiogenesis and it is not necessary to look at Golgi stage in more detail. The added PNA images should be sufficient to make this point.

[c.Fig]c.Fig S4, again higher magnification insets focussing just on the acrosome and acroplaxome are needed, and step 13-14 ko panel is missing. Many of the elongating spermatids are incorrectly staged. The 'step 9' WT and the two far right 'step 9' Actl7b-/- spermatids are is at least step 10 or 11 based on their shape. The two far right two step '12' Actl7b-/- spermatids are at least step 13, based on their shape and the manchette position.

© 2023. Published by The Company of Biologists under the terms of the Creative Commons Attribution License (https://creativecommons.org/licenses/by/4.0/).

Authors reply: We still do not agree that we are dealing with a sole acrosome phenotype and also at no point make that claim throughout the manuscript. The defects seen in the acrosome could, like all other defects, be secondary effects due to disturbed cytoskeletal movements and sperm degradation. Focusing even more on acrosome biogenesis will, in our opinion, not add additional value to the study.
The staging in Fig. S4 (now Fig. S8) has been corrected.

13. Line '178-179' 'the general architecture of elongating spermatids appears disrupted' - please be more specific in your description

Authors reply: We have rephrased the sentence (lines 221-224):
In later developmental stages of *Actl7b-/-* spermatids in the testis, sperm membranes and acrosomal structures become detached (Fig. 5D-E). Part of the condensed nuclei show inclusions (Fig. 5F) and the overall organization of elongating spermatids appears disorganized (Fig. 5G). Sperm specific structures fail to assemble correctly.

14. Lines 181-182 'a subset of spermatids lacks the manchette, several spermatids show partly detached ectoplasmic F-Actin bundles'. This is not clear in the presented images. Higher magnification is needed. The manchette phenotypes should be confirmed with IHC of tubulin on testis sections.

Authors reply: The sentence has been deleted and the images of alpha-tubulin stained manchette structures have been added to the manuscript. Here we focus on those spermatids isolated from testis that showed manchette structures (Fig. S6B), since absence of manchette structures are most likely caused by spermiogenic arrest or spermatid degradation.
The following statement has been added to the manuscript (lines: 228-233):
Of note, Clement *et al.* reported mislocalization of mitochondria in their *Actl7b*-KO model (Clement et al., 2023). Clement *et al.* further describe mislocalization of flagellar proteins in KO sperm and multiple morphological malformations of flagellae. Therefore, we used IC staining against α-tubulin to analyze manchette formation in *Actl7b-/-* spermatids. Manchette appear irregular compared to WT and *Actl7b-/-* spermatid head shapes are abnormal (Fig. S6B).
[15.Fig]15.Fig 5A, please include appropriate control comparison images.

Authors reply: Fig. 5B is the control image to Fig. 5A. Fig. 5B is a WT image. We added labels of the genotype in the figure to be clearer.

[16.The]16.The characterisation of flagella defects if included needs to be improved. In Lines 187-188 the authors state 'the proximal centriole locates to the posterior region of the nucleus to form the basal body, but often the extension of the axoneme fails in Actl7b-/- spermatids. In later stage spermatids the flagellum is absent. However, cross- sections of properly formed flagella can be found detached from spermatids'

These observations seem to rely solely on EM, given that the plane of section in EM can sometimes mean that HTCA is not observed the authors should use another method to confirm this (e.g. epididymal sperm morphology, or spermatid dry downs staining for the axoneme). The authors should give an indication of the frequency of these phenotypes. As highlighted in the other comments, for the EM higher magnification images should be included to allow ultrastructure of the sperm tail to be more readily assessed by readers.

Authors reply: We believe that abnormalities in flagella structures are just one of the many defects *Actl7b-/-* sperm show. Additionally, defects in *Actl7b*-KO spermatids have been analyzed in detail by Clement et al. To go into more detail, we now include Mitored stainings of sperm, validating the defects described by Clement et al.
The following statement has been added to the manuscript (lines: 224-231):
Staining of epididymal sperm with PNA and Mitotracker showed no significant differences between *Actl7b+/-* and WT sperm (Fig. S6A). In contrast, *Actl7b-/-* epididymal sperm show mislocalization of acrosomal structures and mitochondria. In most *Actl7b-/-* sperm, PNA and Mitotracker signals were found along the whole tail and in the head region. Of note, Clement *et al.* reported mislocalization of mitochondria in their *Actl7b*-KO model (Clement et al., 2023). Clement *et al.*

© 2023. Published by The Company of Biologists under the terms of the Creative Commons Attribution License (https://creativecommons.org/licenses/by/4.0/).

further describe mislocalization of flagellar proteins in KO sperm and multiple morphological malformations of flagellae.
The following descriptions have been added to the methods section (lines 608-612):
PNA-Mito red staining was performed on PFA-fixed (4%, 20 min at RT) epididymal sperm. Sperm were incubated with 5 µg/ml Peanut agglutinin (PNA)-fluorescein isothiocyanite (FITC) Alexa 615 Fluor 488 conjugate (Molecular Probes) and 20 nm MitoTracker Red CMXRos (Cell Signaling, #9082) for 45 min at RT, washed in PBS and smeared on slides. Slides were mounted with or ROTI Mount FluorCare DAPI (Carl Roth).

[17.For]17.For the MS, why was KO testis not used as the control?

Authors reply: Thank you for this suggestion. The KO testis would have been a nice additional control indeed, however the beads only control gave us the chance to filter out non-specifically bound proteins

18.Lines 265 -266, might the increase in Sertoli cell proteins just be a function of the decreased amount of germ cells in the epithelium i.e., resulting in a higher ration of Sertoli cells to germ cells?

Authors reply: This is a very good comment; however, we believe that with the staining against ezrin (Fig. S18) we were able to validate the results gathered with mass spec. The same is true for other proteins validated (CTSB, Fig. 3D; PRND, Fig. S17)

[19.As]19.As a general comment the authors need to be careful in the description of their results, there is a tendency to make sweeping/vague statements. Need to be more precise in you wording, citing specific arrows on specific panels of figures in the in text description would help.

Authors reply: We hope that the additional stainings, experiments and changes to the text helped to make our claims clearer.

***** Reviewer 3 Advance Summary and Potential Significance to Field:
This is an exciting paper examining the role of a testis specific Actin-related proteins (Arp), ACT7LB, a highly conserved protein in primates and rodents in spermiogenesis. ACTL7B is specifically expressed in round and elongating spermatids, and the Actl7b-KO mice are infertile and display defects in sperm formation, cytoskeleton organization, and chromatin condensation, leading to spermatid arrest or loss at various stages of the seminiferous tubule cycle. To better understand the role of ACT7LB the authors performed IP-MS and find that ACT7LB interacts with dynein protein, which implicate ACT7LB and Dynein's in the global sperm remodeling process. This is an important body work, but additional experiments, clarifications and validations are needed to strengthen the work. Please see comments below:

1) It seems that prm2 in the mutant is cytoplasmic and not nuclear? Is that correct?

Authors reply: Thank you for sharing this observation with us. As mentioned in the answers to the comments of reviewer one, we added new PRM2 stainings and a new supplementary figure to take a closer look at nuclear remodeling in Actl7b-/- mice.
Please see Fig. 4C and supplementary Figure S5.
The following addition to the text has been made (lines 205-214):
Staining against PRM2 in the nuclei of elongating spermatids in *Actl7b-/-*, *Actl7b+/-* and *Actl7b+/+* testis sections indicates that nuclear remodeling and chromatin condensation is initiated (Fig. 4C). Transition proteins are loaded onto the DNA and later protamines are detected in the nuclei of spermatids (Fig. S5A-B). Staining against the cleaved domain of PRM2 (cP2) showed that PRM2 localizes to the nucleus of developing spermatids. Finally, in WT mice remaining full-length unprocessed PRM2 is evicted to the residual bodies (Fig. S5C), as described (Arévalo et al., 2022). In contrast, in *Actl7b-/-* testis sections residual bodies seem enlarged and less clearly separated from spermatid nuclei. Seemingly, the eviction of cytoplasm fails in large parts of *Actl7b-/-* spermatids and cP2 filled cytoplasm is retained

2) The EM images in 4C seem that the Actl7b-KO spermatids more dense – more contrast. Is this an imaging staining artifact or an indication of abnormal packaging?

© 2023. Published by The Company of Biologists under the terms of the Creative Commons Attribution License (https://creativecommons.org/licenses/by/4.0/).

Authors reply: The difference in density was an imaging artefact. To take a closer look at chromatin packaging we added new confocal stainings of PRM2 (see Fig. 4C) and an additional supplementary Figure (Fig. S5).

3) In figure 5 including reference panels for WT and also using similar magnification for WT/Mutant cell comparisons is necessary to make informed decisions by reviewer.

Authors reply: Thank you for this comment. We realized that we need to be clearer at this point of the manuscript. We added the genotypes to Fig. 5.

We did not add an additional WT image for Fig. 5C-F (now: Fig.5D-E), since WT spermatid development is depicted in Fig. S7 and Fig. S8.

4) In figure 6 – why do the eluate bands run much higher than the input? The bands are not as convincing.

Authors reply: To validate ACTL7B-DYNLL1/2 interaction, we generated a stable *Actl7b- eGFP* expressing HEK cell line. Staining of the endogenous human DYNLL1 and DYNLL2 showed, that both light chains show a different immunolocalization when ACTL7B is present compared to WT HEK cells (see Fig. 7E, Fig. S13). This was also seen when transfecting the cells transiently with murine DYNLL1 or DYNLL2 fused to mCherry (see Fig. S14).
Additionally, we were able to pull down DYNLL2 using GFP trap beads followed by western blot (see Fig. 6C). The same higher molecular weight complex was identified as seen for whole testis extractions.

<u>Additions made to Results part:</u>
<u>Lines 273-281:</u>

Next, we generated HEK cells stably expressing *Actl7b* fused to *eGFP* (HEK$^{Actl7b\text{-}eGFP}$) and performed a pull-down with GFP nanobody-coupled beads on whole protein extracts (Fig. 6C, Fig. S10). Protein extracts from WT HEK cells were used as control. We identified ACTL7B via Western blot both in the input and the IP eluate. ACTL7B was, as expected, not identified in the WT cells. Western blot using the DYNLL2 antibody identified multiple bands in the input from both cell lines, corresponding to the size of DYNLL2 alone and potential protein complexes of larger size. In the IP eluate from HEK$^{Actl7b\text{-}eGFP}$ cells a band positive for DYNLL2 was identified at the size of around 60 kDa, suggesting that DYNLL2 is bound to ACTL7B.

<u>Lines 297-305:</u>

In HEK$^{Actl7b\text{-}eGFP}$cells, ACTL7B localized mainly to the cytoplasm and DYNLL1 and DYNLL2 were localized throughout the whole cytoplasm and the staining appeared fibrous (Fig. 7E-F, Fig. S13). In comparison, DYNLL1 and DYNLL2 localized mainly in the nucleus in WT HEK cells and only weakly in the cytoplasm. Further the staining looked much more even in WT HEK cells. Next, we transfected HEK cells with an expression plasmid containing the murine DYNLL1 or DYNLL2 fused to mCherry. Here, a similar staining pattern can be seen (Fig. S14). This supports the notion that ACTL7B interacts with and controls DYNLL1 and DYNLL2 localization in the cell.

<u>Additions made to Discussion part (lines: 439-440):</u>

Further, we show that expression of Actl7b in HEK cells alters DYNLL1 and DYNLL2 localization in the cell.

<u>Additions made to Methods section:</u>
<u>Lines 689-719:</u>

<u>Generation of HEK$^{Actl7b\text{-}eGFP}$cells</u>

Actl7b CDS was amplified using the Q5 high fidelity thermostable DNA polymerase (NEB) from mouse testis cDNA using the following primers (GFP-Actl7b_fw and GFP-Actl7b_rv: ctcgagctcaagcttcgatggcgacaaagaacag, ggtaccgtcgactgcagttagcacttgctgtagatgg) and inserted into the pEGFP-C1 vector (Clontech) digested with EcoRI (NEB) using the NEBuilder HiFi DNA Assembly kit (NEB) according to the manufacturer's instructions. The pEGFP-C1-Actl7b plasmid was then linearized with ApaLI (NEB) and transfected into HEK293 cells using Lipofectamine^{TM} 2000 (ThermoFisher Scientific). Next, cells were selected for 3 weeks with 1 mg/ml geneticin^{TM} (G418 sulfate, Gibco), trypsinized,

© 2023. Published by The Company of Biologists under the terms of the Creative Commons Attribution License (https://creativecommons.org/licenses/by/4.0/).

diluted to 0.5 cells/100 µl and plated 100 µl/well on a 96-well plate. After 6 weeks five clones with different degrees of fluorescence were selected, expanded in 0.8 mg/ml geneticin^{TM} and cryopreserved. Protein lysates of the five clones were tested by western blotting with polyclonal anti-ACTL7B (Invitrogen, PA5-113560) and anti-GFP (kind gift from Prof. Dr. Oliver Gruss, Institute of Genetics, Bonn University). All clones showed different expression levels of the 76 kDa fusion protein in agreement with the fluorescence intensity levels. We chose the clone with the highest fluorescence intensity for further experiments.

Generation of DYNLL1-mCherry and DYNLL2-mCherry expression plasmids
Dynll1 and *Dynll2* CDC was amplified from mouse testis cDNA using the following primers: Dynll1_fw and Dynll1_rv: AAAAGAATTCATGTGCGACCGGAAGGC, TTTTGGATCCTTACCAGATTTGAACAGAAGAATG; Dynll2_fw and Dynll2_rv: AAAAGAATTCATGTCTGACCGGAAGGCAG, TTTTGGATCCTTGCCCGACTTGAGAGGAG). The amplified cDNA cloned into the p- mCherry-N1 plasmid (Clontech, PT3974-5). Plasmids were sent to GATC/Eurofins (Cologne, Germany) for sequencing.

Transfection of cells
HEK cells were cultured in DMEM (Gibco) with 10% FBS and transfected at 80% confluency. Transfection was performed using 3µg of expression plasmid with FuGENE HD Transfection Reagent (Promega, Madison, USA) according to the manufacturer's instructions. Cells were imaged 12 h later using a Leica DM5500 B microscope.

Lines 725-728:

Proteins from HEK cells were isolated utilizing the M-PER^{TM} Mammalian Protein Extraction Reagent (Thermo Fisher Scientific) supplemented with Halt Protease Inhibitor Single-Use Cocktail EDTA-Free (Thermo Fisher Scientific) according to the manufacturers' instructions.
Lines 743-746:
GFP pull-down assay was performed using GFP nanobody-coupled magnetic beads (GFP-Trap Magnetic Particles Kit, Chromotek GmbH, Munich, Germany) according to the manufacturer's instructions. After IP, the bound proteins were eluted in Laemmli buffer and analyzed.

We have shown ACTL7B-DYNLL1/2 interaction both in whole testis protein extractions and *in situ*. Further we showed aberrant localization of DYNLL1/2 in presence/ absence of ACTL7B. The protein complex formed seems not to be denatured by the methods used. Since same results were gathered *ex vivo* and *in situ*, as well as by Mass spec identification, we believe the interaction of ACTL7B and DYNLL1/2 to be sufficiently proven. Enrichment of the protein complex by antibody coupled beads may explain, why the band of higher molecular weight has not been identified in the n.b. fraction.

5) Is the reduction in figure 6 for ACTl7A and Hook1 just due to the loss of round and elongating spermatid populations. Given the proportion of different cell populations differ between the WT and mutant – wouldn't it be better to sort different populations from WT and mutant and run westerns on equivalent number of cells from each type? This will give a better representation of the changes in protein levels in respective population. This approach will work if these proteins are broadly expressed in spermatids etc… because then you won't have population loss as a confounding factor.

Authors reply: Thank you for this valuable comment. The reduction in Actl7a and Hook1 protein levels have discussed to be most likely due to reduction in the number of elongating spermatids in the first version of the manuscript. We excluded the western blots for Actl7a and Hook1 in the new version and included new experiments to describe the reduction in sperm number already in the testis.
Fig. S2 and the following statement in the Results section (lines 146-159):
Differences between cauda epididymides of WT and *Actl7b-/-* males were already apparent upon inspecting the dissected organs (Fig. S2A). Cauda from *Actl7b-/-* males appeared smoother and less filled. We isolated sperm from the cauda epididymis from

© 2023. Published by The Company of Biologists under the terms of the Creative Commons Attribution License (https://creativecommons.org/licenses/by/4.0/).

Actl7b+/+, *Actl7b+/-* and *Actl7b-/-* males via swim-out. Sperm count was severely reduced in *Actl7b-/-* with an average of 32,250 sperm from both cauda epididymides (Fig. S2B-C). This corresponds to a 1000-fold reduction in sperm number and differs from Clement *et al.* who showed an approximately 10-fold reduction (Clement et al., 2023). Sperm count was not significantly different between *Actl7b+/-* and WT males. *Actl7b+/-* sperm appear morphologically normal (Fig. S2D-E), viable (Fig. S2F) and motile (not shown). While *Actl7b-/-* males show a pathomorphological phenotype, loss of one allele of *Actl7b* seems to be phenotypically inconspicuous. In *Actl7b-/- mice,* daily sperm production (Fig. S2F) and the number of elongating spermatids per seminiferous tubule cross section (Fig. S2G) were significantly reduced, indicating that the reduction in spermatids originates at least partially from defective spermiogenesis in the testis.
We believe that sorting different cell populations goes beyond the scope of this manuscript and will not add valuable information to the study.

6) The file I downloaded had missing supplemental figures

Authors reply: We are very sorry for this inconvenience and hope that all supplementary figures will be available for inspection in the re-submitted version of the manuscript.

7) In Figure 6E and F – is the mislocalization of DynLL1/2 just because spermatids do not shed the cytoplasmic droplet? If you compare the mutant spermatids in KO to an earlier stage in WT, do you see the same pattern? If spermatids stop progressing the stage your analyzing in the mutant may not match exactly the WT?

Authors reply: Indeed, we could show that large portions of residual cytoplasm is evicted from seminiferous tubules and detectable in the epididymis.
We added a staining against cleaved protamine 2 to discuss the eviction of cytoplasm.
<u>See lines 218-221:</u>
Staining against cP2 (to visualize residual cytoplasm) on caput epididymal sections showed, that large amounts of cP2 are retained in the cytoplasm of immature germ cells in *Actl7b-/-* mice (Fig. 5C). This again suggests defects in the eviction of cytoplasm.
However, depict DYNLL1 and DYNLL2 stainings for different stages of the epithelial cycle in figures S11 and S12, clearly showing the difference in staining between WT and KO.

8) Validating proteomic differences on pure populations will circumvent the differences in cell populations in the wt, het and mutant testis.

Authors reply: This is a very good comment; however, we believe that with the staining against ezrin (Fig. S18) we were able to validate the results gathered with mass spec. The same is true for other proteins validated (CTSB, Fig. 3D; PRND, Fig. S17)

We do not believe that additional validation of the quantitative mass spec results are needed and that separation of cell populations would go beyond the scope of this study.

<u>Second decision letter</u>

MS ID#: DEVELOP/2023/201593

MS TITLE: Actl7b-deficiency leads to mislocalization of LC8 type dynein light chains and disruption of murine spermatogenesis

AUTHORS: Gina Esther Merges, Lena Arevalo, Andjela Kovacevic, Keerthika Lohanadan, Dirk G. de Rooij, Carla Simon, Melanie Jokwitz, Walter Witke, and Hubert Schorle

© 2023. Published by The Company of Biologists under the terms of the Creative Commons Attribution License (https://creativecommons.org/licenses/by/4.0/).

I have now received all the referees' reports on the above manuscript, and have reached a decision. The referees' comments are appended below, or you can access them online: please go to BenchPress and click on the 'Manuscripts with Decisions' queue in the Author Area.

As you will see, the referees in general appreciate your efforts to improve the manuscript, but still have some concerns and recommend a substantial revision of your manuscript before we can consider publication. If you are able to revise the manuscript along the lines suggested, which may involve further experiments, I will be happy receive a revised version of the manuscript. Your revised paper will be re-reviewed by one or more of the original referees, and acceptance of your manuscript will depend on your addressing satisfactorily the reviewers' major concerns. Please also note that Development will normally permit only one round of major revision. If it would be helpful, you are welcome to contact us to discuss your revision in greater detail. Please send us a point-by-point response indicating your plans for addressing the referee's comments, and we will look over this and provide further guidance.

Please attend to all of the reviewers' comments and ensure that you clearly highlight all changes made in the revised manuscript. Please avoid using 'Tracked changes' in Word files as these are lost in PDF conversion. I should be grateful if you would also provide a point-by-point response detailing how you have dealt with the points raised by the reviewers in the 'Response to Reviewers' box. If you do not agree with any of their criticisms or suggestions please explain clearly why this is so.

Reviewer 1

Advance summary and potential significance to field

The authors of this manuscript found that ACTIN like protein ACTL7B plays important roles during the elongation stage of spermiogenesis. Using gene targeting in mouse, they found that male Actl7b homozygous mutants are infertile, possibly due to the drastic defects in cellular morphogenesis of haploid spermatids, which carried malformed nuclei and flagella. Using biochemical and proteomics methods, they found that ACTL7B may form complexes with dynein light chain proteins DYNLL1 and DYNLL2, suggesting an interesting link between cytoskeletal networks for the coordinated morphogenesis of sperm. Mutant testis contained varied levels of numerous proteins comparing to those of wild type and heterozygous controls.
Their results potentially add an important clue to the understanding of how spermatogenic cells coordinate complexed morphological changes during spermiogenesis and what molecular players may be involved in this process.

Comments for the author

In this revision, the authors made improvements by including new experiments and re-structuring some of the figures and the main text. The quality of the images in figures have also been improved. The results presented much clearer on the phenotypes caused by Actl7b mutation during mouse spermiogenesis and indicated important roles ACTL7B plays that warrant for further studies. However, the manuscript has some drawbacks preventing it from presenting the main conclusions convincingly and providing a better ground for the understanding of potential mechanisms involved, thus, being acceptable for publication in Development in its current form.
One of the main concerns is the interaction between ACTL7B and DYNLL1/2. For example, in Figure 6A, the Co-IP efficiency of anti-ACTL7B appeared low as shown by the WBs using the three antibodies (for ACTL7B DYNLL1 and DYNLL2, respectively). Figure 6C shows a weak signal of 12-KD band (DYNLL2) but strong signal at the ~60 KD position in wild type HEK cells, suggesting that the specificity of anti-DYNLL2 should be verified. In the same vein, Figure S9A shows the second staining using anti-ACTL7B after anti-DYNLL2, both of which are rabbit polyclonal (so as Figure S9C). Figure S10's co-IP sample also showed that the bands recognized by anti-ACTL7B and anti-DYNLL2 may be at different molecular weights, although they are the same anti-ACTL7B co-IP samples (Left panels). Figure S10's anti-ACTL7B WB (upper panels) also showed protein bands around 50 KD, not the 76 KD GFP fusion proteins. The interaction between ACTL7B and DYNLL1/2 need to be verified by other methods, e.g. using tagged proteins co-expressed in cell lines or recombinant proteins expressed in bacteria for in vitro binding assays.
Other main concerns are:

© 2023. Published by The Company of Biologists under the terms of the Creative Commons Attribution License (https://creativecommons.org/licenses/by/4.0/).

1) Overstatements should be avoided when draw conclusions from the phenotypic analyses;
2) The rationales of various experiments should be presented logically and English should be checked and improved throughout the main text, including the Summary statement.
Some minor concerns:
1) Indicate PCR primers in the figure (Figure 1A) for genotyping;
2) Age of mice used should be indicated in the text or in figure legends, for example, for Figure 2 (adults), and for experiments analyzing apoptosis vs. autophagy (line 149) and the reduction of sperm count (lines 154-156). These phenotypical differences observed by the two groups may be discussed in Discussion, e.g. how different strains of mice could cause. Line 160, Figure S2F, how DSP calculated?
3) In Figure 3A, include a wild type control; Figure 3B-C, if it shows the vesicles containing degrading spermatids, the acrosomal structures are unlikely those indicated by arrowheads;
4) Figure S4 legend; lines 215-216 vs. Figure 5C legend;
5) Lines 220-223, Figure 5A-B, at TEM levels, it is hard to tell the cytoplasmic eviction and spermiation should indicate which structures could help to implicate these?
6) Figure S6A, MitoTracker red staining not clear along the whole tail in KO species, as described in the text line 233;
7) Figure S7, what the boxed regions show, not indicated in the legend; Figure S8, no boxed regions in the figure; instead of marking sub-cellular structures in the images with too many labels, it may be more helpful to indicate out the differences between wild type and KO species.

Reviewer 2

Advance summary and potential significance to field

The authors have made several improvements in line with the previous reviews however a number of areas still have not been adequately addressed for publication and a number of the conclusions are still not properly supported by the data.

Comments for the author

Major comments

Chromatin condensation defect: No chromatin condensation defects are apparent in Fig S8.

Meiosis being normal: the rationale to use stage I tubules to assess meiosis is unclear. Meiosis occurs over multiple stages with the meiotic divisions of meiosis I (metaphase/anaphase/telophase) and meiosis II occurring at stage XII, whereas prophase I spans stages I-XI. The only reason to assess stage I would be to observe if apoptotic metaphase/anaphase/telophase I and meiosis II cells stall into stage I, however that is not clear as written and stage XII should also be assessed to allow direct assessment of if the divisions are abnormal. A control image is also needed regardless for Fig S3E and a larger view of the stage I tubules as at present very few round spermatids can be seen.

Mislocalisation of mitochondria in knockout sperm tails: in describing Fig S6 the authors state that mitotracker signal was abnormally along the whole length of the tail in the knockout males. However, the mitotracker signals also appear along the whole tail of the WT and Het images presented and are comparable in that respect to the KO. The difference in the far right KO image wherein the mid-piece is clearly abnormal, however as the mitotracker signals overall appear non-specific ultrastructure analysis via TEM or SEM of sperm tails is needed to verify if this is due to abnormal mitochondrial loading.

Cytoplasm eviction defect: is not clear in Fig 5 A and B. The rational to use these images to show eviction of cytoplasm is unclear as these stages seem to be well before spermiation, or are you referring to the single spermiated sperm in the knockout lumen in the lumen? If yes to the latter arrows are needed and an control appropriate control showing a spermiated WT sperm with not retained cytoplasm is needed.

Formatting of figures and inclusion of control panels still needs attention:
1. Fig S3A panel is labelled as stage VI, in text is referred to as IX? Also a

© 2023. Published by The Company of Biologists under the terms of the Creative Commons Attribution License (https://creativecommons.org/licenses/by/4.0/).

control panel is needed for comparison to Fig S3A
2. Fig S3E as above control panel is needed for comparison
3. Fig 3 as detailed above stage matched control images are 3. Fig S4 figure legend – panel numbers need to be corrected in legend and tubule stage should be included on images are needed for Fig 3A and 3B/C
4. Fig 4B - panels are not labelled with respective genotypes

Minor comments

Sperm number - swim out is not a quantitative method. It biases against immotile sperm and also the number of sperm that vacate the epididymis can be influenced by the number of cuts made etc. Accurate quantification required homogenisation of the testis. That the swim out method was used both herein and in the Clements paper may contribute to the differences in results, in addition to the strain of the animals as the authors suggest.

Number of elongating spermatids per tubule cross section - please specify stage of seminiferous tubule used for this assessment and how many tubules assessed per mouse.

Reviewer 3

Advance summary and potential significance to field

This is an exciting paper examining the role of a testis specific Actin-related proteins (Arp), ACT7LB, a highly conserved protein in primates and rodents in spermiogenesis. ACTL7B is specifically expressed in round and elongating spermatids, and the Actl7b-KO mice are infertile and display defects in sperm formation cytoskeleton organization, and chromatin condensation, leading to spermatid arrest or loss at various stages of the seminiferous tubule cycle. To better understand the role of ACT7LB the authors performed IP-MS and find that ACT7LB interacts with dynein protein, which implicate ACT7LB and Dynein's in the global sperm remodeling process. This is an important body work for the field.

Comments for the author

The authors have done addressed many of the raised concerns. This is good for publication.

Second revision

Author response to reviewers' comments

MS ID#: DEVELOP/2023/201593
MS TITLE: Actl7b-deficiency leads to mislocalization of LC8 type dynein light chains and disruption of murine spermatogenesis
AUTHORS: Gina Esther Merges, Lena Arevalo, Andjela Kovacevic, Keerthika Lohanadan, Dirk G. de Rooij, Carla Simon, Melanie Jokwitz, Walter Witke, and Hubert Schorle

We like to thank the reviewers for their repeated input in our manuscript. Please find below a point by point reply to all comments.

Reviewer 1 Advance Summary and Potential Significance to Field:
The authors of this manuscript found that ACTIN like protein ACTL7B plays important roles during the elongation stage of spermiogenesis. Using gene targeting in mouse, they found that male Actl7b homozygous mutants are infertile, possibly due to the drastic defects in cellular morphogenesis of haploid spermatids, which carried malformed nuclei and flagella. Using biochemical and proteomics methods, they found that ACTL7B may form complexes

© 2023. Published by The Company of Biologists under the terms of the Creative Commons Attribution License (https://creativecommons.org/licenses/by/4.0/).

with dynein light chain proteins DYNLL1 and DYNLL2, suggesting an interesting link between cytoskeletal networks for the coordinated morphogenesis of sperm. Mutant testis contained varied levels of numerous proteins comparing to those of wild type and heterozygous controls.
Their results potentially add an important clue to the understanding of how spermatogenic cells coordinate complexed morphological changes during spermiogenesis and what molecular players may be involved in this process.

Reviewer 1 Comments for the Author:
In this revision, the authors made improvements by including new experiments and re-structuring some of the figures and the main text. The quality of the images in figures have also been improved. The results presented much clearer on the phenotypes caused by Actl7b mutation during mouse spermiogenesis and indicated important roles ACTL7B plays that warrant for further studies. However, the manuscript has some drawbacks preventing it from presenting the main conclusions convincingly and providing a better ground for the understanding of potential mechanisms involved, thus, being acceptable for publication in Development in its current form.
One of the main concerns is the interaction between ACTL7B and DYNLL1/2. For example, in Figure 6A, the Co-IP efficiency of anti-ACTL7B appeared low as shown by the WBs using the three antibodies (for ACTL7B, DYNLL1 and DYNLL2, respectively). Figure 6C shows a weak signal of 12-KD band (DYNLL2) but strong signal at the ~60 KD position in wild type HEK cells, suggesting that the specificity of anti-DYNLL2 should be verified. In the same vein, Figure S9A shows the second staining using anti-ACTL7B after anti-DYNLL2, both of which are rabbit polyclonal (so as Figure S9C). Figure S10's co-IP sample also showed that the bands recognized by anti-ACTL7B and anti-DYNLL2 may be at different molecular weights, although they are the same anti-ACTL7B co-IP samples (Left panels). Figure S10's anti-ACTL7B WB (upper panels) also showed protein bands around 50 KD, not the 76 KD GFP fusion proteins. The interaction between ACTL7B and DYNLL1/2 need to be verified by other methods, e.g. using tagged proteins co-expressed in cell lines or recombinant proteins expressed in bacteria for in vitro binding assays.

Authors reply: We did first identify the interaction between ACTL7B and DYNLL1/2 using Mass.-Spec. analyses. There the results are clear as the Mass.-Spec data clearly identify DYNLL1/2. This, is to our understanding, a highly precise method for protein identification. The Mass-Spec. were confirmed using CoIP (Fig. 6A) and reciprocal CoIP (Fig. 6B) followed by Western-Blot analyses. Further, we generated 293-HEK cells stably overexpressing ACTL7B and demonstrated that DYNLL chains are localized differently in these cells compared to control 293-HEK cells (Fig. S13). Finally, pulldown of ACTL7B in these HEK cells showed that ACTL7B binds to DYNLL2 in these cells (Fig. 6C). We agree that the Antibodies used (and the only ones available) are not the best in terms of overall performance. However, we would like to point out that DYNLL chains are known to form complexes with other proteins, which explains the multiple bands detected in our experiments (Fig. 6A. & 6C).
So, having used in sum five different approaches which probed this interaction from various sides, makes us confident in claiming that ACTL7B-DYNLL interact.
Further, we think, that we have already addressed reviewer 1's request: "The interaction between ACTL7B and DYNLL1/2 need to be verified by other methods, e.g. using tagged proteins co-expressed in cell lines" by tagging the ACTL7B with GFP and expressing it in 293HEK cells, which have endogenous DYNLL1/2 expression and performed WB and IHC experiments as shown in Fig. 7 E, F and Fig. S13. Further, we used the ACTL7B-GFP HEK-cells and transfected in DYNLL1-mCherry and DYNLL2-mCherry. These results, presented in Fig S14 B, C, and D confirm the results of the IHC presented in Fig. S13. So, in sum we would like to disagree with Reviewer 1 which requests more experiments aimed at this interaction.

Reviewer 1 Comment
Other main concerns are:
1) Overstatements should be avoided when draw conclusions from the phenotypic analyses;
Authors reply: We edited our statements and claims of several experiments as detailed below.
Reviewer 1 Comment

© 2023. Published by The Company of Biologists under the terms of the Creative Commons Attribution License (https://creativecommons.org/licenses/by/4.0/).

2) The rationales of various experiments should be presented logically and English should be checked and improved throughout the main text, including the Summary statement.

Authors reply: We tried to the best of our knowledge adhere to proper English language - the spell checker is set to US-English though, there might be some differences. Further, the summary statement was edited, it reads now (line 29 ff):

In this study, Actl7b-deficient mice were generated. Loss of Actl7b leads to spermatogenic arrest in mice. ACTL7B interacts with DYNLL1/DYNLL2 and contributes to dynamic change in cellular remodeling during spermiogenesis.

Reviewer 1
Comment
Some minor concerns:
1) Indicate PCR primers in the figure (Figure 1A) for genotyping;

Authors reply: We added purple arrowheads to indicate the location of the PCR-Primers used for genotyping. This was added to the legend (Line 1095):

Location of PCR-primers used for genotyping is indicated by purple arrowheads

In addition, we would like to emphasize that we had included information of the sequence of the primers as detailed in material and methods (line 546 ff):

Primers flanking the gene edited region (Actl7b_fwd: 5´- GGGACACAGGTTCCACTCAAC, Actl7b_rev: 5´- AGGTAGTTGGTGAGGTCGCA) were used to amplify both the WT and edited allele (Cycling conditions: 5 min 95°C; 35x (30 sec 95°C; 30 sec 60°C; 45 sec 72°C); 5 min 72°C). PCR products (WT allele: 607 bp, *Actl7b*-: 134 bp) were separated on agarose gels.

Reviewer 1 Comment
2) Age of mice used should be indicated in the text or in figure legends, for example, for Figure 2 (adults), and for experiments analyzing apoptosis vs. autophagy (line 149) and the reduction of sperm count (lines 154- 156).

Authors response: We added the following text (line 543):
All further experiments were performed with males aged 8-12 weeks unless stated otherwise

Reviewer 1 Comment
These phenotypical differences observed by the two groups may be discussed in Discussion, e.g. how different strains of mice could cause.

Authors comment: We think that we already included this topic in the discussion. Due to length restrictions, we unfortunately have not enough space to go into this topic/issue further.

Reviewer 1 Comment
Line 160, Figure S2F, how DSP calculated?
Authors response: The explanation of the method of daily sperm production (DSP) was added in the first round of the revision. We explain the method in line 655 ff. It reads:

<u>Testicular daily sperm production</u>
Daily sperm production was determined as described by Juma *et al.* (Juma et al., 2017) with modifications. In brief, after removal of the tunica albuginea, testes were homogenized in 400 µL DSP buffer (0.15 M NaCl, 0.1 M NaN_3 and 0.05% Triton-X 100 in water). WT and heterozygous samples were adjusted to 4 ml, KO samples to 2 ml final volume using DSP buffer. Elongating spermatids were counted using a Neubauer counting chamber, the result was divided by 4.84.

© 2023. Published by The Company of Biologists under the terms of the Creative Commons Attribution License (https://creativecommons.org/licenses/by/4.0/).

Reviewer 1 Comments:
3) In Figure 3A, include a wild type control; Figure 3B-C, if it shows the vesicles containing degrading spermatids, the acrosomal structures are unlikely those indicated by arrowheads;

Authors response: Wt control are added and shown in Fig. 3A. For Fig. 3B-C we did not mean to indicate the presence of “vesicles” but rather degrading spermatids, we corrected this issue in the Figure legend and text. The sentence reads now Line 168:

Here, we detected degrading spermatids in *Actl7b-/-* seminiferous tubules (Fig. 3B-C).

..and line 394:

Degrading spermatids seem to be eliminated by Sertoli cells and levels of autophagy marker proteins are increased in *Actl7b-/-* testes.

The legend reads now (line 1121):
Transmission electron micrographs of degrading spermatids detected in Actl7b-/- seminiferous tubules.

Reviewer 1 Comments:
4) Figure S4 legend; lines 215-216 vs. Figure 5C legend;
5) Lines 220-223, Figure 5A-B, at TEM levels, it is hard to tell the cytoplasmic eviction and spermiation, should indicate which structures could help to implicate these?

Authors response: We discussed our interpretation based on the reviewer’s comments. We agree that we might have overstated and overinterpreted the results. We amended the respective sections and watered the claim down and kept it on a descriptive level. It now reads (Line 210 ff):

Seemingly, in *Actl7b-/-* spermatids cytoplasm (labelled by cP2 staining) is retained. Transmission electron micrographs reveal that spermatids in *Actl7b-/-* testes show abnormal morphologies and excess cytoplasm (Fig. 5A). In comparison, in the lumen of *Actl7b+/+* tubules morphologically normal sperm line up to be spermiated (Fig. 5B). Staining against cP2 (to visualize cytoplasm) on caput epididymal sections produced a strong signal in immature germ cells in *Actl7b-/-* mice indicating the presence of cytoplasm (Fig. 5C).

Reviewer 1 Comment
6) Figure S6A, MitoTracker red staining not clear along the whole tail in KO species, as described in the text, line 233;

Authors comments: We discussed the reviewers concern. We agree that we might have overstated the finding and REMOVED the sentence formerly in line 224-226 in order to clarify this section.

~~“In most *Actl7b-/-* sperm, PNA and Mitotracker signals were found along the whole tail and in the head region.”~~

Reviewer 1 Comment
7) Figure S7, what the boxed regions show, not indicated in the legend;

Authors comments: This might be an error by the reviewer since the legend to Figure S7 indicated: “boxes: marginal ring region”
Reviewer 1 Comment
Figure S8, no boxed regions in the figure; instead of marking sub-cellular structures in the images with too many labels, it may be more helpful to indicate out the differences between wild type and KO species.

Authors comments: This was due to a mistake from editing the supplemental material during the course of the first revision – we corrected the mistake.

© 2023. Published by The Company of Biologists under the terms of the Creative Commons Attribution License (https://creativecommons.org/licenses/by/4.0/).

***** Reviewer 2 Advance Summary and Potential Significance to Field:
The authors have made several improvements in line with the previous reviews however a number of areas still have not been adequately addressed for publication and a number of the conclusions are still not properly supported by the data.

Reviewer 2 Comments for the Author:
Major comments

Chromatin condensation defect: No chromatin condensation defects are apparent in Fig S8.

Authors comment: We agree that the paragraph might have been confusing. Hence, we REMOVED the sentence referring to chromatin condensation in line 233 (pasted below).

~~"In part of the developing spermatids, chromatin condensation seems to be initiated earlier compared to WT spermatids. Darker stained chromatin could, however, also be a sign of DNA degradation."~~

Reviewer 2 Comment:
Meiosis being normal: the rationale to use stage I tubules to assess meiosis is unclear. Meiosis occurs over multiple stages with the meiotic divisions of meiosis I (metaphase/anaphase/telophase) and meiosis II occurring at stage XII, whereas prophase I spans stages I-XI. The only reason to assess stage I would be to observe if apoptotic metaphase/anaphase/telophase I and meiosis II cells stall into stage I, however that is not clear as written and stage XII should also be assessed to allow direct assessment of if the divisions are abnormal. A control image is also needed regardless for Fig S3E and a larger view of the stage I tubules as at present very few round spermatids can be seen.

Authors comment: In discussing the suggestions put forward by the reviewer, we tried to comply with the reviewer's suggestion for the first round of this revision, that is to include data on meiosis. Reading the above comment, we think that a concise evaluation is needed in order to draw firm conclusions about meiosis. However, to us, this is beyond the scope of the present manuscript, where we established ACTL7B deficient mice, went through omics-analyses to identify ACTL7B interaction partners like DYNLL proteins, and present results aimed to further validate the interaction. We would like to mention, that ACTL7B is localized to the cytoplasm and seemingly interacts with DYNLL1 and 2. We describe all phenotypic alterations directly linked to the loss of ACTL7B, that is defects which appear after the onset of expression of ACTL7B (in WT situation). Additionally, since ACTL7B is expressed in haploid spermatids only, we do not expect a direct effect of lack of ACTL7B on meiosis. Neither Clement et al. nor we found any indication or present any data, which would suggest a role of ACTL7B in meiosis. Further, previous literature did not show presence of ACTL7B in diploid cells in the testis, as we had quoted this in the Introduction: "In mice and humans, *ACTL7B* has been found to be expressed post- meiotically in round and elongating spermatids (Hisano et al., 2003, Guo et al., 2018)."
As a consequence, we decided to REMOVE Fig S3E and the referring sentence which is listed below.

~~As expected, meiosis was inconspicuous in *Actl7b-/-* testis and no increased numbers of apoptotic divisions were detected (Fig. S3E).~~

Reviewer 2 Comment:
Mislocalisation of mitochondria in knockout sperm tails: in describing Fig S6 the authors state that mitotracker signal was abnormally along the whole length of the tail in the knockout males. However, the mitotracker signals also appear along the whole tail of the WT and Het images presented and are comparable in that respect to the KO. The difference in the far right KO image wherein the mid-piece is clearly abnormal, however as the mitotracker signals overall appear non-specific, ultrastructure analysis via TEM or SEM of sperm tails is needed to verify if this is due to abnormal mitochondrial loading.

Authors response: We discussed the reviewers concern. We agree that we might have

© 2023. Published by The Company of Biologists under the terms of the Creative Commons Attribution License (https://creativecommons.org/licenses/by/4.0/).

overstated the finding and REMOVED the sentence previously in line 224-226 in order to clarify this section.

~~“In most *Actl7b-/-* sperm, PNA and Mitotracker signals were found along the whole tail and in the head region.”~~

In addition, the suggestion to perform TEM/SEM would need a high number of animals to be generated, due to the extremely low sperm count. Hence, such an experiment could be performed in the course of addressing further questions using the ACTL7B deficient mice and be included in a follow-up study.

Reviewer 2 Comment:
Cytoplasm eviction defect: is not clear in Fig 5 A and B. The rational to use these images to show eviction of cytoplasm is unclear as these stages seem to be well before spermiation, or are you referring to the single spermiated sperm in the knockout lumen in the lumen? If yes to the latter arrows are needed and an control appropriate control showing a spermiated WT sperm with not retained cytoplasm is needed.

Authors comment: The arrowhead was added. Due to concerns raised by reviewer 1, we re-worded the paragraph dealing with cytoplasm eviction. We might have overstated and overinterpreted the results. We amended the respective sections and watered the claim down and kept it on a descriptive level. It now reads (Line 210 ff):

Seemingly, in *Actl7b-/-* spermatids cytoplasm (labelled by cP2 staining) is retained. Transmission electron micrographs reveal that spermatids in *Actl7b-/-* testes show abnormal morphologies and excess cytoplasm (Fig. 5A). In comparison, in the lumen of *Actl7b+/+* tubules morphologically normal sperm line up to be spermiated (Fig. 5B). Staining against cP2 (to visualize cytoplasm) on caput epididymal sections produced a strong signal in immature germ cells in *Actl7b-/-* mice indicating the presence of cytoplasm (Fig. 5C).

Reviewer 2 Comment:
Formatting of figures and inclusion of control panels still needs attention:
1. Fig S3A panel is labelled as stage VI, in text is referred to as IX? Also, a control panel is needed for comparison to Fig. S3 A.

Authors comment: We discussed this issue as the staging of ACTL7B mutant testes is neither easy nor straightforward. We believe that the stage shown in the Fig. S3A reflects more stage III-IV containing arrested step 10 - 12 spermatids. This was changed in the panel title, legend as well as in the main text. Further a WT control is now added to Fig. S3A for comparison. Text in line 179 now reads:

Cohorts of step 10-12 elongating spermatids can be found it what appears to be a tubule in stage III-V (Fig.S3A). Figure legend in (Fig. S 3A) now reads:
Step 10-12 elongating spermatids are present in Actl7b-/- (vermillion arrow heads)

Reviewer 2 Comment:
2. Fig S3E as above control panel is needed for comparison
Authors comment: We removed the analysis regarding the meiosis and hence Fig. S3E is no longer needed. Reviewer 2 Comment:
3. Fig 3 as detailed above stage matched control images are needed for Fig 3A and 3B/C

Authors comment: WT images were added to Fig. 3A. For Fig. 3 B C we show the situation in the ACTL7B mutant testes, there is no comparable structure in WT, hence matched controls are not available.
Reviewer 2 Comment:
3. Fig S4 figure legend – panel numbers need to be corrected in legend and tubule stage should be included on images
Authors comment: Panel labels were corrected in legend and included the stages in the image. Reviewer 2 Comment:
4. Fig 4B - panels are not labelled with respective genotypes

© 2023. Published by The Company of Biologists under the terms of the Creative Commons Attribution License (https://creativecommons.org/licenses/by/4.0/).

Authors comment: Panels are now labelled.

Reviewer 2 Comment:
Minor comments

Sperm number - swim out is not a quantitative method. It biases against immotile sperm and also the number of sperm that vacate the epididymis can be influenced by the number of cuts made etc. Accurate quantification required homogenisation of the testis. That the swim out method was used both herein and in the Clements paper may contribute to the differences in results, in addition to the strain of the animals as the authors suggest.

Authors comment: We agree that a “swim out” might not be appropriate term. However, we are using more of a “flush out” procedure, which, in our hands lets us also isolate immotile sperm as well. We cut the cauda and flush vigorously using a pipette (1ml) with media in order to obtain all cells. In addition, the epidydimal sections shown in Fig. 2 F clearly indicate, that virtually no sperm is present in ACTL7B males confirming our data about the sperm count.

Reviewer 2 Comment:
Number of elongating spermatids per tubule cross section - please specify stage of seminiferous tubule used for this assessment and how many tubules assessed per mouse.

Authors Comment:
We amended the information to the material and methods section, it reads now (line 644) :

Five seminiferous tubule cross sections for 3 individuals each were counted as described previously (Merges at al. 2022). Cross sections of seminiferous tubes in stage I-III have been used.

***** Reviewer 3 Advance Summary and Potential Significance to Field:
This is an exciting paper examining the role of a testis specific Actin-related proteins (Arp), ACT7LB, a highly conserved protein in primates and rodents in spermiogenesis. ACTL7B is specifically expressed in round and elongating spermatids, and the Actl7b-KO mice are infertile and display defects in sperm formation, cytoskeleton organization, and chromatin condensation, leading to spermatid arrest or loss at various stages of the seminiferous tubule cycle. To better understand the role of ACT7LB the authors performed IP-MS and find that ACT7LB interacts with dynein protein, which implicate ACT7LB and Dynein’s in the global sperm remodeling process. This is an important body work for the field.

Reviewer 3 Comments for the Author:
The authors have done addressed many of the raised concerns. This is good for publication.

Authors comment: Thanks for the positive evaluation of our work.

Third decision letter

MS ID#: DEVELOP/2023/201593

MS TITLE: Actl7b-deficiency leads to mislocalization of LC8 type dynein light chains and disruption of murine spermatogenesis

© 2023. Published by The Company of Biologists under the terms of the Creative Commons Attribution License (https://creativecommons.org/licenses/by/4.0/).

AUTHORS: Gina Esther Merges, Lena Arevalo, Andjela Kovacevic, Keerthika Lohanadan, Dirk G. de Rooij, Carla Simon, Melanie Jokwitz, Walter Witke, and Hubert Schorle

I have now received all the referees' reports on the above manuscript, and have reached a decision. The referees' comments are appended below, or you can access them online: please go to BenchPress and click on the 'Manuscripts with Decisions' queue in the Author Area.

As you will see, the reviewer #1 seems not to be happy with your revision in the view point of interaction of ACTL7B with dynein components. I partially share her/his concern as the IP-western in physiological setup are not very strong, I must say. However, as you mentioned in the text, Mass Spec analysis shown in supplementary material should be more convincing. This MS data could be more carefully displayed as figures with p-values (such as volcano plots or something similar way). General view for ACTL7B-centric interactome could also be appreciated to capture how it works during spermiogenesis. My suggestion is to convert the MS data in supplementary table to more comprehensive figures and show them in the Figure 6. If you are able to revise the manuscript along the lines suggested, I will be happy receive a revised version of the manuscript. Your revised paper will be re-reviewed by myself, and acceptance of your manuscript will depend on your addressing satisfactorily the reviewer 1's and my major concerns. Please also note that Development will normally permit only one round of major revision. If it would be helpful, you are welcome to contact us to discuss your revision in greater detail. Please send us a point-by-point response indicating your plans for addressing the referee’s comments, and we will look over this and provide further guidance.

Please attend to all of the reviewers' comments and ensure that you clearly highlight all changes made in the revised manuscript. Please avoid using 'Tracked changes' in Word files as these are lost in PDF conversion. I should be grateful if you would also provide a point-by-point response detailing how you have dealt with the points raised by the reviewers in the 'Response to Reviewers' box. If you do not agree with any of their criticisms or suggestions please explain clearly why this is so.

Reviewer 1

Advance summary and potential significance to field

The authors of this manuscript found that ACTIN like protein ACTL7B plays important roles during the elongation stage of spermiogenesis. Using gene targeting in mouse, they found that male Actl7b homozygous mutants are infertile, possibly due to the drastic defects in cellular morphogenesis of haploid spermatids, which carried malformed nuclei and flagella. Using biochemical and proteomics methods, they found that ACTL7B may form complexes with dynein light chain proteins DYNLL1 and DYNLL2, suggesting an interesting link between cytoskeletal networks for the coordinated morphogenesis of sperm. Mutant testis contained varied levels of numerous proteins comparing to those of wild type and heterozygous controls.
Their results potentially add an important clue to the understanding of how spermatogenic cells coordinate complexed morphological changes during spermiogenesis and what molecular players may be involved in this process.

Comments for the author

The main concern is still on the protein interaction:
1) Mass spec identifies proteins in the IP complex, not necessarily proteins interacting directly;
2) If the co-IP samples are separated under denaturing conditions (SDS-PAGE and WB), proteins in a complex should be hard to still stay together, so the individual protein bands can be seen on blots. Thus, it is hard to conclude that the “60 KD” band, as suggested by the authors, to be complex of ACTL7B and DYNLL1/2, in Figure 6A and B (e.g. IP-D2 lane). This is the same question for the IP experiments did in 293T cells (Figure 6C).
3) The raw data in Figure S9A shows two blots hybridized with anti-ACTL7B and anti-DYNLL2 sequentially.
Since both antibodies are rabbit polyclonal according the information given in the Materials and Methods, this could cause a problem for the second hybridization, which likely show contamination from the first hybridization.

© 2023. Published by The Company of Biologists under the terms of the Creative Commons Attribution License (https://creativecommons.org/licenses/by/4.0/).

4) If the raw blots shown in Figure S9 are for Figure 6, their M.W. marker should be the same, is the 55 KD marker band mislabeled as 50 KD? Please draw clear lines to indicate each band in the M.W. lane and the target protein bands in the sample lanes when making figures. The raw blot shown in figure S9A, third panel (the anti-ACTL7B blot) appeared not the same one as the Figure 6A's anti-ACTL7B strip, please show the same raw blot.
5) In the main text and in the figures (Figure 6C), it is indicated that the ACTL7B expressed in 293T cells is the fusion protein of ACTL7B-GFP, which should have a c.a. M.W. around 72 KD, but the ACTL7B band shown in Figure S10 upper panels are around 55 KD. If the expressed protein is non-tagged version, how was GFP-trap beads used for IP experiments?
6) Co-localization experiments shown in Figure S13 and S14 may help to make the point. The data may suggest that overexpression of ACTL7B-GFP did something to change the nuclear localization DYNLL2 (the first panel of Merged should show red staining images for WT-HEK), however, it is less convincing than biochemical data in terms of detecting protein interactions at the resolution the authors used. The localization change seemed not so evident for DYNLL1. In addition, localizations of these proteins in HEK293 cells could be different from that of spermatids even in the wild type background.
7) Figure 7B WB shows the antibodies could identify clear bands of ACTL7B, DYNLL1 and DYNLL2, please show the full-length gel of these blots in supplements like the ones shown in Figure S9 and S10.
These problems need to be addressed. The authors should consider to use more specific antibodies such as those for Myc or Flag tags to conduct co-expression and Co-IP experiments, or use recombinant proteins (e.g. GST tagged) expressed in E. coli to conduct in vitro pull-down assays in order to show the direct interaction between ACTL7B and DYNLL2 or DYNLL1, since this is one of the main conclusions that may help to show additional information than the study that has been published earlier. In addition, Co-IP using two-step method (primary antibody, then protein-A/G beads) should be considered, instead of generating antibody coupled dynabeads.
Other minor points:
1) Lines128-129, since no phenotype was found for Act7lb heterozygous null mice, the staining used was not quantitative, it is not clear what the “gene-dosage effect” means;
2) What the positive control is in Figure S2H was not explained;
3) It may be a norm to present wild type figures in front of corresponding mutant figures, and as such, images in Figure 3A, S3 and 5A should be re-arranged and presented accordingly;
4) Lines 210-216, only Figure S5A shows TNP1 staining, not S5B; clear for S5B and S5C, which one is full-length PRM2 staining and which one is cP2 staining, in the figure labeling, figure legend and corresponding main text;
5) Lines 221-224, inconsistency in the main text description (referred to several structures) and the figure legend (referred to only condensed nuclei), cellular structures the authors want the readers to pay attention to should be indicated with labels in these images, and please provide wild type control images correspondingly;
6) Lines 230-233, not sure if the manchette is much different in the KO sample from that the wild type sample judging from Figure S6B;
7) Lines 252-255, it'll be helpful if the rationale for choosing DYNLL1/2 for further study is given out, while other proteins identified seemed more abundant. Can other proteins identified as potential positive hits imply on the functional roles of ACT7LB? This should be discussed.

Reviewer 2

Advance summary and potential significance to field

The authors have sufficiently addressed the concerns raised in my previous reviews.

Comments for the author

The authors have sufficiently addressed the concerns raised in my previous reviews.

© 2023. Published by The Company of Biologists under the terms of the Creative Commons Attribution License (https://creativecommons.org/licenses/by/4.0/).

Third revision

Author response to reviewers' comments

Please see the pdf named "rebuttal letter" uploaded as supplementary information.

Dear Dr. Koseki,

Please find enclosed the copy of your decision letter, where we have pasted our reply (in light blue) in a point by point fashion. Thanks for giving us the opportunity to revise the ms again. We appreciate your patience in this matter.

Best

H. Schorle

Dear Dr. Schorle,

I have now received all the referees' reports on the above manuscript, and have reached a decision. The referees' comments are appended below, or you can access them online: please go to BenchPress and click on the 'Manuscripts with Decisions' queue in the Author Area.

As you will see, the reviewer #1 seems not to be happy with your revision in the view point of interaction of ACTL7B with dynein components. I partially share her/his concern as the IP-western in physiological setup are not very strong, I must say. However, as you mentioned in the text, Mass Spec analysis shown in supplementary material should be more convincing. This MS data could be more carefully displayed as figures with p-values (such as volcano plots or something similar way). General view for ACTL7B-centric interactome could also be appreciated to capture how it works during spermiogenesis. My suggestion is to convert the MS data in supplementary table to more comprehensive figures and show them in the Figure 6. If you are able to revise the manuscript along the lines suggested, I will be happy receive a revised version of the manuscript. Your revised paper will be re-reviewed by myself, and acceptance of your manuscript will depend on your addressing satisfactorily the reviewer 1's and my major concerns. Please also note that Development will normally permit only one round of major revision. If it would be helpful, you are welcome to contact us to discuss your revision in greater detail. Please send us a point-by-point response indicating your plans for addressing the referee’s comments, and we will look over this and provide further guidance.

Authors Comment:
Dear editor. This is an interesting and very intriguing suggestion. We performed the CoIP MassSpec on testes tissue and (added in this version) on the HEK 293 cells overexpressing ACTL7B-GFP. Both tools allowed us to identify the DYNLL Proteins as interaction partners. In addition, using testes extract, we performed the “reverse”- IP DYNLL2 and detected ACTL7B as well (Fig. 6). DYNLL Proteins are expressed during spermiogenesis shortly after ACTL7B allowing this interaction. Loss of ACTL7B leads to altered localization of DYNLL Proteins in sperm (Fig. 7). Further, when ACTL7B is overexpressed in HEK 293 cells localization of DYNLL Proteins seem to “move” from mainly nuclear (in wt controls, Fig S13) to cytoplasmic. This was further corroborated co-transfecting ACTL7B-GFP and DYNLL1/2 mCherry (Fig S14).
What I like to say is, that in this manuscript, we did put a focus on the ACTL7B-DYNLL1/2 interaction. Aside from the Mass-Spec Data, we did not perform additional validation experiments on further proteins to feel confident enough to present and discuss a potential interaction. Hence, we would rather not display an interactome as it would be rather speculative.

Of note, after considering your and the reviewer comment, we concluded, that the Western-blot

© 2023. Published by The Company of Biologists under the terms of the Creative Commons Attribution License (https://creativecommons.org/licenses/by/4.0/).

analysis following CoIP using ACTL7B was misinterpreted from our side and we removed the dataset as well as the respective sentences in the manuscript. Since the ACTL7B-DYNLL interaction was detected in the MassSpec analysis, we hypothesize, that Mass-Spec Analyses are more sensitive and the detection level of the Western Blot was not reached.However, in the now edited Figure 6, we included the “reverse” CoIP using DYNLL1 and DYNLL2. There a band at 50kDa indicative for ACTL7B (although weak) is detected supporting the idea that both proteins interact.
Further in order to further support and validate our claim, we have performed an additional MassSpec analysis of material generated by pull down using ACTL7B-GFP overexpressed in HEK293 cells. Here, the analysis revealed DYNLL1 as interaction partner being enriched after ACTL7B-GFP pulldown (DYNLL2 on a weaker scale). We believe that this nicely adds to the data presented. The data are given as Supplementary Material 2 and we refer to it in the main text at line 263ff.

LINE 263:
Next, we generated HEK cells stably expressing *Actl7b* fused to *eGFP* ($HEK^{Actl7b\text{-}eGFP}$) and performed a pull-down with GFP nanobody-coupled beads followed by MassSpec analysis on the eluate. Here, we identified ACTL7B and DYNLL1 enriched in the Actl7b sample using a targeted analysis (Supplementary material 2). Of note, DYNLL2 peptides were detected but they were either identical with DYNLL1 or the detection level was low.

In addition, the expression pattern of Actl7b and Dynll genes are intriguingly overlapping (as indicated in Fig 1C and 7A) suggesting that they have the chance to interact during spermiogenesis. Further, the fact that ACTL7B expressed in HEK cells leads to relocation of DYNLL1 and 2 supports the idea of interaction. We think, all experiments together add up and back each other up. Also, we think that we choose our words carefully in order not to overstate the finding.
On a personal note, since the first author of the study (Merges) has finished the thesis and left the lab, right now, I simply do not have the manpower to perform more experiments in due time. This is a pity, because, we were the first to publish this findings (on biorxiv; December 19, 2022 https://www.biorxiv.org/content/10.1101/2022.12.19.520998v1.article-info) before submitting to Development. In the meantime (and presumably triggered by the biorxiv deposition by us), the competitors work describing Actl7b deficient mice was published in Biol. Reprod (DOI: 10.1093/biolre/ioad001) on March 13, 2023. We included and discussed the findings of this publication in our manuscript as requested from a reviewer in our previous revision. Being a reviewer for Development I do see the problem that the journal aims towards a nice and round story. Our manuscript might have its weaknesses since the Antibodies used are not the best and the molecules are sort of stubborn. If your decision would be “reject” would it still be possible under these circumstances to transfer to Biology Open and have some sort of accelerated review in order to get credit for the work done?

Please attend to all of the reviewers' comments and ensure that you clearly highlight all changes made in the revised manuscript. Please avoid using 'Tracked changes' in Word files as these are lost in PDF conversion. I should be grateful if you would also provide a point-by-point response detailing how you have dealt with the points raised by the reviewers in the 'Response to Reviewers' box. If you do not agree with any of their criticisms or suggestions please explain clearly why this is so.

Reviewer 1 Advance Summary and Potential Significance to Field:
The authors of this manuscript found that ACTIN like protein ACTL7B plays important roles during the elongation stage of spermiogenesis. Using gene targeting in mouse, they found that male Actl7b homozygous mutants are infertile, possibly due to the drastic defects in cellular morphogenesis of haploid spermatids, which carried malformed nuclei and flagella. Using biochemical and proteomics methods, they found that ACTL7B may form complexes with dynein light chain proteins DYNLL1 and DYNLL2, suggesting an interesting link between cytoskeletal networks for the coordinated morphogenesis of sperm. Mutant testis contained varied levels of numerous proteins comparing to those of wild type and heterozygous controls.
Their results potentially add an important clue to the understanding of how spermatogenic cells coordinate complexed morphological changes during spermiogenesis and what molecular players may be involved in this process.

© 2023. Published by The Company of Biologists under the terms of the Creative Commons Attribution License (https://creativecommons.org/licenses/by/4.0/).

Reviewer 1 Comments for the Author:
The main concern is still on the protein interaction:
1) Mass spec identifies proteins in the IP complex, not necessarily proteins interacting directly;

Authors Comment:
We are aware of the fact that Co-IP detects proteins in an IP complex. This does not render the observed interaction less relevant in a physiological context. On the contrary, it opens up research to determine the proteins of the proposed complex.

2) If the co-IP samples are separated under denaturing conditions (SDS-PAGE and WB), proteins in a complex should be hard to still stay together, so the individual protein bands can be seen on blots. Thus, it is hard to conclude that the “60 KD” band, as suggested by the authors, to be complex of ACTL7B and DYNLL1/2, in Figure 6A and B (e.g. IP-D2 lane). This is the same question for the IP experiments did in 293T cells (Figure 6C).

Authors Comment:
After considering your and the reviewer comment, we concluded, that the Western-blot analysis following CoIP using ACTL7B was misinterpreted from our side and we removed the dataset as well as the respective sentences in the manuscript. Since the interaction was detected in the MassSpec analysis, we hypothesize, that Mass-Spec Analyses are more sensitive and the detection level of the Western Blot was not reached.
However, in the now edited Figure 6, we included the “reverse” CoIP using DYNLL1 and DYNLL2. There a band at 50kDa indicative for ACTL7B (although weak) is detected supporting the idea that both proteins interact.
However, in order to further support and validated our claim, we now performed an additional MassSpec analysis of material generated by pull down using ACTL7B-GFP overexpressed in HEK cells. Here, the analysis revealed DYNLL1 as interaction partner being enriched after ACTL7B-GFP pulldown (DYNLL2 on a weaker scale). We believe that this nicely adds to the data presented. The data are given as Supplementary Material 2 and we refer to it in the main text at line 266ff.
3) The raw data in Figure S9A shows two blots hybridized with anti-ACTL7B and anti-DYNLL2 sequentially. Since both antibodies are rabbit polyclonal according the information given in the Materials and Methods, this could cause a problem for the second hybridization, which likely show contamination from the first hybridization.

Authors Comment:
Due to the re-arrangement of Figure 6, where we now only present the data from the CoIP using DYNLL2 (IP D2) and DYNLL1 (IP D1) coupled beads, we no longer have the former S9A and B in the supplemental figures. It is now Fig S9. Using ACTL7B Antibody first, we detect a band approx. 50kDa, i.e. the correct size for Actl7B (left panel, green arrow). This means, that the CoIP using DYNLL2 as a bait pulled down ACTL7B. Using antiDynLL2 detects a band at 12kDa (middle and left, red arrow). This band is not detected in the left panel using ACTL7B AB. It appears, and we agree, that in the left panel a band can be seen at 50kDa as well (yellow arrow). This might indeed be a “bleeding through” artifact from the first AB (ACTL7B). However, the fact, that ACTL7B staining was performed first and produced the 50kDA band and no 12 kDa Band should be proof, that the IP using DYNLL2 is able to pull down ACTL7B (see below).

© 2023. Published by The Company of Biologists under the terms of the Creative Commons Attribution License (https://creativecommons.org/licenses/by/4.0/).

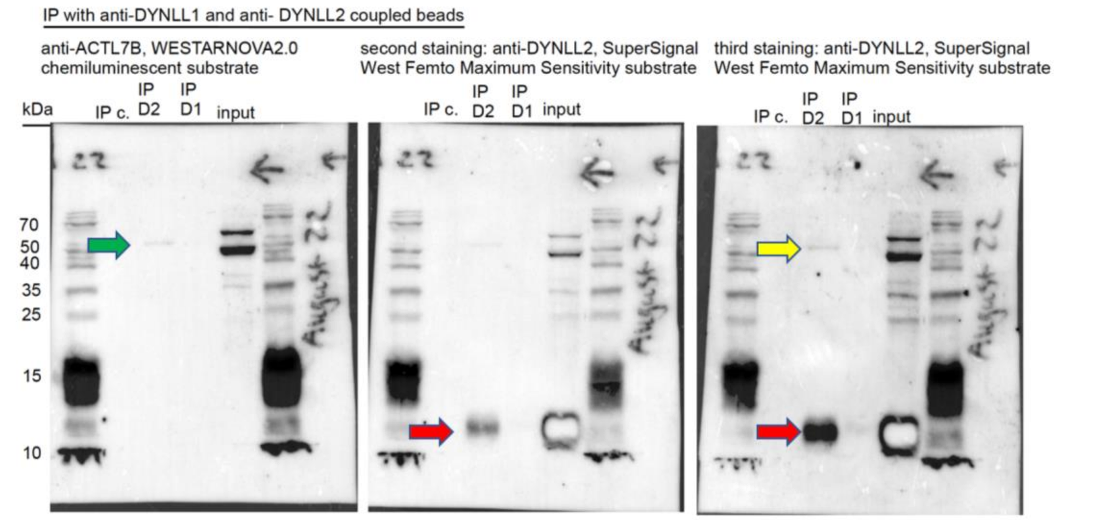

Fig. S9. Western blots of IPs using anti-DYNLL1 and anti-DYNLL2 coupled beads and bead only controls. Merged pictures of protein bands detected and colorimetric picture of the membrane with protein ladder. Note: The anti-ACTL7B antibody (13537-1-AP; Proteintech) has been shown to generate unspecific bands in western blot using mouse testis tissue subjected to SDS PAGE (see suppliers webpage). The specific band runs at a height of around 45 kDa.

4) If the raw blots shown in Figure S9 are for Figure 6, their M.W. marker should be the same, is the 55 KD marker band mislabeled as 50 KD? Please draw clear lines to indicate each band in the M.W. lane and the target protein bands in the sample lanes when making figures. The raw blot shown in figure S9A, third panel (the anti-ACTL7B blot) appeared not the same one as the Figure 6A's anti-ACTL7B strip, please show the same raw blot.

Authors Comment:
As pointed out above, Figure 6 A (and C) was removed.

5) In the main text and in the figures (Figure 6C), it is indicated that the ACTL7B expressed in 293T cells is the fusion protein of ACTL7B-GFP, which should have a c.a. M.W. around 72 KD, but the ACTL7B band shown in Figure S10 upper panels are around 55 KD. If the expressed protein is non-tagged version, how was GFP- trap beads used for IP experiments?

Authors Comment:
Of note, Figure 6 C was removed. However, we provide and explanation to the reviewers comment here: ACTL7B has a M.W. of 49KD, the GFP-M3 used here is 22 KD making a theoretical protein size of 71 KD. We rechecked and found a mistake in the labelling of the ladder. Due to the fact that we have both, the PAGE Ruler Plus™ and PAGE Ruler™ prestained protein ladders, there was a mixup. We labelled the ladder according to the PAGE Ruler but the PAGE ruler PLUS was used instead. PAGE Ruler Plus lacks the 40kDa band. Now the sizes have been corrected. As mentioned by the reviewer, the protein sizes now match the sizes calculated by the reviewer i.e. 49 and >70 kDa.

6) Co-localization experiments shown in Figure S13 and S14 may help to make the point. The data may suggest that overexpression of ACTL7B-GFP did something to change the nuclear localization DYNLL2 (the first panel of Merged should show red staining images for WT-HEK), however, it is less convincing than biochemical data in terms of detecting protein interactions at the resolution the authors used. The localization change seemed not so evident for DYNLL1. In addition, localizations of these proteins in HEK293 cells could be different from that of spermatids even in the wild type background.

Authors Comment:
We are aware of the fact, that the LC8 dynein light chains might have a different localization in HEK293 cells compared to spermatids. This is an additional experiment performed in the course of the revision and was used to address the question if localization of DYNLL 1/2 proteins is affected "per se" upon expression of ACTL7B in the cell system (HEK293 cells). We do not claim that change of localization seen in HEK cells are identical to what we see in sperm in our manuscript. The HEK293 wt cells stained for DYNLL in red merged with Hoechst (blue) are shown in the last

© 2023. Published by The Company of Biologists under the terms of the Creative Commons Attribution License (https://creativecommons.org/licenses/by/4.0/).

panel.

7) Figure 7B WB shows the antibodies could identify clear bands of ACTL7B, DYNLL1 and DYNLL2, please show the full-length gel of these blots in supplements like the ones shown in Figure S9 and S10.

Authors Comment:
We have included the blots as new supplementary figure S10. (see line 272).
Accordingly, all other supplementary figures were renamed both in the legend and the text.

These problems need to be addressed. The authors should consider to use more specific antibodies such as those for Myc or Flag tags to conduct co-expression and Co-IP experiments, or use recombinant proteins (e.g. GST tagged) expressed in E. coli to conduct in vitro pull-down assays in order to show the direct interaction between ACTL7B and DYNLL2 or DYNLL1, since this is one of the main conclusions that may help to show additional information than the study that has been published earlier. In addition, Co-IP using two- step method (primary antibody, then protein-A/G beads) should be considered, instead of generating antibody coupled dynabeads.

Authors Comment:
These experiments are certainly a good idea. We allow to disagree with the reviewer, that the interaction with DYNLL1/2 is the main conclusion. We also think that the concerns of the other reviewers were sufficiently addressed in the course of major revision. At this point, the proposed additional experiments would go beyond the scope of this revision and represent a starting point for further analyses aimed to fully understand the interactome of ACTL7B during spermiogenesis. This project will be then less developmental biology but much more biochemistry angled.

Other minor points:
1) Lines128-129, since no phenotype was found for Act7lb heterozygous null mice, the staining used was not quantitative, it is not clear what the “gene-dosage effect” means;

Authors Comment:
With the term ´gene dosage´ we simply refer to the definition of the ´amount of gene product´. In order to clarify the issue, we have deleted the term, since IHC is not an appropriate method to quantify protein levels. The new sentence reads (lines 124-125):
In testis sections of *Actl7b*+/- mice, ACTL7B signal appeared weaker.

We like to point out that we performed WB on WT, het and ACTL7B-/- testes and show the results in Fig 7B and S10 where we see a reduction of 7B levels in the het mice compared to WT testes (see line 272, Fig 7B and S10).

2) What the positive control is in Figure S2H was not explained;
Authors Comment:
We have re-written this sentence to make it more clear. We added the information to the figure legend (588 ff.) and figure legend to Fig. S2).

Positive controls were generated using DNase I treatment as recommended by the manufacturer of the kit used. The method section states: TUNEL assay was performed utilizing the TUNEL Assay Kit- HRP-DAB (Abcam, ab206386) according to manufacturer’s instructions. Positive control slides were generated by treatment with 1 µg/µl DNase I in TBS/ 1 mM $MgSO_4$ for 20 min at RT as recommended by the manufacturer.

3) It may be a norm to present wild type figures in front of corresponding mutant figures, and as such, images in Figure 3A, S3 and 5A should be re-arranged and presented accordingly;

Authors Comment:
Figure 3A does not show images of WT. These are displayed in Figure 2F, according to the discussion in the first round of revision. We are not aware of a rule to present WT images first. As requested in the first round of revision, we have added individual genotype labels to each image,

© 2023. Published by The Company of Biologists under the terms of the Creative Commons Attribution License (https://creativecommons.org/licenses/by/4.0/).

to make the inspection by the reader easier. We have arranged the figures according to the mentioning in the text. As far as we are aware, this is the proper way to present the results in figures.

4) Lines 210-216, only Figure S5A shows TNP1 staining, not S5B; clear for S5B and S5C, which one is full- length PRM2 staining and which one is cP2 staining, in the figure labeling, figure legend and corresponding main text;

Authors Comment:

The sentence the reviewer refers to reads as follows (line 207):

Transition proteins are loaded onto the DNA and later protamines are detected in the nuclei of spermatids (Fig. S5A-B).
So, the sentence refers to both TNPs and Protamines, which is why both S5A (TNP staining) and S5B (Protamine 2 staining) are referenced. S5B and S5C are both stainings against the full-length (i.e. unprocessed Protamine2) using an Antibody developed and published by us.
The antibody targeting cP2 detects the full length PRM2, which still contains the cP2 part. We demonstrated previously that neither cP2 alone (since it is degraded) nor mature Prm2 is detected by the cP2-specific antibody (Arévalo *et al*. 2022, Merges *et al*. 2022). This AB is used to check to check for eviction of cellular components to the residual bodies.

5) Lines 221-224, inconsistency in the main text description (referred to several structures) and the figure legend (referred to only condensed nuclei), cellular structures the authors want the readers to pay attention to should be indicated with labels in these images, and please provide wild type control images correspondingly;

Authors Comment:
The figure legend (Figure 5) reads: (D-G) Representative images of Actl7b-/- spermatids with condensed nuclei. Scales: 2µm.
This is meant to indicate the type of cell we are looking at. Defects are described in the text.
We have answered to this in the first round of revision and provide our respective answers below (in red):

Point 3) made by Reviewer 3: In figure 5 including reference panels for WT and also using similar magnification for WT/Mutant cell comparisons is necessary to make informed decisions by reviewer.

Our answer: Thank you for this comment. We realized that we need to be clearer at this point of the manuscript. We added the genotypes to Fig. 5. We did not add an additional WT image for Fig. 5C-F (now: Fig.5D-E), since WT spermatid development is depicted in Fig. S7 and Fig. S8.

Point 15) made by Reviewer: Fig 5A, please include appropriate control comparison images.

Our answer: Fig. 5B is the control image to Fig. 5A. Fig. 5B is a WT image. We added labels of the genotype in the figure to be clearer.

Point 7) made by Reviewer 2: More care needs to be taken with the formatting of many of the figures. Please include seminiferous tubule stage and genotype on all figures. For many of the EM images, the authors should crop them appropriately i.e., to focus specifically on the structure/cell of interest.

Our answer: We have added the stages of tubules depicted were we thought its necessary and re-checked with an expert in the field to ensure all staging was done correctly. The genotypes are given directly in the figure or the figure legends. Since various new stainings and new confocal images were added to the manuscript during the revision process, we think that all defects described are shown in sufficient detail. Further, we think that the TEM images used for staging the spermatids (Fig. S7 and S8) serve the reader best, when the whole cell is visible.

Point 2) of minor concerns made by Reviewer 1: 2. Lines 174-175, Fig. 5A: how is the “eviction of

© 2023. Published by The Company of Biologists under the terms of the Creative Commons Attribution License (https://creativecommons.org/licenses/by/4.0/).

cytoplasm” different from wild type? The genotypes of mice used should be indicated on the graphs for better readability. A control image (wild type) may be included for data shown in Fig. 5C-F. In this figure and Fig. 3B-C and Fig. S4, what are the dark granules that look like gold particles used for immuno-staining?

Our answer: Thank you for this comment. We realized that we need to be clearer at this point of the manuscript. We added the genotypes to Fig. 5 and added a staining against cleaved protamine 2 to discuss the eviction of cytoplasm. See lines 218-221: Staining against cP2 (to visualize residual cytoplasm) on caput epididymal sections showed, that large amounts of cP2 are retained in the cytoplasm of immature germ cells in Actl7b-/- mice (Fig. 5C). This again suggests defects in the eviction of cytoplasm.We did not add an additional WT image for Fig. 5C-F (now: Fig.5D-E), since WT spermatid development is depicted in Fig. S7 and Fig. S8. The dark granules on the TEM pictures shown do not originate from amino gold stainings. As far as we are aware these are liposomes and degrading RNAs. We see these granules on all TEM images taken regardless of the genotype or mouse line analyzed.

6) Lines 230-233, not sure if the manchette is much different in the KO sample from that the wild type sample judging from Figure S6B;

Authors Comment:
The staining of the manchette was an additional experiment performed in the course of the revision, as asked for by Reviewer (point 14):
Lines 181-182 ‘a subset of spermatids lacks the manchette, several spermatids show partly detached ectoplasmic F-Actin bundles’. This is not clear in the presented images. Higher magnification is needed. The manchette phenotypes should be confirmed with IHC of tubulin on testis sections.

Our answer was: The sentence has been deleted and the images of alpha-tubulin stained manchette structures have been added to the manuscript. Here we focus on those spermatids isolated from testis that showed manchette structures (Fig. S6B), since absence of manchette structures are most likely caused by spermiogenic arrest or spermatid degradation. The following statement has been added to the manuscript (lines: 228-233):
Of note, Clement et al. reported mislocalization of mitochondria in their Actl7b-KO model (Clement et al., 2023). Clement et al. further describe mislocalization of flagellar proteins in KO sperm and multiple morphological malformations of flagellae. Therefore, we used IC staining against α-tubulin to analyze manchette formation in Actl7b-/- spermatids. Manchette appear irregular compared to WT and Actl7b-/- spermatid head shapes are abnormal (Fig. S6B).

In sum, we believe that we were very careful with our wording, staining that manchette “appear irregular”. Since, Reviewer 2 requested these changes and was satisfied with our approach and answer, we believe this to be sufficient.

7) Lines 252-255, it’ll be helpful if the rationale for choosing DYNLL1/2 for further study is given out, while other proteins identified seemed more abundant. Can other proteins identified as potential positive hits imply on the functional roles of ACT7LB? This should be discussed.

Authors Comment:
This point was raised and addressed in the first round of revision as well (please find the answer below):

.

Point 5) of minor concerns made by Reviewer 1: Lines 206-208, only DYNLL1/2 is mentioned, what are the other proteins identified as positive hits in the Co-IP experiments?

Our answer: We have added two sentences to this part of the manuscript to mention other proteins identified and to justify why we concentrated our analysis on the LC8 dynein light chains. See lines 252-258:
Further, a published bead proteome from HeLa cells was used to filter out proteins which nonspecifically bind Dynabeads (Trinkle-Mulcahy et al., 2008). Those included various H2B histone variants. In the Co-IP using the anti-ACTL7B-coupled beads, we identified LC8 light chains, Dynein light chain 1 (DYNLL1) and its paralog dynein light chain 2 (DYNLL2). Other proteins identified

© 2023. Published by The Company of Biologists under the terms of the Creative Commons Attribution License (https://creativecommons.org/licenses/by/4.0/).

were ribonucleoproteins and ribosomal proteins, which were excluded from further analysis.

We would like to stress the point, that we do not claim that DYNLL proteins are the only interaction partners of ACTL7B. But from the data generated, looking at the expression pattern from DYNLL1 and DYNLL2 during spermiogenesis it appeared to be the most interesting and logic thing to do (considering the phenotype described). Certainly, it would be interesting to try to unravel the whole interactome in a separate study.

***** Reviewer 2 Advance Summary and Potential Significance to Field: The authors have sufficiently addressed the concerns raised in my previous reviews.
Reviewer 2 Comments for the Author:
The authors have sufficiently addressed the concerns raised in my previous reviews.

Fourth decision letter

MS ID#: DEVELOP/2023/201593

MS TITLE: Actl7b-deficiency leads to mislocalization of LC8 type dynein light chains and disruption of murine spermatogenesis

AUTHORS: Gina Esther Merges, Lena Arevalo, Andjela Kovacevic, Keerthika Lohanadan, Dirk G. de Rooij, Carla Simon, Melanie Jokwitz, Walter Witke, and Hubert Schorle
ARTICLE TYPE: Research Article

I am happy to tell you that your manuscript has been accepted for publication in Development, pending our standard ethics checks.

© 2023. Published by The Company of Biologists under the terms of the Creative Commons Attribution License (https://creativecommons.org/licenses/by/4.0/).
